# Prognostic risk factors of serous ovarian carcinoma based on mesenchymal stem cell phenotype and guidance for therapeutic efficacy

**DOI:** 10.1186/s12967-023-04284-3

**Published:** 2023-07-11

**Authors:** Xiaohui Yang, Minying Zheng, Yidi Ning, Jie Sun, Yongjun Yu, Shiwu Zhang

**Affiliations:** 1 Nankai University School of Medicine, Nankai University, Tianjin, 300071 People’s Republic of China; 2 Department of Pathology, Tianjin Union Medical Center, Tianjin, 300121 People’s Republic of China

**Keywords:** Serous ovarian carcinoma, Mesenchymal stem cell, Prognosis model, Immunochemistry, Metastasis

## Abstract

**Background:**

Epithelial ovarian cancer is the leading cause of death from gynecologic cancer, in which serous ovarian carcinoma (SOC) is the most common histological subtype. Although PARP inhibitors (PARPi) and antiangiogenics have been accepted as maintenance treatment in SOC, response to immunotherapy of SOC patients is limited.

**Methods:**

The source of transcriptomic data of SOC was from the Cancer Genome Atlas database and Gene Expression Omnibus. The abundance scores of mesenchymal stem cells (MSC scores) were estimated for each sample by xCell. Weighted correlation network analysis is correlated the significant genes with MSC scores. Based on prognostic risk model construction with Cox regression analysis, patients with SOC were divided into low- and high-risk groups. And distribution of immune cells, immunosuppressors and pro-angiogenic factors in different risk groups was achieved by single-sample gene set enrichment analysis. The risk model of MSC scores was further validated in datasets of immune checkpoint blockade and antiangiogenic therapy. In the experiment, the mRNA expression of prognostic genes related to MSC scores was detected by real-time polymerase chain reaction, while the protein level was evaluated by immunohistochemistry.

**Results:**

Three prognostic genes (PER1, AKAP12 and MMP17) were the constituents of risk model. Patients classified as high-risk exhibited worse prognosis, presented with an immunosuppressive phenotype, and demonstrated high micro-vessel density. Additionally, these patients were insensitive to immunotherapy and would achieve a longer overall survival with antiangiogenesis treatment. The validation experiments showed that the mRNA of PER1, AKAP12, and MMP17 was highly expressed in normal ovarian epithelial cells compared to SOC cell lines and there was a positive correlation between protein levels of PER1, AKAP12 and MMP17 and metastasis in human ovarian serous tumors.

**Conclusion:**

This prognostic model established on MSC scores can predict prognosis of patients and provide the guidance for patients receiving immunotherapy and molecular targeted therapy. Because the number of prognostic genes was fewer than other signatures of SOC, it will be easily accessible on clinic.

**Supplementary Information:**

The online version contains supplementary material available at 10.1186/s12967-023-04284-3.

## Background

Ovarian cancer (OC) is one of the most dangerous gynecologic malignancies. In 2020, the morbidity of OC was estimated to be 3.4% worldwide for women, and ranked eighth among female malignant tumors and third among gynecological malignancies. Mortality from OC accounts for 4.7% of all female malignant tumors around the whole world and is ranked second among female genital tumors [1]. Serous ovarian cancer (SOC) is the most common type of gynecological malignancy [2]. The National Comprehensive Cancer Network (NCCN) has recommended poly ADP-ribose polymerase inhibitors (PARPi) and angiogenesis inhibitors as treatment for patients with SOC. Although PARPi and antiangiogenics have been shown to prolong progression-free survival (PFS), they do not reflect an obvious improvement in overall survival (OS) of patients with SOC [3, 4].

The tumor microenvironment (TME) denotes the niche where tumor cells interact with the surrounding stroma, including various immune cells, stroma cells, lymph-vascular space, and the extracellular matrix (ECM) [5]. Mesenchymal stem cells (MSCs) are a key component of stromal cells and can mediate the immune response by inhibiting the activity of T lymphocytes, interfering with the proliferation and differentiation of B lymphocytes, and inducing macrophage phenotypic switching [6]. In addition to immune regulation, MSCs can promote angiogenesis by releasing soluble factors and can be a source of carcinoma-associated fibroblasts (CAF). CAFs, in turn, can directly release angiogenesis-related factors and indirectly modulate pathophysiological processes, including ECM stiffness, elasticity, and interstitial fluid pressure [7, 8]. In OC, MSCs regulate cancer cell proliferation, metastasis, phenotype, and response to chemotherapy by binding directly to target cells, secreting soluble factors, or discharging exosomes from MSCs [9–11]. Cancer-associated MSCs (CA-MSCs) can be isolated and identified in tumor tissue, and exhibit a unique gene expression profile from MSCs compared to that of healthy individuals [12]. Patients with the CA-MSC phenotype have a significantly worse PFS than those with the normal MSC phenotype [10]. It is reported that CA-MSCs of an immune 'hot' mouse OC drived CD8 + T cell tumor immune evasion of CD8 + T cells from tumors and these mouse exhibited a poor response to anti-programmed death ligand 1 (PD-L1) immune checkpoint blockade therapy (ICB) through the secretion of multiple chemokines, such as CCL2, CX3CL1, and TGF-β1 [13].

Cobalt chloride (CoCl_2_)-induced polyploid giant cancer cells (PGCC) exhibit the same characteristics as cancer stem cells (CSC) and express markers related to CSC (CD133 and CD44). Daughter cells produced by PGCC undergo an epithelial–mesenchymal transition (EMT) and gain a mesenchymal phenotype and are thus endowed with strong abilities for migration and invasion [14], thus promoting cancer progression [15–17]. And the risk score of PGCCs and their daughter cells with high migration and invasion capacity were higher than those of control cells.

In this study, a comprehensive transcriptomic analysis of MSC trait genes was performed. Using an MSC-related scoring system based on the expression of 3 MSC genes, patients were divided into low- and high-risk groups. Compared to the low-risk group, the high-risk group had a worse prognosis and exhibited an obvious immunosuppressive effect. Analysis of genomic data and molecular targeted therapy datasets between the two groups revealed that high-risk patients had less homologous recombination deficiency (HRD) and were weakly responsive to anti-PD-1 inhibitors. The prognosis of patients who received traditional chemotherapy was worse than that of patients treated with both bevacizumab and chemotherapy in high-risk group. In addition, the mRNA of PER1, AKAP12, and MMP17 were highly expressed in normal ovarian epithelial cells compared to OC cell lines and the protein levels were increased in SOC cases with metastasis.

## Methods

### Dataset collection, filtering, and preprocessing

Transcriptome profile value (counts; Fragments Per Kilobase Million [FPKM]) and matched clinical information on OC were downloaded from The Cancer Genome Atlas (TCGA) database (https://portal.gdc.cancer.gov/) and were used for the training cohort. The validation datasets were retrieved from two different microarray platforms in Gene Expression Omnibus (GEO), including GSE26712, GSE14764, GSE23554, GSE17260, and GSE53963. The gene expression profile of normal ovary tissue was obtained from the Genotype-Tissue Expression (GTEx) database from the University of California Santa Cruz (UCSC) Xena browser (https://xenabrowser.net/hub/). The exclusion criteria for OC samples were: (i) duplicate samples; (ii) nonserous tumors; and (iii) samples without follow-up record.

The normalized FPKM count matrix and clinical data from patients with advanced melanoma treated with Nivolumab (anti-PD-1) in GSE91061 were available to define MSC risk scores for immune checkpoint blockade therapy. GSE140082, a study of patients with OC treated with bevacizumab (an antiangiogenetic agent), was tested for the application of the MSC risk scores and response to antiangiogenic therapy. Variables with a *P-*value < 0.1 were considered.

Raw data from the Affymetrix platform were filtered using the gcRMA algorithm and adjusted for background and quantile normalization based on the Affy software package (Affymetrix, Inc) [18]. The 'backgroundCorrect' and 'normalizeBetweenArrays' functions in the limma package used performed for the Agilent platform data sets for normalization of data [19]. The ComBat algorithm of the “sva” package corrected for batch effects and non-biological technical biases across different data sets [20]. For RNA sequencing data, the FPKM value was transformed into a transcript per million (TPM) value and was further log2- transformed (log2TPM) for subsequent analyzes.

### Access to gene signatures

MSCs have the capabilities of self-renewal and multidirectional differentiation. A total of 19 MSC-related gene signatures were extracted from the GOBP_MESENCHYMAL_STEM_CELL_DIFFERENTIATION and GOBP_MESENCHYMAL_STEM_CELL_PROLIFERATION in Molecular Signatures Database (MSigDB) (Additional file 1). For angiogenesis-related genes, a gene set was derived from the intersection of several published papers investigating angiogenesis in OC and from the HALLMARK_ANGIOGENESIS in MSigDB [21–25] (Additional file 2). The immune gene signatures of melanoma and the formula of immune gene score were from the literatures (Additional file 3). The immune gene score: ${\sum }_{i=1}^{29}\mathrm{\beta i}*\mathrm{Xi}$, βi is the estimated regression coefficient of each gene and Xi is the expression value of each gene.

### Single-sample gene set enrichment analysis (ssGSEA) and immune or stromal cell infiltration in TME

Gene expression values for samples were rank-normalized and enrichment scores were aggregated using the Empirical Cumulative Distribution Function (ECDF) of the genes in the signatures. The enrichment scores represent the relative abundance of the gene sets in all samples and were obtained using the ssGSEA algorithm of the R package 'GSVA' [26]. 

xCell is a published method based on ssGSEA that estimates abundance scores of 64 types of immune cells and stromal cells, including adaptive and innate immune cells, hematopoietic progenitors, epithelial cells, and ECM components [27]. Fourteen kind of tumor-infiltrating lymphocytes were identified from xCell, including activated B cell, activated CD4 T cell, activated CD8 T cell, CD56-bright natural killer cell, CD56-dim natural killer cell, gamma delta T cell, immature B cell, natural killer T cell, natural killer cell, regulatory T cell, T follicular helper cell, type 1 T helper cell, type 17 T helper cell and type 2 T helper cell.

### Identification of differentially expressed genes (DEGs)

The patients were classified into two groups according to enrichment scores of` MSC infiltration (MSC scores). With the absolute value of the fold change > 1 and a false discover rate (FDR) < 0.05 as the screening conditions, 7792 significant genes of these two clusters were identified with the R package 'edgeR' [28]. For DEGs between normal ovaries and tumor tissues, the 'limma' package was used to distinguish 7269 genes from the count matrix in the context of the absolute value of fold change > 2 and a FDR < 0.05 [19].

### Analysis of MSC-related pattern function and pathway enrichment

Genes in different MSC clusters were subjected, respectively, to Gene Ontology (GO) enrichment and Kyoto Encyclopedia of Genes and Genomes (KEGG) pathway analysis using the 'clusterProfiler' package in R software. The statistical significance criteria for the enrichment analysis were set as an adjusted *P-*value of < 0.05.

### Weighted correlation network analysis (WGCNA) of MSC-score genes

WGCNA is a computational method in systems biology that describes the correlation patterns among genes and can be used to summarize changes between the gene set and phenotype [29]. The Log_2_TPM of 7269 genes was specified as the input data, and the soft threshold power of β = 6 was selected to construct the scale-free topology network and gene modules. A correlation analysis between the constructed modules and MSC scores was performed and a significant module was adopted for subsequent analyses. The entire process was performed using the R package 'WGCNA'.

### Establishment of an MSC score gene-related prognostic model

The univariate Cox regression analysis was performed using 245 genes extracted from the Brown module to identify prognostic genes with the criterion of *P* < 0.05. A three-MSC-related gene-score prognostic model was constructed using the stepwise regression analysis derived from multivariate Cox regression analysis. All samples in the training and validation cohorts were stratified into the low- or high-risk group according to the same formula of risk scores with the 'predict' function using a uniform cut-off value. To eliminate numerical differences between microarray data and high-sequence data, prognostic genes were centralized and standardized with the 'scale' function. Cox regression and Kaplan–Meier analyses were performed using the 'survival' R package. To evaluate the discrimination of the MSC score model, receiver operating characteristic (ROC) curve, area under the curve (AUC), and concordance index (C-index) were quantified using survivalROC package and survcomp package, respectively.

### Correlation of MSC scores and mesenchymal-related genetic characteristics

Mesenchymal-related characteristics, including neoantigen load, homologous recombination defects (HRD), CTA scores, intratumor heterogeneity (ITH), and copy number variation (CNV), were recovered from the study by Mariathasan et al. [30]. TCGA cohort gene variant information was accessible from the TCGAbiolinks package in R [31]. The package maftools was used to calculate the tumor mutation burden (TMB) and to define the mutational landscape of the 20 main driver genes with the highest mutation frequency [32]. TMB of GSE91061 was downloaded from the supplementary file of the corresponding literature (Additional file 4). The TISIDB (http://cis.hku.hk/TISIDB/index.php) is a reciprocal network demonstrating tumor and immune system interaction [33]. Twenty-four immunosuppressors from the TISIDB website were obtained for immunosuppressive analysis.

### Independence of MSC-based prognostic model and other clinicopathological parameters

Univariate Cox analysis was performed for each clinicopathological parameter to determine whether it was significantly associated with survival. Variables with a *P-*value < 0.1 were added as input from the multiple Cox regression and detected the association with quality of life. Cox analysis was implemented using the 'survival' package in R.

### Nomogram construction based on independent prognostic factors

Based on the contribution of each influencing factor on survival, certain scores for each influencing factor were obtained. The total scores of all factors were calculated to evaluate for the probability of OS at 1, 3, and 5 years for patients using the R package rms (version 6.3-0; https://CRAN.R-project.org/package=rms). The ROC curve and C-index were used to evaluate nomogram discrimination using the survivalROC package and the survcomp package, whose values fluctuate between 0.5 and 1. The consistency of the nomogram was determined using calibration plots with the calibrate function in the rms package. Decision curve analysis (DCA) was used to assessing the clinical impact of risk prediction models with or without other clinical indexes [34].

### Cell culture

The human normal ovarian epithelial cell line IOSE 80 and human OC cells HEY and SKOv3 were obtained from the Tianjin Union Medical Center and cultured under complete Dulbecco’s modified Eagle’s medium (DMEM) and RPMI-1640 conditions, respectively. The induction of PGCCs is undertaken when HEY and SKOv3 cells reached 60% confluence in T25 flasks [14, 17]. After treatment with 450 μM CoCl_2_ (Sigma-Aldrich, St. Louis, MO, USA) 3–4 times, cancer cells are harvested for further studies.

### Total RNA extraction and real-time polymerase chain reaction (RT-PCR)

Cell pellets were mixed with TRIzol and chloroform for lysis and RNase inactivity. After centrifugation at low temperature, isopropanol, and anhydrous ethanol were added to precipitate and wash RNA sediments, respectively. The concentration of RNA was calculated and 1000 μg RNA was used for reverse transcription to cDNA (11141ES10, Yeasen Biotechnology, Shanghai, China). RT-PCR was performed using the SYBR Green Master Mix (11184ES03, Yeasen Biotechnology, Shanghai, China). The relative gene expression was calculated using the Livak method (also known as the 2^−ΔΔCt^ method). All experiments were performed in triplicate. The primer sequences are listed in Additional files 5 and 6 and the protocol for reverse transcription and RT-PCR is previously described [35, 36]. The risks cores of cancer cell line are defined by the relative expression of each gene and its associated Cox coefficient: PER1 × 0.124154 + AKAP12 × 0.145457 + MMP17 × 0.122203.

### Tissue microarray and SOC samples

Paraffin-embedded SOC (n = 42) tissue samples were collected from the Department of Pathology in Tianjin Union Medical Center, and included 24 primary lesions without metastasis and 18 primary SOC with metastasis. The Hospital Review Board of the Tianjin Union Medical Center approved this study and patient information confidentiality was maintained.

### Immunohistochemistry (IHC) staining and scoring

Paraffin-embedded tissue sections were deparaffined with xylene and rehydrated in an ethanol gradient. After antigen retrieval with ethylenediaminetetraacetic acid (EDTA) buffer (Solarbio, Beijing, China) and blocking endogenous peroxidase, sections were then incubated with specific anti-PER1 (1:100, Proteintech, Wuhan, China), anti-AKAP12 (1:100, Proteintech, Wuhan, China), MMP17 (1:100, Proteintech, Wuhan, China) at 4 °C overnight. The sections were incubated with reagents 2 and 3 (PV-9001; Zhong Shan Biotech Co Ltd, Beijing, China) next day and detailed processes are provided in Additional file 7 [37, 38]. The staining intensity was assessed as follows: 0, negative; 1, light yellow; 2, yellow; and 3, brown. The percentage of positive cells was graded as 0 (< 10%), 1 (11–25%), 2 (26–50%), 3 (51–75%), 4 (76–100%). The multiplication of the staining intensity and positive cell scores was used to determine the staining index for each section.

### Statistical analyses

Data were analyzed using R version 4.1.2 (https://www.r-project.org/) and Bioconductor software (https://www.bioconductor.org/), a toolkit based on the R language. X-tile 3.6.1 software (Yale University, New Haven, CT, USA) was applied to determine the best cutoff value for patients with OC according to the MSC scores and were classified as low- and high-score groups [39]. SPSS 25.0 (IBM Corporation, Armonk, NY, USA) was used to analyze IHC data in the study. The expression of three prognostic genes was performed through UCSC XenaShiny, which is a package in R that can be downloaded to visualize datasets from the UCSC Xena database [40]. The Mann–Whitney–Wilcoxon test was used to compare two independent groups for continuous variables and ordinal categorical variable. The Spearman rank correlation coefficient was calculated to measure the dependence of two variables. Pearson correlation analysis were applied for detecting the correlation between metastasis and staining index. The Kaplan–Meier curves and the logarithmic rank test were used to assess the predictive ability of the prognostic model.

## Results

### Clusters were constructed based on the abundance scores of mesenchymal stem cells

**Fig. 1:**
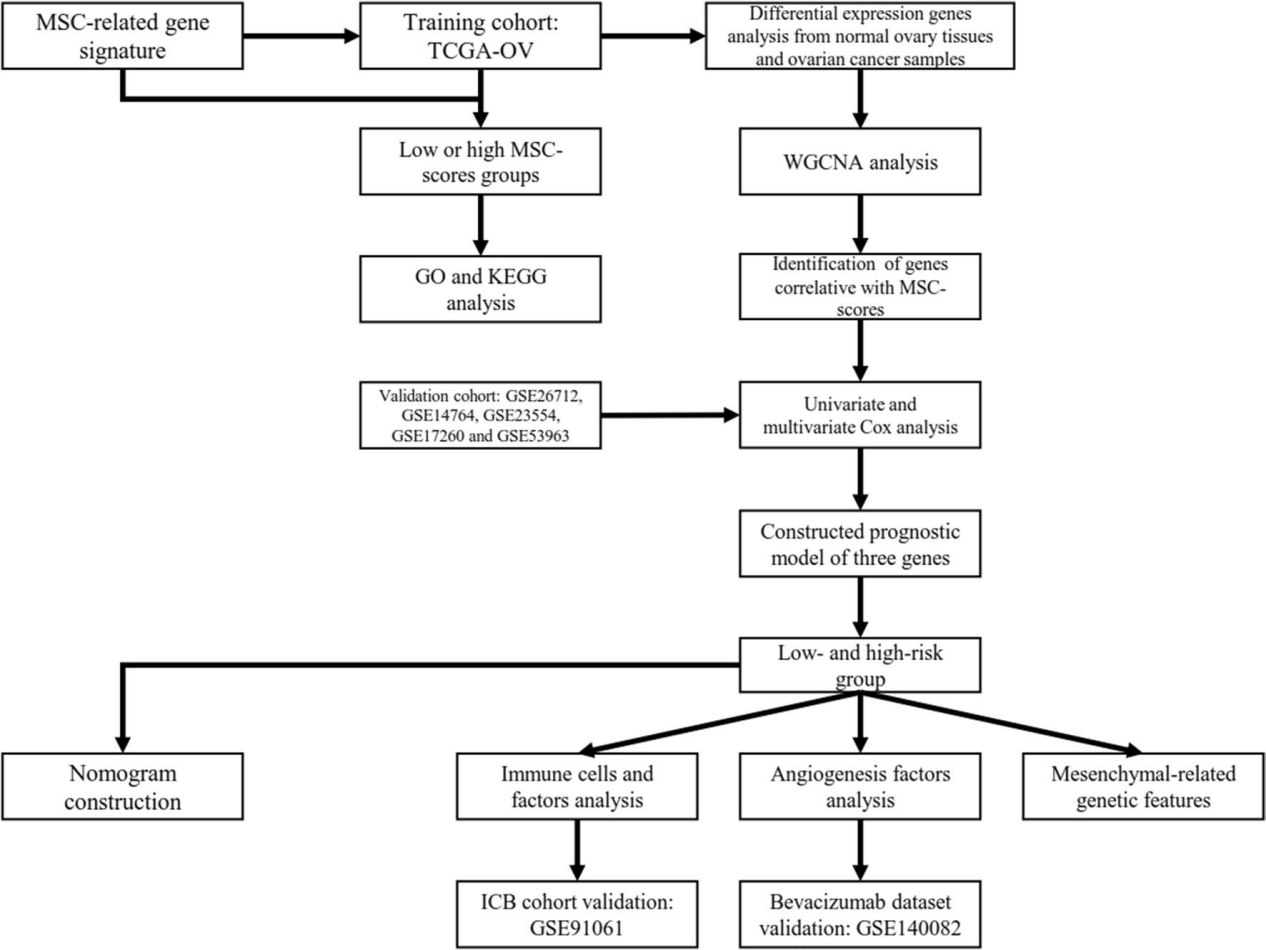
Graphical abstract of bioinformatics section in this study. *MSC* mesenchymal stem cell, *TCGA *the Cancer Genome Atlas, *WGCNA* weighted gene co-expression network analysis, *GO* gene ontology, *KEGG* Kyoto Encyclopedia of Gene Genome, *ICB* immune checkpoint blockade

**Fig. 2:**
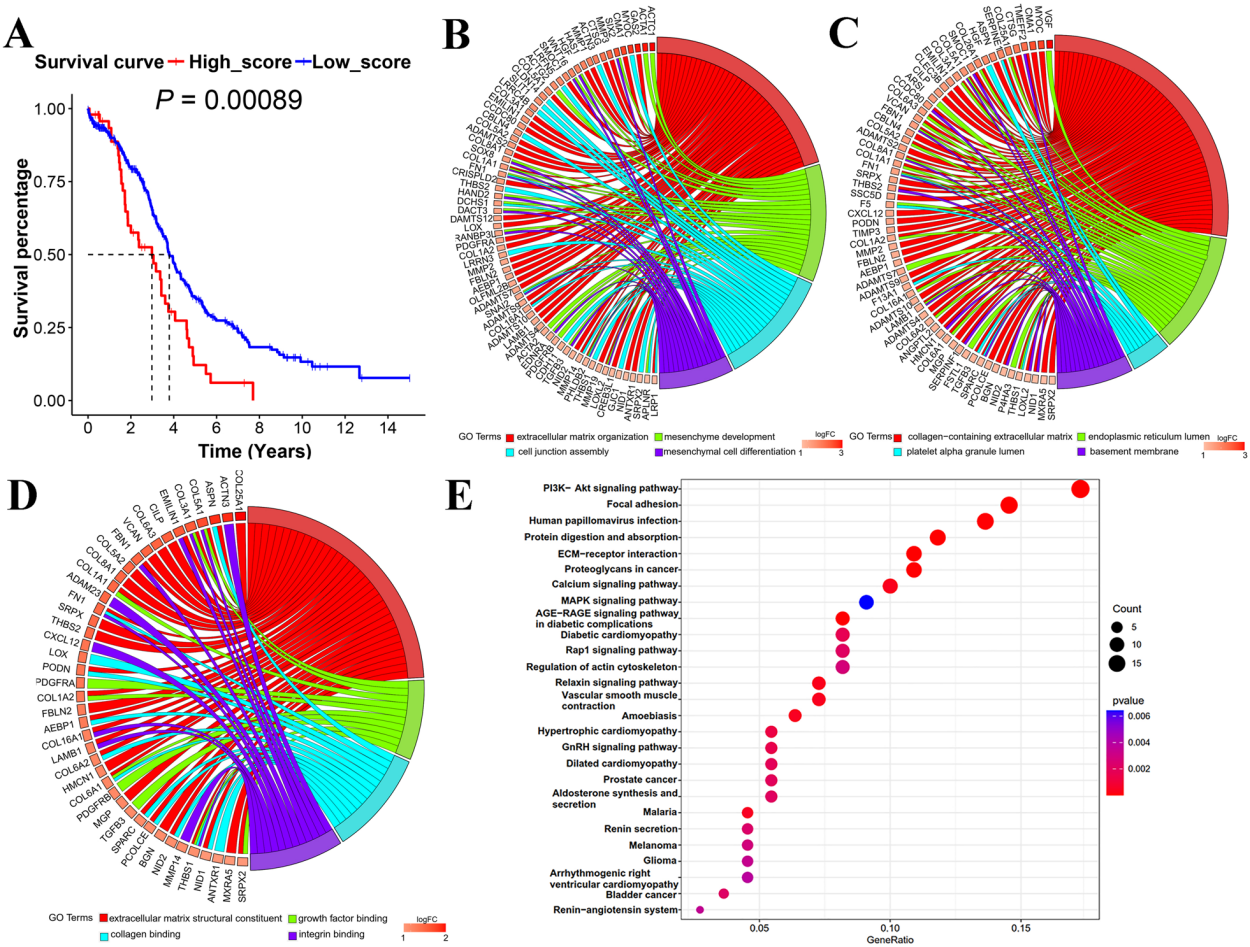
Enrichment analysis of genes related to MSC scores in TCGA-OV cohort. **A** Kaplan–Meier analysis of low and high MSC-scores groups. The most significant GO enrichment analysis of DEGs between groups with low and high MSC scores: **B** biological process (BP); **C** cellular components (CC); **D** molecular functions (MF). **E** KEGG pathway analysis of low and high MSC-scores groups. A larger circle represents more genes enriched in the corresponding term, and the darker rectangle means more statistically significant. *MSC* mesenchymal stem cell, *GO* gene ontology, *DEGs* differentially expressed genes

The general study flow is shown in Fig. 1. To better understand the association between the characteristics of MSCs and clinical characteristics of patients, the enrichment scores of OC cases were calculated based on 19 MSC-related gene signatures (Additional file 1) using ssGSEA methods. After removing duplicates, the expression profile of 376 samples was incorporated into the study. The normalized enrichment scores of each sample combined with survival information were considered input variables in the X-tile software (Additional file 8). The patients were then assigned to low or high MSC scores groups at the cut-off value of 0.7946 to identify the importance of MSC scores in predicting patient outcomes (Additional file 16: Fig. S1). The Kaplan–Meier analysis demonstrated that high MSC scores reflected poorer prognosis (Fig. 2A), while patients with a lower MSCs score were associated with an unfavorable prognosis.

### Identification and enrichment analysis of differential expression genes between the two MSC clusters

Using the package 'edgeR', there were 7792 genes identified between the low and high MSC scores groups using a filter criterion of |fold change|> 1 and FDR < 0.05, that included 4698 downregulated DEGs and 3094 upregulated DEGs. The top 25% DEGs of |fold change|> 2 were inserted into the function and the pathway enrichment analysis. GO was used to define gene or protein function in three dimensions: biological processes (BP), cellular components (CC), and molecular functions (MF). In terms of BP, the genes were enriched in the organization and stucture of ECM and MSC differentiation processes, such as the regulation of chondrocyte differentiation or the muscle system process (Fig. 2B; Additional file 9). With regard to CC terms, the collagen-containing ECM, endoplasmic reticulum lumen, and basement membrane, were detected (Fig. 2C; Additional file 9). For the MF analysis, the structural components of the ECM, collagen binding, and growth factor binding were significant processes (Fig. 2D; Additional file 9). As shown in the enrichment analysis, these differentially expressed genes were mainly correlated with mesenchymal development, differentiation, and relevant constituents and organelles, such as fibrin involved in the organization of the ECM or the active endoplasmic reticulum. According to the KEGG pathway analysis, the biological process involved in the ECM of cancer comprised ECM-receptor interaction and proteoglycans. The signal pathways associated with CSC included PI3K/Akt and MAPK signaling pathway. The PI3K/Akt signaling pathway involved in the regulation of chemoresistance and CSCs in ovarian carcinoma, and the MAPK signaling pathway can maintain CSC stemness in solid tumors [41, 42]. (Fig. 2E; Additional file 10).

### WGCNA co-expression network construction and significant module identification

**Fig. 3:**
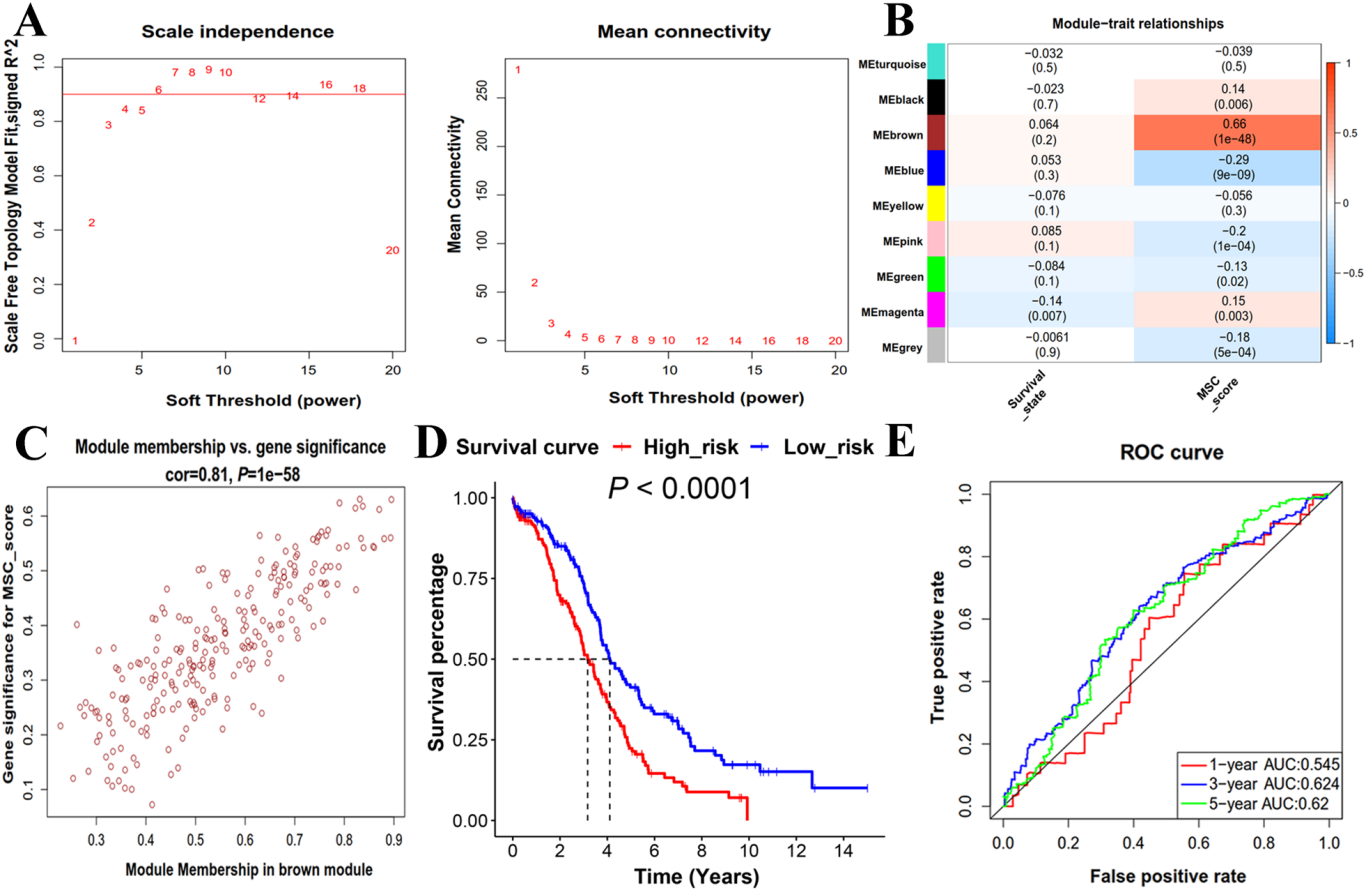
Identification of the set of MSC-scores-related genes in TCGA-OV cohort. **A** Soft threshold (β) filtering that includes scale independence and mean connectivity. Scale-free topological fitting index was set as 0.9, and the first number to reach 0.9 is the soft threshold (β = 6). When β = 6, the network connectivity is suitable and keep stable. **B** Correlation between module eigengenes and clinical traits (survival status and MSC-scores). The color of bar represents the correlation coefficient, and the value in bracket are *P* value. **C** Scatter plot of the module eigengenes related to the MSC-scores in the brown module. **D** Kaplan–Meier analysis of low- and high-risk score groups in TCGA-OV cohort. **E** The 1-year, 3-year and 5-year discrimination index (ROC curve and AUC values) of the prognostic risk model in TCGA-OV cohort. *ROC* receiver operating characteristic, *AUC* area under the curve

The differentially expressed genes were distinguished from 88 normal ovary tissues and 374 SOC samples. To identify a gene set that correlated with the MSC scores, 7269 DEGs derived from the above analysis were inserted in the WGCNA. After removing outliers, an appropriate soft threshold (β = 6) was chosen to construct a scale-free network that was validated by a value of R-square 0.93 (Fig. 3A, Additional file 16: Fig. S2A, B). Setting the cut off value at 0.25, the blue and red module eigengenes obtained were combined (Additional file 16: Fig. S2C, D). Afterwards, these modules were associated with MSC scores and survival. As shown in Fig. 3B, there was a strong correlation between the brown module and MSC scores. A total of 245 genes were identified in this module and the correlation coefficient of the brown module and the MSC scores reached 0.81 (Fig. 3C).

### Building the MSC-related gene score prognostic model

To further explore the relationship between MSC phenotypes and clinical traits, univariate Cox regression analysis was used to select genes related to prognosis, based on Brown module. Thirteen of 245 genes (*P* < 0.05) related to prognosis were finally defined as candidate genes to define the MSC score (Additional file 11). Multivariate Cox regression analysis was conducted to identify genes significantly associated with OS and three genes (MMP17, AKAP12, and PER1) were used to construct the prognostic model (Additional file 12). In accordance with risk scores obtained using the 'predict' function, the patients were classified into the low-risk and high-risk prognostic groups. Subsequently, Kaplan–Meier analysis showed that the group with a high MSC-related risk score had poorer prognosis than a low risk score (Fig. 3D). To verify model prediction efficiency, the ROC curve and AUC values were adopted, which presented a predictive advantage for the 3- and 5-year AUC for OS (Fig. 3E). The C-index, another discriminatory power for prediction models, characterized this model as less accuracy, with a value of 0.58 (Additional file 13). The gene expression heatmap, the distribution of risk scores, and the OS of TCGA-OV patients are shown in Additional file 16: Fig. S3A.

### Validation of the MSC prognostic model using GEO datasets

**Fig. 4:**
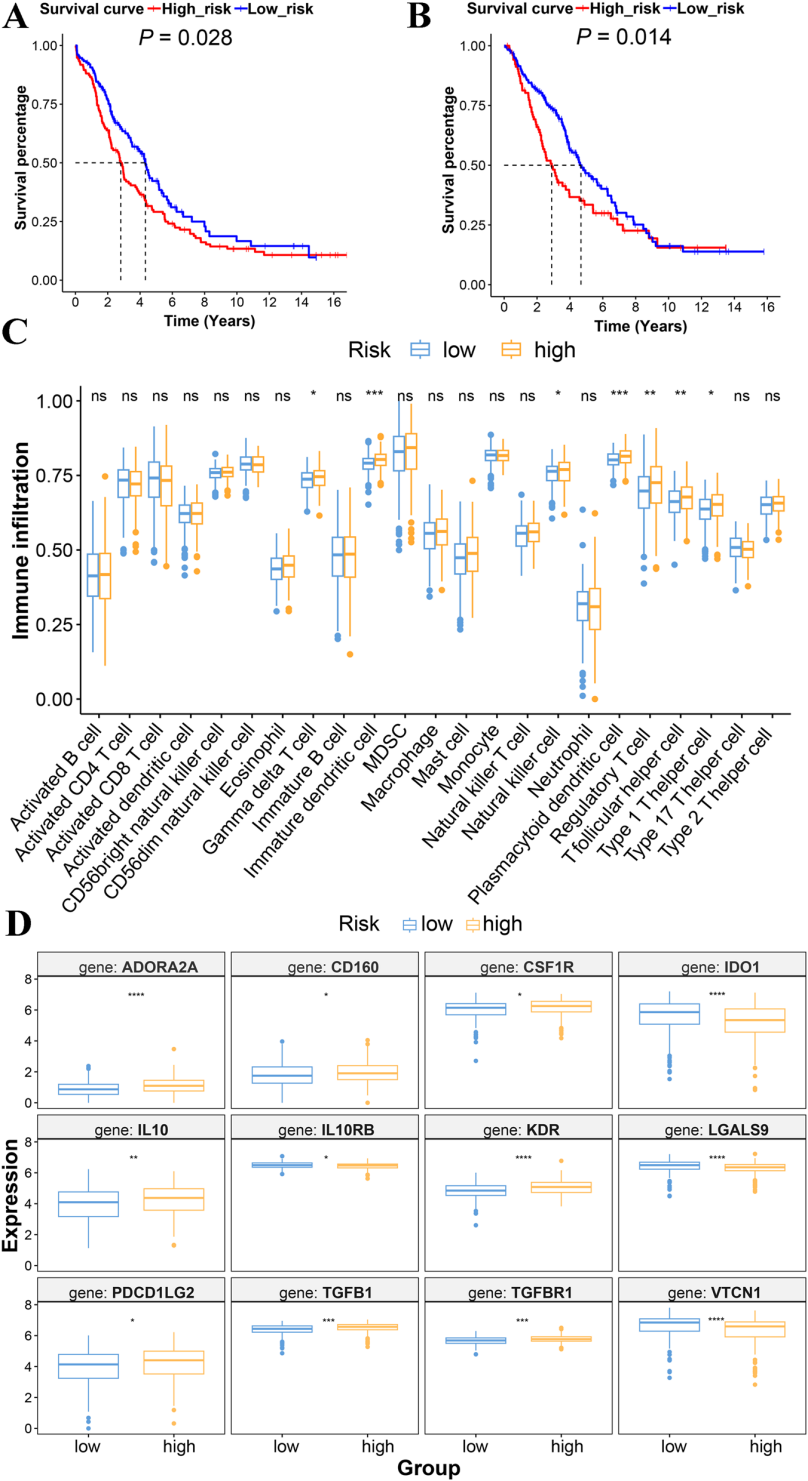
Validation of prognostic model in GEO datasets and immuno-related analysis in TCGA-OV cohort. **A** Kaplan–Meier analysis of low- and high-risk score in GSE17260 and GSE53963 of GPL6480. **B** Kaplan–Meier analysis of low- and high-risk score in GSE26712, GSE14764, and GSE23554 from GPL96.  (**C**) Abundant infiltration of immunocytes and (**D**) expression of immunosuppressive factors with statistic difference in TCGA-OV cohort. *P < 0.05, **P < 0.01, ***P < 0.001, ****P < 0.0001

To determine the consistency of this prognostic gene signature, a total of 565 samples were extracted for validation from five different datasets. There were significant differences in survival advantages between the low- and high-risk groups, which were in consistent with that in the TCGA cohort (Fig. 4A, B). Furthermore, the 3- and 5-year AUC were slightly higher than the 1-year AUC in the Affymetrix datasets, indicating superior long-term survival (Additional file 16: Fig. S3C). However, the 5-year AUC value in the Agilent data was lower than the 1- or 3-year AUC (Additional file 16: Fig. S3B). According to the results of AUC, the C-index indicated that the model in the validation cohorts had relatively low veracity (Additional file 13). Additional file 16: Fig. S3D, E show that cases with high expression of MMP17, AKAP12, and PER1 were characterized by high risk and poor prognosis.

### Immune or stromal cell infiltration signature in diverse grouping

MSC can also act as an immunosuppressor in cancers by releasing soluble factors in the TME [43], which may be related to a poor prognosis of the mesenchymal phenotype of SOC. For different MSC phenotypes, high-risk scores indicated the presence of abundant infiltration of immunocytes with inhibitory activity, such as immature dendritic cells and regulatory T cells (Fig. 4C). Except for tumor-associated immunosuppressive cells, immunity inhibition factors were also differentially expressed (Fig. 4D; Additional file 16: Fig. S4A). MSCs have been reported to secrete immunomodulatory factors that influence other immune or stromal cells, such as transforming growth factor-beta (TGF-β1) on macrophages, vascular endothelial growth factor receptor 2 (*KDR*) in endothelial cells*,* and galectin-9 (*LGALS9*) in T cells [44–46].

### Variable mesenchymal-related genetic characteristics

**Fig. 5:**
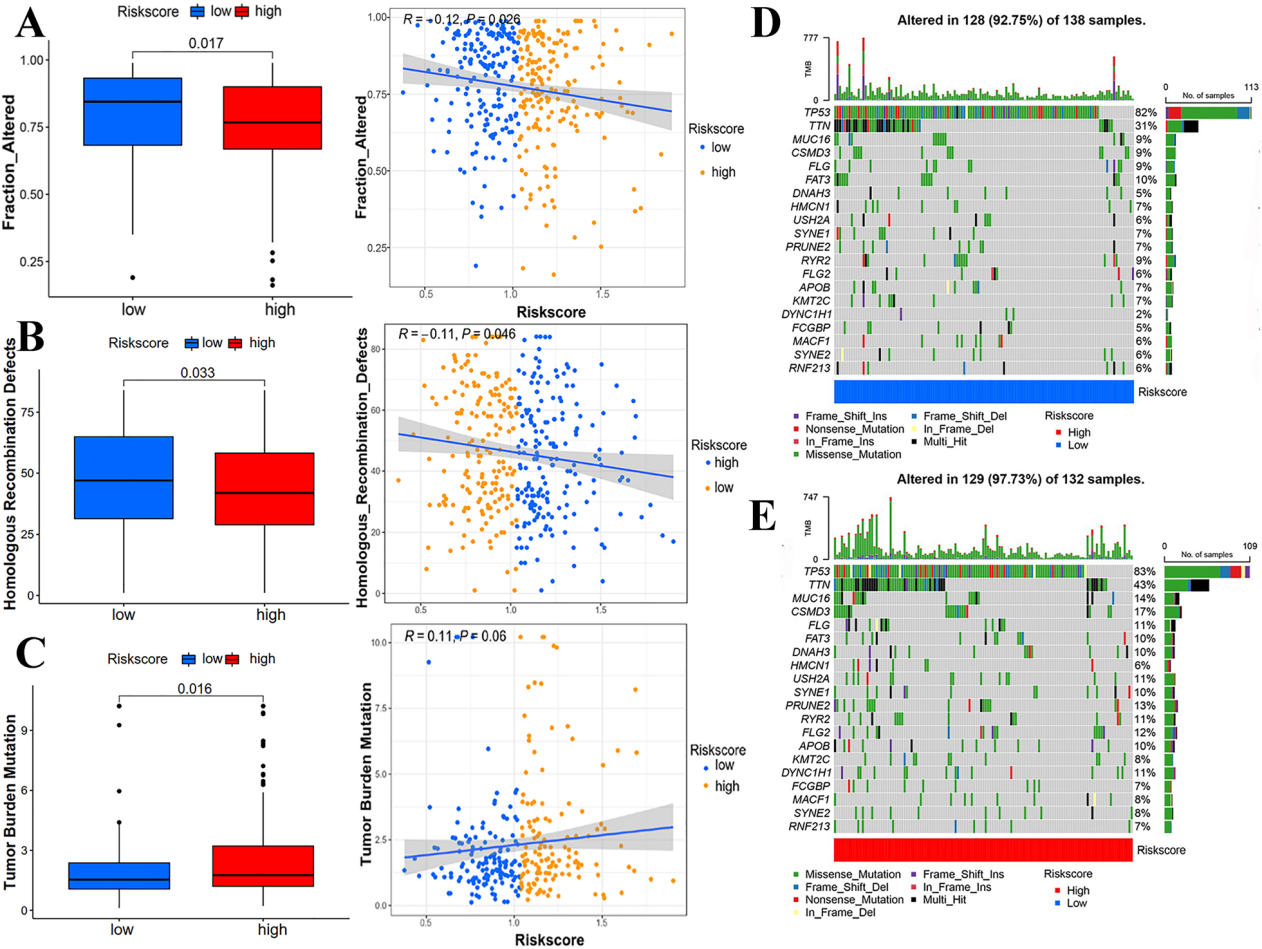
Genetic characteristics with statistical significance and correlation with risk score in TCGA-OV cohort: **A** Fractional changes of copy number variation (CNV). **B** Homologous recombination defects (HRD). **C** Tumor mutation burden (TMB). *R* means correlation coefficient. The top 20 mutant genes in the (**D**) low-risk group and (**E**) high-risk group

To explore latent genomic variants present in each risk groups, CNV, HRD, TMB, neoantigen load, CTA scores, and ITH were studied. According to a meta-analysis of tumor immune expression signatures [30], the CNV included both the number of segments and the fraction of genome alterations, both of which showed a higher frequency in the low-risk group (Fig. 5A; Additional file 16: Fig. S4B). HRD provided an opportunity to optimize the use of PARPi for the treatment of patients with high-grade SOC. In our scoring system, there was a negative correlation between risk scores and HRD (Fig. 5B). Unlike HRD, high MSC-related risk scores corresponded to more robust TMB (Fig. 5C). Subsequently, an explicit evaluation of the top 20 mutant genes of ovarian neoplasm was conducted for the risk score subgroups. As shown in Fig. 5D–E, the high-risk group tended to have more mutations commonly reported for OC. However, there was no statistical difference between the other three genetic parameters and the risk scores (Additional file 16: Fig. S4C–E).

### Application of the prognostic model in immune checkpoint blockade cohort

**Fig. 6:**
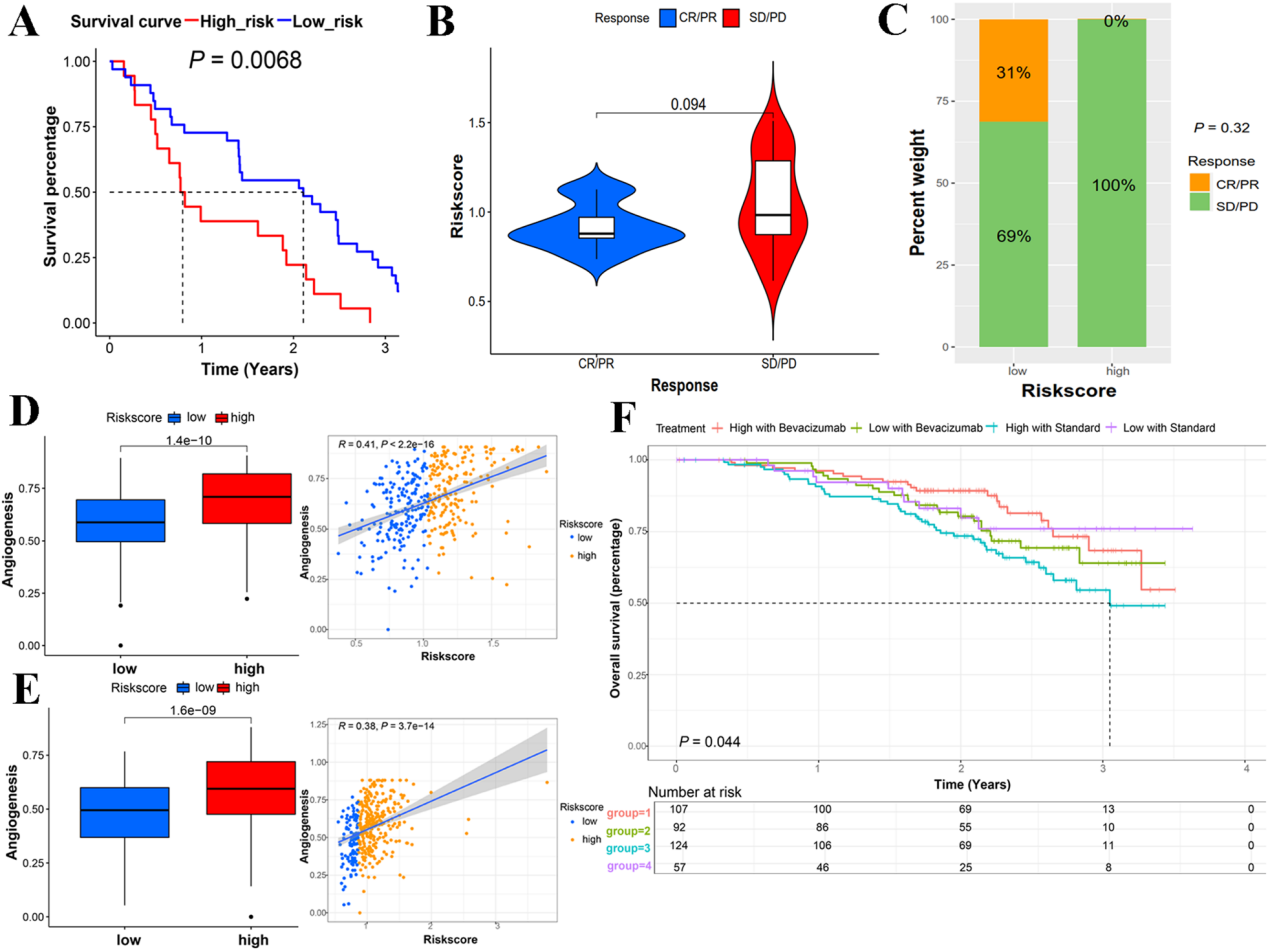
Application of MSC-related prognostic model in therapy cohorts. **A** Kaplan–Meier analysis of low- and high-risk score in GSE19061. **B** The risk scores of different response groups of GSE19061. **C** The proportion of patients with response to anti-PD-1 immunotherapy in different risk groups. **D** Expression of pro-angiogenic factors in the TCGA cohort. **E** Expression of pro-angiogenic factors in GSE140082. **F** Overall survival in GSE140082 with different treatments. High: high-risk group; Low: low-risk group. Standard treatment: carboplatin plus paclitaxel chemotherapy. Bevacizumab treatment: carboplatin plus paclitaxel chemotherapy plus Bevacizumab. *CR* complete response, *PR* partial response, *SD* stable disease, *PD* progressive disease

Anti-PD-1/PD-L1 antibodies have been included in the therapeutic guidelines for some cancers, including melanoma, non-small cell lung cancer, and clear cell renal carcinoma [47]. To validate this model in response to immune therapy, 51 patients with advanced melanoma who received anti-PD-1 therapy were considered in the assessment [48]. Using a universal calculation formula, the samples were given specific scores and were stratified into two groups. The Kaplan–Meier analysis revealed that the high-risk score group exhibited poor OS outcomes (Fig. 6A; Additional file 16: Fig. S5). With regard to the therapeutic response, patients who responded to treatment had a tendency for lower risk scores and a larger number of non-responders to treatment were in the high-risk group, indicating that the low-risk group was more likely to benefit from anti-PD-1 therapy (Fig. 6B, C). To illustrate the merit of the prognostic model based MSC-score, the efficacy of PD-L1 or PD-1 expression status, tumor-infiltrating lymphocytes (TILs), mutational burden, and immune gene signatures, which previously described as biomarkers for anti-PD-1 treatment, was also evaluated in GSE91061. PD-1 expression, 8 kinds of TILs and tumor mutational burden are capable of predicting the overall survival of SOC (Additional file 16: Fig. S6). PD-1 expression, 8 kinds of TILs and tumor mutational burden did not show better predictive power than MSC-score-based clustering for the evaluating PD-1 therapy response (Additional file 16: Fig. S7).

### Evaluation of the prognostic model of MSC scores in the patients treated with bevacizumab

Except for their immunosuppressive activity, MSCs can enhance angiogenesis by producing high levels of growth factors or cytokines that stimulate angiogenesis [43]. In TCGA training set, samples with high MSC risk scores had a higher proportion of gene expression associated with proangiogenic factors (Fig. 6D). The specific correlation of MSCs with angiogenesis was evidenced in the dataset treated with bevacizumab and the expression pattern were similar to that of TCGA cohort. (Fig. 6E; Additional file 16: Fig. S8A). Furthermore, patients with high MSC risk scores and who had received standard chemotherapy (carboplatin plus paclitaxel) were associated with a significantly worse prognosis than the other three groups (Fig. 6F), denoting that compared to the chemotherapeutics and bevacizumab combination, conventional chemotherapy alone achieved a poor prognosis in patients with the mesenchymal phenotype, therefore, the addition of bevacizumab to chemotherapy should be considered.

### Nomogram construction to identify predictive prognostic factors

**Fig. 7:**
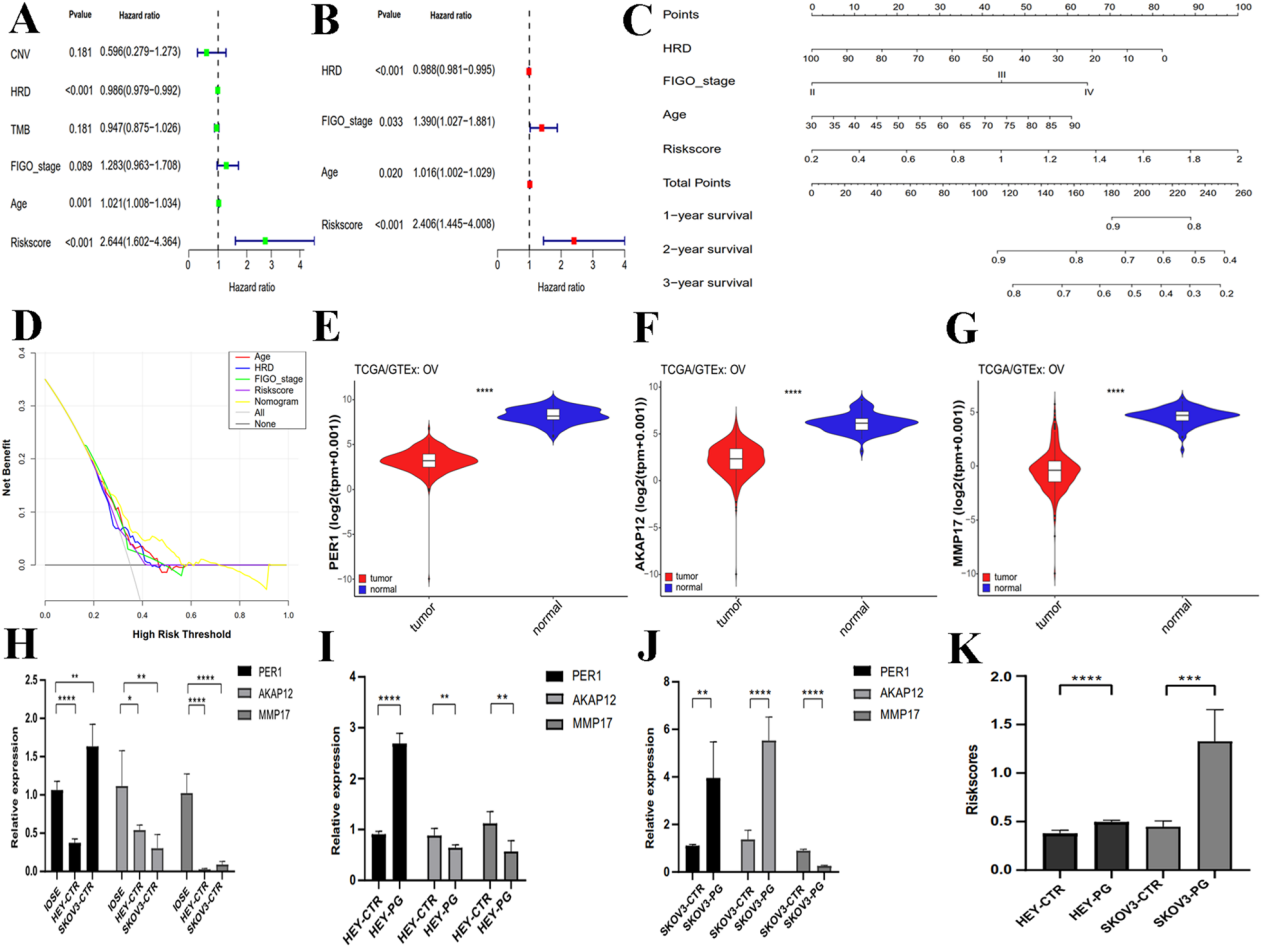
Construction of the nomogram of SOC-related characteristics in TCGA-OV cohort and validation of the expression of PER1, AKAP12 and MMP17. (**A**) Univariate Cox analysis and (**B**) multiple Cox regression on clinicopathological parameters. **C** The nomogram of independent parameters, including risk score, age, FIGO stage and HRD score to predict 1-, 3-, and 5-year OS of SOC. **D** Consistency validation of the nomogram with DCA. **E–G** The expression of PER1, AKAP12, and MMP17 between cancer and normal ovary in the TCGA and GTEx data set. **H** Comparison of the amount of PER1, AKAP12, and MMP17 between IOSE80 and the OC cell lines HEY and SKOv3. **I**, **J** Relative content of PER1, AKAP12, and MMP17 in HEY and SKOv3 cell lines and their PGCCs. **K** Risk scores for HEY and SKOv3 cancer cells and their PGCCs. *PER1* period circadian regulator 1, *AKAP12* a-kinase anchoring protein 12, *MMP17* matrix metallopeptidase 17, *DCA* decision curve analysis, *TCGA* the Cancer Genome Atlas, *GTEx* Genotype-Tissue Expression. *P < 0.05, **P < 0.01, ***P < 0.001, ****P < 0.0001

To explore the independence of the risk model, the 'survival' program was used to show that the MSC-related model was robust in predicting OS in patients with SOC (Fig. 7A, B). The nomogram with four variables (HRD, FIGO stage, age, and MSC risk scores) was constructed to provide guidance on prognosis of patients with SOC. Patients with higher HRD and risk scores, later FIGO stage, and older age, had a lower probability of long-term survival (Fig. 7C). The ROC curve and the C-index showed that the discrimination of this nomogram obviously increased under the application of the prognostic model containing MSC scores (Additional file 16: Fig. S8B; Additional file 13). The calibration plots show a prominent conformity between the nomogram prediction and actual observation in terms of the 1- and 3-year survival rates in the cohort (Additional file 16: Fig. S8C–E). The clinical impact with DCA showed that the nomogram had the highest net benefit at threshold probabilities between 0 and 60% (Fig. 7D), demonstrating this nomogram superiority over a single prognostic factor.

### Expression of PER1, AKAP12 and MMP17 genes using quantitative real-time polymerase chain reaction

On comparison of the expression of PER1, AKAP12, and MMP17 in TCGA with that in the GTEx dataset, the levels of the three genes were higher in normal ovarian tissues than in cancer tissues (Fig. 7E–G). RT-PCR was performed on human normal ovarian epithelial cells and human OC cells. The result confirmed that AKAP12 and MMP17 were overexpressed in IOSE 80 cells, while PER1 expression is highest in SKOv3 cells (Fig. 7H).

### Expression of prognostic genes in human SOC tissues

**Fig. 8:**
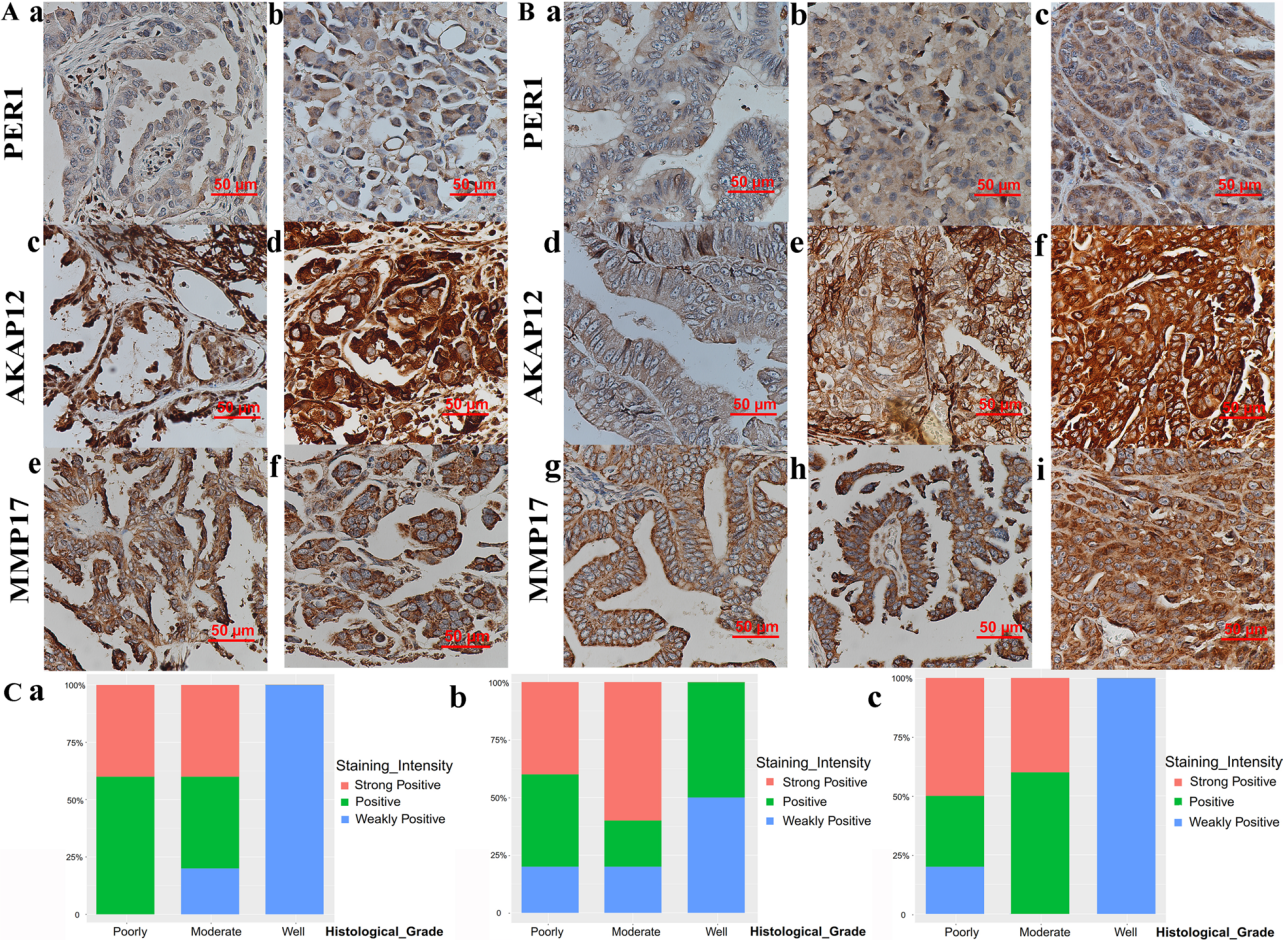
Immunohistochemical staining in paraffin-embedded human SOC. **A** PER1, AKAP12, and MMP17 IHC staining in SOC without (**a**, **c**, **e**) and with (**b**, **d**, **f**). metastasis. **B** PER1, AKAP12, and MMP17 IHC staining in well differentiation (**a**, **d**, **g**), moderate differentiation (**b**, **e**, **h**) and poor differentiation (**c**, **f**, **i**) SOC. **C** Histograms showed (**a**) PER1 (**b**) AKAP12 (**c**) MMP17 staining intensity in SOC with well differentiation, moderate differentiation and poor differentiation. And the weakly positive represents negative and light yellow staining, positive represents yellow staining, and strongly positive stands for brown staining

To explore the protein expression of PER1, AKAP12 and MMP17 in SOC, IHC staining was performed. As shown in Fig. 8A, B, the expression of PER1, AKAP12 and MMP17 was located in the cytoplasm of tumor cells. In SOC with metastasis, the staining intensity of PER1, AKAP12 and MMP17 was stronger than that in SOC without metastasis (Fig. 8A). And the difference of PER1, AKAP12 and MMP17 was statistically significant and correlation analysis showed that metastasis was positively correlated with PER1, AKAP12 and MMP17 expression (Tables 1 and 2). In TCGA and GEO cohorts, the advanced FIGO stages implied high risk score (Figure S9) and FIGO stages were classified according to metastasis.  PGCCs, a type of cancer stem cells with more invasion and metastasis ability, obtained higher risk scores than the control group (Fig. 7K), albeit the varied expression of PER1, AKAP12 and MMP17 in HEY and SKOv3 cell lines (Fig. 7I, J). However, the differences between the expression and other parameters including age and tumor size were not statistically significant (Additional files 13, 14). In addition, PER1, AKAP12 and MMP17 expression were associated with the malignant degree of SOC. SOCs with poor differentiation had the strongest staining intensity of PER1, AKAP12 and MMP17 and SOCs with well differentiation had the weakest staining intensity (Fig. 8B, C).

**Table 1:** Staining index of PER1, AKAP12 and MMP17 among SOC samples

	Staining index		Mann–Whitney U test	
	SOC without metastasis	SOC with metastasis	*Z* value	*P* Value
PER1	6.75 ± 3.77	8.94 ± 3.28	− 1.84	0.06
AKAP12	7.46 ± 3.99	10.00 ± 2.47	− 2.08	0.038
MMP17	6.58 ± 3.17	9.44 ± 3.28	− 2.32	0.02

**Table 2:** Correlations between PER1, AKAP12 and MMP17 expression and metastasis characteristics in SOC patients

	Staining index		Correlation analysis	
	SOC without metastasis	SOC with metastasis	Pearson coefficient	*P* value
PER1	6.75 ± 3.77	8.94 ± 3.28	0.298	0.056
AKAP12	7.46 ± 3.99	10.00 ± 2.47	0.352	0.022
MMP17	6.58 ± 3.17	9.44 ± 3.28	0.411	0.007

## Discussion

OC is the most lethal gynecological malignancy. The incidence of OC has globally increased from 1990 to 2019 [49]. The neighboring environment of the tumor, namely the TME, comprises both cellular and non-cellular components. The former includes immune cells and stromal cells, such as T/B lymphocytes, dendritic cells (DC), tumor-associated macrophages (TAM), myeloid-derived suppressor cells (MDSC), CAFs, and MSCs [50]. For example, functional T cells of different phenotype, quantity, and location distribute with different tumor immune cell phenotypes. The immune-excluded phenotype was characterized by less activated/exhausted CD8 + GZMB T cells and enrichment of predysfunctional CD8 + GZMK T cells and of resting CD4 + T cell populations. In contrast, activated CD4 + T cells and regulatory T cells were detected in the infiltrated tumor [51]. TAMs are a heterogeneous cell population and broadly classified into pro-inflammatory M1 and anti-inflammatory M2 macrophages. In OC, M2 macrophages comprise 39% of the immune cells and are associated with adverse clinical outcomes [52]. Single cell spatial analysis discloses intimate interactions of exhausted CD8^+^ T cells and PD-L1^+^ macrophages that are considered mechanistic determinants of response to niraparib and pembrolizumab treatment, which are PARP and immune checkpoint inhibitors, respectively [53]. Among stromal cells, CAFs originate from MSCs or by transdifferentiation of other cells. With the expression of specific molecules and receptors, CAFs promote angiogenesis, metastasis, and infiltration of immunosuppressive cells, thus fueling tumor growth and progression [54]. SOC stromal fibroblasts exhibit intrinsic resistance to PARPi and increased further after PARPi administration [55]. Except for the PARPi response, patients with high CAF infiltration exhibit chemoresistance and contribute to the insensitivity to immunotherapy [54, 56, 57]. Similarly, the increased dispersion of MSCs in SOCs tends to shorten survival and attenuates the response to immunotherapy of patients in our study.

In terms of the clinical relevance, the expression of MSC markers and the existence of soluble factors derived from MSCs are negatively correlated with the prognosis of patients. CD105 + MSCs were associated with a reduced OS of patients with brain neoplasm, lung cancer, and gastric cancer [58–60]. MMP9 and IL-6 are secreted proteins of MSCs. High expression of MMP9 has been associated with low survival rates in lung adenocarcinoma [61]. Patients with high IL-6 levels have significantly a poorer survival rate than those with low IL-6 levels [62]. The exosomal microRNAs released by MSCs were positively related to survival time in colorectal, myeloid leukemia, nasopharyngeal carcinoma, and glioma [63–66].

Essentially, MSCs exert immunomodulatory effects on both innate and adaptive cells through cell-to-cell contact and paracrine activity, including T cells, natural killer (NK) cells, and DCs. Induction of regulatory T cells (Tregs) is a main mechanism of immunosuppression by MSCs. MSCs can convert conventional T cells (T convs) to Forkhead box P3 (Foxp3) expressing Tregs [67]. Foxp3 is a transcription factor that inimitably defines Tregs and is a requirement for Tregs differentiation [68]. The immature dendritic cells are a subset of dendritic cells that selectively promote the proliferation of Tregs, and both take part in immunosuppressive activity [69]. Moreover, mature DCs co-cultured with MSCs skew to immature status and show a reduced stimulatory activity on T cells [70]. Therefore, in our study, patients with the mesenchymal phenotype tended to have an immunosuppressive state characterized by a richness of Tregs and immature DCs, and responded poorly to anti-PD-1 therapy. However, there are other immunoeffector cells that assembled in the high-risk score group. NK cells are innate cytotoxic lymphocytes and MSCs modulate their inhibitory effects on cell proliferation, altered cytotoxicity and cytokine production, and induction of apoptosis by MSC secreted cytokines such as prostaglandin E2 (PGE2), indoleamine 2,3-dioxygenase (IDO), TGF-β1, IL-6, and nitric oxide (NO) [71]. In our study, NK cells were enriched in the high MSC risk score group. This change largely resulted from the fact that there was heterogeneity among NK cells, and CD56^bright^ NK cells and CD56^dim^ NK cells are two main subsets of circulating human NK cells. CD56^bright^ NK cells are more immature and are more enriched in the tumor, and exhibit more limited cytotoxicity responses compared to CD56^dim^ NK cells [72], which is likely to occupy the majority of the mesenchymal phenotype of OC. In addition, Wan et al. revealed that the unique bispecific anti-programmed cell death protein 1 (PD-1)/programmed death-ligand 1 (PD-L1) antibody induced NK cells to transition from inert to more active and cytotoxic phenotypes, implicating NK cells as the key missing component of the current ICB-induced immune response in SOC [73].

With the exception of the prognostic response to ICB, our MSC score system is able to provide guidance for anti-angiogenesis therapy. As for patients with high-risk scores, standard chemotherapy plus angiogenic inhibitor is superior to chemotherapy alone. Stefani et al. found that low-dose irradiated MSCs showed antiangiogenic properties and infiltrated predominantly the perivascular niche, leading to rejection of established tumors [74]. MiR-16, a microRNA targeting VEGF, was enriched in MSC-derived exosomes and partially resulted in an antiangiogenic effect in breast cancer cells [75]. The MSC score system was a prospective marker for the administration of PARPi. The higher the risk scores, the lower the number of defects in homologous recombination, for which repair deficiency is closely associated with sensitivity to PARPi therapy in epithelial OC [76, 77]. Therefore, patients with a mesenchymal phenotype may not be suitable for treatment with PARPi.

In our validation experiment, three genes related to poor outcomes were prone to accumulate in the healthy ovary and in epithelial cells. However, the PER1 content is distinct between HEY and SKOv3 cells, which may be explained by the role of *TP53*. *TP53* in HEY cells is wild-type and in SKOv3 cells is deleted. PER1 knockdown influences pancreatic cancer cell lines with mutated *TP53*, but does not alter cells containing wild-type *TP53* [78], the reason for this finding is that p53 represses PER1 transcription [79]. MSCs can act directly on metastasis of tumor through production of pro-metastatic cytokines or regulation of epithelial-mesenchymal transition [80, 81]. Not only in SOC, the elevated proteins of PER1, AKAP12 and MMP17 are also associated with the migration and invasion in triple-negative breast cancer, melanoma and colon cancer [82–84].

Because of its multipotency, low immunogenicity, easy accessibility and ethical advantage compared to pluripotent stem cells or embryonic stem cells, MSCs are desirable candidates for in degenerative and inflammatory diseases, auto-immune diseases, such as joint injury, atopic dermatitis, and multiple sclerosis [85]. Infrapatellar fat pad-derived mesenchymal stem cells, proximal to the knee joint and similar to adipose cells, own proliferation and differentiation potential independent of age and promote hyaline-like cartilage formation without integration into the surrounding cartilage [86]. Currently, there are several phase I/II and III clinical trials involving immunomodulatory MSCs aimed at treating graft-versus-host disease and tumors. In combination with ganciclovir, genetically-modified autologous MSC were found to be safe and tolerable in patients with advanced gastrointestinal adenocarcinoma [87]. A similar trial confirmed that allogeneic MSC infusions showed safety and feasibility in patients with prostate cancer [88]. Another trial in which endovascular superselective intraarterial (ESIA) MSC infusions loaded with an oncolytic adenovirus Delta-24 (MSC-D24) were used to treat glioblastoma is currently underway [89]. The direction of treatment for MSCs mainly includes the delivery of various anticancer biological agents or suicide genes using an extracellular vesicle derived from MSCs [90, 91]. However, we have to face the possibility about the latent pro-metastasis functions and the promotion of immune evasion if anticancer agents or suicide genes in MSCs cannot function. In addition, MSCs combined with drug nanoparticles were used to induce the death of cancer cells. The conjugation forms between MSCs and nanoparticles include MSCs loading nanoparticles, nanoparticles attached to MSCs surface, nanoparticles coated with MSCs membrane, and vectors of anti-tumor genes in MSCs [92]. Because of tumor tropism of MSCs, the conjugation between MSCs and nanoparticles solved the problem of low target specificity and minimized side effects of conventional medicine. However, the toxicity of nanoparticles and the uncertainty of pharmacokinetics are still existent, including accumulation in organs followed by inflammation or binding with blood constituent followed by coagulation [93]. If these disadvantages about the safety of MSCs, nanoparticles or drugs can be solved, MSC may gradually be applied in clinical practice.

## Conclusions

Using a comprehensive transcriptomic analysis of genes characterizing MSCs, this study constructed an MSC-related prognostic model that could separate patients into two groups. The high-risk group was associated with a worse prognosis, a different immunosuppressive phenotype, and a weak response to anti-PD-1 treatment. There was also instructive significance of this sorting system for anti-angiogenesis therapy.

## Supplementary Information

**Additional file 1**. MSC-related gene signature. The gene signatures were extracted from the GOBP_MESENCHYMAL_STEM_CELL_DIFFERENTIATION and GOBP_MESENCHYMAL_STEM_CELL_PROLIFERATION in Molecular Signatures Database.

**Additional file 2**. Angiogenesis-related gene signature. The gene signatures were intersection of published papers investigating angiogenesis in ovarian cancer and the HALLMARK_ANGIOGENESIS from Molecular Signatures Database.

**Additional file 3**. The immune gene signatures. The gene signatures involved in immunity from 5 literatures and their coefficients.

**Additional file 4**. The tumor mutational burden of GSE91061. The tumor mutation burden of GSE91061.

**Additional file 5**. The primer sequence. The forward and reverse primer sequence of PER1, AKAP12 and MMP17.

**Additional file 6**. Protocol for reverse transcription and real-time polymerase chain reaction. Details for reverse transcription and real-time polymerase chain reaction.

**Additional file 7**. Protocol for immune-histochemistry. Details for immune-histochemistry.

**Additional file 8**. Grouping of samples in TCGA according to MSC score. The normalized enrichment scores and group information of each sample in TCGA.

**Additional file 9**. GO enrichment analysis between low and high MSC score group. GO enrichment analysis of DEGs between groups with low and high MSC scores.

**Additional file 10**. KEGG pathway analysis between low and high MSC score group. KEGG pathway analysis of DEGs between groups with low and high MSC scores.

**Additional file 11**. Genes related to prognosis in brown module. Prognostic genes in module correlated with MSC score.

**Additional file 12**. Genes in the MSC score related prognostic model. Multivariate Cox regression analysis was conducted to identify MSC-score-related genes associated with OS and three genes (MMP17, AKAP12, andPER1) were used to construct the prognostic model.

**Additional file 13**. C-index of TCGA cohort, GEO cohorts and nomogram. Standard error, minimum value, maximum value and P value of C-index of TCGA cohort, GEO cohorts and nomogram.

**Additional file 14**. Difference between staining index of PER1, AKAP12 and MMP17 and age among SOC samples. Difference between staining index of PER1, AKAP12 and MMP17 and age among SOC samples via Mann-Whitney U test.

**Additional file 15**. Difference between staining index of PER1, AKAP12 and MMP17 and tumor size among SOC samples. Difference between staining index of PER1, AKAP12 and MMP17 and tumor size among SOC samples via Mann-Whitney U test.

**Additional file 16: Figure S1**. Estimation of the best cutoff value for the MSC scores determined by the X-tile software. The interface of estimation of the best cutoff value for the MSC scores in TCGA-OV cohort by the X-tile software. **Figure S2**. Identification of the MSC-score-related gene set. (**A**) Clustering dendrograms to reject outliers. (**B**) Scale-free network confirmation with connectivity value k. (**C**) Module dendrogram and (**D**) gene dendrogram before and after combination of modules with similar expression patterns. **Figure S3**. Gene expression heatmap, risk scores and OS time distribution Gene expression heatmap, risk scores and OS time distribution of TCGA and GEO cohorts, and ROC curve of GEO datasets. **Figure S4**. Immunity inhibition factors and genetic characteristics in different MSC risk group. The level of immunity inhibition factors and genetic characteristics in different MSC risk group, which *P* value was more than 0.05. **Figure S5**. Estimation of the best cutoff value for the GSE19061 risk scores determined by X-tile software. The interface of estimation of the best cutoff value for the GSE19061 risk scores determined by X-tile software. **Figure S6**. Kaplan-Meier analysis of other biomarkers of GSE91061. To evaluate the predictive power of other biomarkers, PD-L1 and PD-1 expression status, 14 kinds of tumor-infiltrating lymphocytes, mutational burden, and immune gene signatures were correlation with the survival. **Figure S7**. Correlation between the abundance score of biomarkers and response to immunotherapy. The scores of other biomarkers in different response groups of GSE19061 and the proportion of patients with response to anti-PD-1 immunotherapy in different biomarker level groups. **Figure S8**. Evaluation of the prognostic model in the antiangiogenesis dataset and construction of the nomogram. Evaluation of the prognostic model in the antiangiogenesis dataset and conformity between nomogram prediction and actual observation in terms of the 1-(**C**), 3-(**D**), and 5-(**E**)year survival rates. **Figure S9**. Comparison of risk score in different FIGO stage, which means the condition on metastasis of SOC. Comparison of risk score in different FIGO stage, which means the condition on metastasis of SOC, in TCGA cohort and GSE14764 and GSE53963.

## Data Availability

The datasets used during the current study are available in the TCGA, GEO, UCAC Xena and TISIDB website. All data analysed during this study are included in this published article.

## Abbreviations

SOC: Serous ovarian carcinoma
PARPi: Poly ADP-ribose polymerase inhibitor
PFS: Progression-free survival
OS: Overall survival
TME: Tumor microenvironment
MSC: Mesenchymal stem cell
CAF: Carcinoma-associated fibroblast
CA-MSC: Cancer-associated mesenchymal stromal cell
PD-L1: Programmed death ligand 1
PD-1: Programmed death 1
ICB: Immune checkpoint blockade
HRD: Homologous defect recombination
FPKM: Fragments per kilobase million
TCGA: The Cancer Genome Atlas
GEO: Gene Expression Omnibus
GTEx: Genotype-tissue expression
TPM: Transcripts per million
MSigDB: Molecular signatures database
ssGSEA: Single sample gene set enrichment analysis
DEG: Differentially expressed gene
FDR: False discover rate
GO: Gene Ontology
KEGG: Kyoto Encyclopedia of Genes and Genomes
WGCNA: Weighted correlation network analysis
MMP17: Matrix metallopeptidase 17
AKAP12: A-kinase anchoring protein 12
PER1: Period circadian regulator 1
ITH: Intratumor heterogeneity
CNV: Copy number variation
TMB: Tumor mutation burden
DCA: Decision curve analysis
PGCC: Polyploid giant cancer cell
IHC: Immunohistochemistry
BP: Biological process
CC: Cellular component
MF: Molecular function
DC: Dendritic cell
TAM: Tumor associated macrophage
MDSC: Myeloid-derived suppressor cell
NK: Natural killer
Tregs: Regulatory T cells
PGE2: Prostaglandin E2
IDO: Indoleamine 2,3-dioxygenase
NO: Nitric oxide
