# Supplementary material for: Prognostic risk factors of serous ovarian carcinoma based on mesenchymal stem cell phenotype and guidance for therapeutic efficacy
Source: J Transl Med. 2023 Jul 11;21:456. doi: 10.1186/s12967-023-04284-3 (PMC10334653; doi:10.1186/s12967-023-04284-3)
Supplement: Supplementary file 1 — Additional file 1. MSC-related gene signature. The gene signatures were extracted from the GOBP_MESENCHYMAL_STEM_CELL_DIFFERENTIATION and GOBP_MESENCHYMAL_STEM_CELL_PROLIFERATION in Molecular Signatures Database. [file 12967_2023_4284_MOESM1_ESM.docx]

**Additional file 1** MSC-related gene signature

| **Metagene** | **Cell type** |
| --- | --- |
| CTNNB1 | MESENCHYMAL_STEM_CELL |
| GSK3B | MESENCHYMAL_STEM_CELL |
| MIR346 | MESENCHYMAL_STEM_CELL |
| PDGFRA | MESENCHYMAL_STEM_CELL |
| SOX6 | MESENCHYMAL_STEM_CELL |
| REST | MESENCHYMAL_STEM_CELL |
| SOX5 | MESENCHYMAL_STEM_CELL |
| SOX9 | MESENCHYMAL_STEM_CELL |
| WNT3 | MESENCHYMAL_STEM_CELL |
| FZD1 | MESENCHYMAL_STEM_CELL |
| SLC4A11 | MESENCHYMAL_STEM_CELL |
| CCNE1 | MESENCHYMAL_STEM_CELL |
| CITED1 | MESENCHYMAL_STEM_CELL |
| FERMT2 | MESENCHYMAL_STEM_CELL |
| LTBP3 | MESENCHYMAL_STEM_CELL |
| MIR16-1 | MESENCHYMAL_STEM_CELL |
| MIR29B1 | MESENCHYMAL_STEM_CELL |
| SIX2 | MESENCHYMAL_STEM_CELL |
| VEGFC | MESENCHYMAL_STEM_CELL |
