# Supplementary material for: Prognostic risk factors of serous ovarian carcinoma based on mesenchymal stem cell phenotype and guidance for therapeutic efficacy
Source: J Transl Med. 2023 Jul 11;21:456. doi: 10.1186/s12967-023-04284-3 (PMC10334653; doi:10.1186/s12967-023-04284-3)
Supplement: Supplementary file 2 — Additional file 2. Angiogenesis-related gene signature. The gene signatures were intersection of published papers investigating angiogenesis in ovarian cancer and the HALLMARK_ANGIOGENESIS from Molecular Signatures Database. [file 12967_2023_4284_MOESM2_ESM.docx]

**Additional file 2** Angiogenesis-related gene signature

| **Metagene** | **Cell type** |  |
| --- | --- | --- |
| ANG | Angiogenesis |  |
| ANGPTL3 | Angiogenesis |  |
| ANGPTL4 | Angiogenesis |  |
| CNN2 | Angiogenesis |  |
| COL4A1 | Angiogenesis |  |
| COL4A2 | Angiogenesis |  |
| CXCL1 | Angiogenesis | |
| CXCL12 | Angiogenesis | |
| DCN | Angiogenesis | |
| DLK1 | Angiogenesis | |
| EDNRA | Angiogenesis | |
| EGF | Angiogenesis | |
| ENG | Angiogenesis | |
| EPHB2 | Angiogenesis | |
| EPHB4 | Angiogenesis | |
| FAP | Angiogenesis | |
| FBLN1 | Angiogenesis | |
| FGF2 | Angiogenesis | |
| IL1B | Angiogenesis | |
| IL6 | Angiogenesis | |
| IL8 | Angiogenesis | |
| NRP1 | Angiogenesis | |
| PDGFRA | Angiogenesis | |
| PDGFRB | Angiogenesis | |
| PLAU | Angiogenesis | |
| PLAUR | Angiogenesis | |
| SERPINE1 | Angiogenesis | |
| SPHK1 | Angiogenesis | |
| TGFB1 | Angiogenesis | |
| TGM2 | Angiogenesis | |
| VEGFA | Angiogenesis | |
