# Supplementary material for: Prognostic risk factors of serous ovarian carcinoma based on mesenchymal stem cell phenotype and guidance for therapeutic efficacy
Source: J Transl Med. 2023 Jul 11;21:456. doi: 10.1186/s12967-023-04284-3 (PMC10334653; doi:10.1186/s12967-023-04284-3)
Supplement: Supplementary file 3 — Additional file 3. The immune gene signatures. The gene signatures involved in immunity from 5 literatures and their coefficients. [file 12967_2023_4284_MOESM3_ESM.docx]

**Additional file 3** The immune gene signatures

| **Genes** | **coefficients** | **PMID** |
| --- | --- | --- |
| KLRK1 | -0.22375 | 34291650 |
| KIR2DL4 | -0.03967 |  |
| IL18RAP | -0.01885 |  |
| KIR3DL1 | -0.28068 |  |
| IL27 | -0.32146 |  |
| CYTL1 | 0.007 | 33428606 |
| CCL8 | -0.033 |  |
| FCGR2C | -0.021 |  |
| OAS1 | -0.015 |  |
| HAPLN3 | -0.037 |  |
| WIPF1 | -0.014 |  |
| CLIC2 | -0.023 |  |
| BATF2 | −0.147 | 32731180 |
| CTLA4 | −0.135 |  |
| EGFR | 0.283 |  |
| IKBKG | −0.560 |  |
| PIK3R2 | 0.773 |  |
| PPP3CA | −0.399 |  |
| SEL1L3 | -0.06359 | 7985195 |
| BST2 | -0.023 |  |
| IFITM1 | -0.00263 |  |
| PSME1 | -0.30396 | 32310824 |
| CDC42 | -0.24399 |  |
| CMTM6 | -0.23548 |  |
| HLA-DQB1 | -0.07312 |  |
| HLA-C | -0.10692 |  |
| CXCR6 | -0.03143 |  |
| CD8B | -0.05033 |  |
| TNFSF13 | -0.25872 |  |
