# Supplementary material for: Prognostic risk factors of serous ovarian carcinoma based on mesenchymal stem cell phenotype and guidance for therapeutic efficacy
Source: J Transl Med. 2023 Jul 11;21:456. doi: 10.1186/s12967-023-04284-3 (PMC10334653; doi:10.1186/s12967-023-04284-3)
Supplement: Supplementary file 4 — Additional file 4. The tumor mutational burden of GSE91061. The tumor mutation burden of GSE91061. [file 12967_2023_4284_MOESM4_ESM.docx]

**Additional file 4** The tumor mutational burden of GSE91061

| **ID** | **total_perMB** | **ID** | **total_perMB** |
| --- | --- | --- | --- |
| Pt10_Pre_E9047565-6 | 1.4 | Pt47_Pre_AD506073-6 | 17.88 |
| Pt101_Pre_AD486328-5 | 0.18 | Pt48_Pre_E9047561-7 | 1.72 |
| Pt103_Pre_AE134058-2 | 0.38 | Pt49_Pre_AD667851-6 | 15.24 |
| Pt106_Pre_AD502250-5 | 12.26 | Pt5_Pre_E9021022-6 | 0.92 |
| Pt11_Pre_AD153352-6 | 1.9 | Pt52_Pre_AD506075-6 | 6.34 |
| Pt17_Pre_E9047563-6 | 0.32 | Pt59_Pre_AD823915-5 | 7.54 |
| Pt18_Pre_E9024732-6 | 4.06 | Pt65_Pre_AD793919-6 | 45.78 |
| Pt23_Pre_AD313075-5 | 11.6 | Pt66_Pre_AD667850-6 | 2.52 |
| Pt24_Pre_AD436687-5 | 0.02 | Pt67_Pre_AD506074-6 | 0.28 |
| Pt26_Pre_AD467789-6 | 2.24 | Pt72_Pre_AD793922-5 | 7.74 |
| Pt27_Pre_AD453873-5 | 3.32 | Pt76_Pre_AD667852-6 | 4 |
| Pt28_Pre_AD297619-6 | 0.72 | Pt77_Pre_AD733591-7 | 0.84 |
| Pt29_Pre_AD497504-5 | 7.26 | Pt79_Pre_AD733587-5 | 9.86 |
| Pt3_Pre_E9024733-3 | 3.16 | Pt8_Pre_AD153354-6 | 1.6 |
| Pt30_Pre_AD497503-5 | 1.12 | Pt82_Pre_AD823914-8 | 0.62 |
| Pt31_Pre_AD453872-5 | 7.92 | Pt84_Pre_AD486532-5 | 0.18 |
| Pt34_Pre_AD466985-6 | 3.82 | Pt85_Pre_AD486329-5 | 2.38 |
| Pt36_Pre_AD467095-6 | 0.04 | Pt89_Pre_AE070951-5 | 3.96 |
| Pt37_Pre_AD502452-5 | 0.5 | Pt9_Pre_E9021024-6 | 7.36 |
| Pt38_Pre_E9200719-6 | 7.9 | Pt90_Pre_AD467873-6 | 4.56 |
| Pt4_Pre_E9021023-6 | 5.04 | Pt92_Pre_AE134060-5 | 11.42 |
| Pt44_Pre_AD467790-6 | 6.52 | Pt94_Pre_AD732850-6 | 7.16 |
| Pt46_Pre_AD467096-6 | 3.3 | Pt98_Pre_AD733586-8 | 0.38 |
