# Supplementary material for: Prognostic risk factors of serous ovarian carcinoma based on mesenchymal stem cell phenotype and guidance for therapeutic efficacy
Source: J Transl Med. 2023 Jul 11;21:456. doi: 10.1186/s12967-023-04284-3 (PMC10334653; doi:10.1186/s12967-023-04284-3)
Supplement: Supplementary file 5 — Additional file 5. The primer sequence. The forward and reverse primer sequence of PER1, AKAP12 and MMP17. [file 12967_2023_4284_MOESM5_ESM.docx]

**Additional file 5** The primer sequence

| **Primer** | **Sequence** |
| --- | --- |
| F(PER1) | GTCACCTCCCACCTTTGG |
| R(PER1) | GCTGTCTCCCCGCAATAA |
| F(MMP17) | CACTCATGTACTACGCCCTCA |
| R(MMP17) | TGGAGAAGTCGATCTGGATGTC |
| F(AKAP12) | GAGATGGCTACTAAGTCAGCGG |
| R(AKAP12) | CAGTGGGTTGTGTTAGCTCTTC |
