# Supplementary material for: Prognostic risk factors of serous ovarian carcinoma based on mesenchymal stem cell phenotype and guidance for therapeutic efficacy
Source: J Transl Med. 2023 Jul 11;21:456. doi: 10.1186/s12967-023-04284-3 (PMC10334653; doi:10.1186/s12967-023-04284-3)
Supplement: Supplementary file 6 — Additional file 6. Protocol for reverse transcription and real-time polymerase chain reaction. Details for reverse transcription and real-time polymerase chain reaction. [file 12967_2023_4284_MOESM6_ESM.docx]

**Additional file 6** Protocol for reverse transcription and RT-PCR

| **Steps of first strand cDNA synthesis** | | | |
| --- | --- | --- | --- |
| 1. Removal of residual genomic DNA | | | |
| Prepare the following mixture in RNase free centrifuge tube, and gently blow the mixture with a pipette. Incubate at 42 ℃ for 2 min. | | | |
| Components | Usage | | |
| RNase-free H2O | To 15 μL | | |
| 5×gDNA digester Mix | 3 μL | | |
| Total RNA | 1μg | | |
| 2. Preparation of reverse transcription system | | | |
| Add 5μL 4×Hifair ®Ⅲ SuperMix directly into the step 1 tube, gently blow and mix with a pipette. | | | |
| 3. Reverse transcription program setting | | | |
| Temperature | Time | | |
| 25℃ | 5 min | | |
| 55℃ | 15 min | | |
| 85℃ | 5 min | | |
| The reverse transcripts can be used for qPCR reaction immediately or stored at - 20 ℃ for a short time. If long-term storage is required, it is recommended to store them at - 80 ℃ after subpackage to avoid repeated freezing and thawing. | | | |
| **Configuration of PCR reaction buffer** | | | |
| 1. Preparation of PCR Reaction System | | | |
| Components | Volume | Final concentration | |
| Hieff UNICON® Universal Blue qPCR SYBR Green Master Mix | 10μL | 1 | |
| Forward Primer (10 μM) | 0.4μL | 0.2 μM | |
| Reverse Primer (10 μM) | 0.4μL | 0.2 μM | |
| cDNA | 2μL | - | |
| DEPC water | To 20 | - | |
| 2. Operation PCR Reaction System | | | |
| Cycle Steps | Temperature | Time | Number of cycles |
| Pre-denaturation | 95℃ | 2 min | 1 |
| Denaturation | 95℃ | 10 sec | 40 |
| Annealing and extension | 60℃ | 30 sec |  |
| Melting curve stage | Default setting | | 1 |
