# Supplementary material for: Prognostic risk factors of serous ovarian carcinoma based on mesenchymal stem cell phenotype and guidance for therapeutic efficacy
Source: J Transl Med. 2023 Jul 11;21:456. doi: 10.1186/s12967-023-04284-3 (PMC10334653; doi:10.1186/s12967-023-04284-3)
Supplement: Supplementary file 7 — Additional file 7. Protocol for immune-histochemistry. Details for immune-histochemistry. [file 12967_2023_4284_MOESM7_ESM.docx]

**Additional file 7** Protocol for immunohistochemistry

| **Reagent** | **Time** | **Temperature** |
| --- | --- | --- |
| Deparaffinization with xylene | 15min*2 | Room temperature (RT) |
| Rehydration with ethyl alcohol, 95% ethanol, 90% ethanol, 80% ethanol, 70% ethanol and water | 5min | RT |
| Antigen retrieval with ethylenediaminetetraacetic acid (EDTA) buffer | 100℃ , 40℃ | 5min 15min |
| Cooling down to room temperature naturally, rinsing in phosphate‐buffered saline (PBS) for 5 min*3 |  |  |
| Blocking endogenous peroxidase with Reagent 1 | 15min | RT |
| Rinsing in PBS for 5 min*3, incubation with primary antibody | Overnight | 4℃ |
| Next day, rinsing in PBS for 5 min*3, incubation with Reagent 2 (Reaction enhancer) | 20min | 37℃ |
| Rinsing in PBS for 5 min*3, incubation with Reagent 3 (Enhanced enzyme-labeled goat anti-rabbit IgG polymer) | 20min | 37℃ |
| Rinsing in PBS for 5 min*3, 3,3′-diaminobenzidine was added for visualization and 10% Mayer’s hematoxylin was used as counter stain |  |  |
| Dehydrated, transparent and sealed with neutral balsam |  |  |
