# Supplementary material for: Prognostic risk factors of serous ovarian carcinoma based on mesenchymal stem cell phenotype and guidance for therapeutic efficacy
Source: J Transl Med. 2023 Jul 11;21:456. doi: 10.1186/s12967-023-04284-3 (PMC10334653; doi:10.1186/s12967-023-04284-3)
Supplement: Supplementary file 8 — Additional file 8. Grouping of samples in TCGA according to MSC score. The normalized enrichment scores and group information of each sample in TCGA. [file 12967_2023_4284_MOESM8_ESM.docx]

**Additional file 8** Grouping of samples in TCGA according to MSC score

| **ID** | **MSC-scores** | **MSC-scores groups** |
| --- | --- | --- |
| TCGA-61-1907-01A-01R-1567-13 | 0 | low |
| TCGA-10-0936-01A-01R-1564-13 | 0.21743 | low |
| TCGA-61-2092-01A-01R-1568-13 | 0.282142 | low |
| TCGA-61-2088-01A-01R-1568-13 | 0.30174 | low |
| TCGA-24-1846-01A-01R-1567-13 | 0.314976 | low |
| TCGA-61-2109-01A-01R-1568-13 | 0.324086 | low |
| TCGA-29-1781-01A-01R-1567-13 | 0.330268 | low |
| TCGA-09-0366-01A-01R-1564-13 | 0.346269 | low |
| TCGA-25-2392-01A-01R-1569-13 | 0.355695 | low |
| TCGA-23-1024-01A-02R-1564-13 | 0.361705 | low |
| TCGA-13-0885-01A-02R-1569-13 | 0.364406 | low |
| TCGA-29-1691-01A-01R-1566-13 | 0.369546 | low |
| TCGA-24-1565-01A-01R-1566-13 | 0.375503 | low |
| TCGA-61-1910-01A-01R-1567-13 | 0.376162 | low |
| TCGA-24-1419-01A-01R-1565-13 | 0.393469 | low |
| TCGA-04-1350-01A-01R-1565-13 | 0.394938 | low |
| TCGA-04-1365-01A-01R-1565-13 | 0.395687 | low |
| TCGA-29-1697-01A-01R-1567-13 | 0.399367 | low |
| TCGA-31-1950-01A-01R-1568-13 | 0.400661 | low |
| TCGA-13-0905-01B-01R-1565-13 | 0.402544 | low |
| TCGA-61-1995-01A-01R-1568-13 | 0.420016 | low |
| TCGA-20-0991-01A-01R-1564-13 | 0.424052 | low |
| TCGA-30-1860-01A-01R-1568-13 | 0.434993 | low |
| TCGA-23-1021-01B-01R-1564-13 | 0.438468 | low |
| TCGA-10-0931-01A-01R-1564-13 | 0.447454 | low |
| TCGA-13-0916-01A-01R-1564-13 | 0.448677 | low |
| TCGA-24-1845-01A-01R-1567-13 | 0.450953 | low |
| TCGA-23-1107-01A-01R-1564-13 | 0.451457 | low |
| TCGA-29-1696-01A-01R-1567-13 | 0.454198 | low |
| TCGA-24-1560-01A-01R-1566-13 | 0.456214 | low |
| TCGA-13-0725-01A-01R-1564-13 | 0.458101 | low |
| TCGA-13-0797-01A-01R-1564-13 | 0.465903 | low |
| TCGA-04-1361-01A-01R-1565-13 | 0.470559 | low |
| TCGA-24-1428-01A-01R-1564-13 | 0.470823 | low |
| TCGA-13-1492-01A-01R-1565-13 | 0.47264 | low |
| TCGA-13-0804-01A-01R-1564-13 | 0.472644 | low |
| TCGA-13-1409-01A-01R-1565-13 | 0.47426 | low |
| TCGA-29-1762-01A-01R-1567-13 | 0.478358 | low |
| TCGA-04-1648-01A-01R-1567-13 | 0.478454 | low |
| TCGA-29-1784-01A-02R-1567-13 | 0.479155 | low |
| TCGA-13-1489-01A-01R-1565-13 | 0.479633 | low |
| TCGA-61-1918-01A-01R-1568-13 | 0.484918 | low |
| TCGA-24-2281-01A-01R-1568-13 | 0.484956 | low |
| TCGA-29-2428-01A-01R-1569-13 | 0.487846 | low |
| TCGA-13-0765-01A-01R-1564-13 | 0.490191 | low |
| TCGA-61-1911-01A-01R-1567-13 | 0.492148 | low |
| TCGA-13-1407-01A-01R-1565-13 | 0.493447 | low |
| TCGA-13-1510-01A-02R-1565-13 | 0.494162 | low |
| TCGA-24-1551-01A-01R-1566-13 | 0.495589 | low |
| TCGA-09-2056-01B-01R-1568-13 | 0.496633 | low |
| TCGA-13-0720-01A-01R-1564-13 | 0.497622 | low |
| TCGA-24-0979-01A-01R-1565-13 | 0.498321 | low |
| TCGA-31-1956-01A-01R-1568-13 | 0.498543 | low |
| TCGA-23-1111-01A-01R-1567-13 | 0.498767 | low |
| TCGA-13-0795-01A-01R-1564-13 | 0.498778 | low |
| TCGA-20-0987-01A-02R-1564-13 | 0.499164 | low |
| TCGA-24-1470-01A-01R-1566-13 | 0.499681 | low |
| TCGA-24-0966-01A-01R-1564-13 | 0.502186 | low |
| TCGA-09-1670-01A-01R-1566-13 | 0.502571 | low |
| TCGA-24-1469-01A-01R-1566-13 | 0.504681 | low |
| TCGA-24-1431-01A-01R-1566-13 | 0.50714 | low |
| TCGA-24-1471-01A-01R-1566-13 | 0.512446 | low |
| TCGA-24-1557-01A-01R-1566-13 | 0.513468 | low |
| TCGA-09-1659-01B-01R-1564-13 | 0.513729 | low |
| TCGA-13-0766-01A-02R-1564-13 | 0.517573 | low |
| TCGA-25-1870-01A-01R-1567-13 | 0.519176 | low |
| TCGA-24-1842-01A-01R-1567-13 | 0.519994 | low |
| TCGA-24-1603-01A-01R-1566-13 | 0.520868 | low |
| TCGA-29-1688-01A-01R-1566-13 | 0.523602 | low |
| TCGA-59-2350-01A-01R-1569-13 | 0.526456 | low |
| TCGA-09-1665-01B-01R-1566-13 | 0.526473 | low |
| TCGA-29-1707-02A-01R-1567-13 | 0.52656 | low |
| TCGA-23-1123-01A-01R-1565-13 | 0.527111 | low |
| TCGA-04-1530-01A-02R-1569-13 | 0.528078 | low |
| TCGA-24-1553-01A-01R-1566-13 | 0.528624 | low |
| TCGA-13-0762-01A-01R-1564-13 | 0.529166 | low |
| TCGA-25-2391-01A-01R-1569-13 | 0.529279 | low |
| TCGA-09-0364-01A-02R-1564-13 | 0.531575 | low |
| TCGA-OY-A56Q-01A-11R-A406-31 | 0.53304 | low |
| TCGA-04-1651-01A-01R-1567-13 | 0.534162 | low |
| TCGA-25-2396-01A-01R-1569-13 | 0.535193 | low |
| TCGA-13-1488-01A-01R-1565-13 | 0.535388 | low |
| TCGA-23-1027-01A-02R-1564-13 | 0.537185 | low |
| TCGA-13-0923-01A-01R-1564-13 | 0.538314 | low |
| TCGA-61-1736-01B-01R-1568-13 | 0.540843 | low |
| TCGA-61-1738-01A-01R-1567-13 | 0.542008 | low |
| TCGA-09-2048-01A-01R-1568-13 | 0.542744 | low |
| TCGA-13-0891-01A-01R-1564-13 | 0.542974 | low |
| TCGA-10-0938-01A-02R-1564-13 | 0.543302 | low |
| TCGA-13-0911-01A-01R-1564-13 | 0.548632 | low |
| TCGA-29-2427-01A-01R-1569-13 | 0.548765 | low |
| TCGA-24-1413-01A-01R-1565-13 | 0.549814 | low |
| TCGA-61-2110-01A-01R-1568-13 | 0.552209 | low |
| TCGA-13-0724-01A-01R-1564-13 | 0.552642 | low |
| TCGA-29-1761-01A-01R-1567-13 | 0.554171 | low |
| TCGA-09-1666-01A-01R-1566-13 | 0.555652 | low |
| TCGA-24-2033-01A-01R-1568-13 | 0.556643 | low |
| TCGA-13-1496-01A-01R-1565-13 | 0.559254 | low |
| TCGA-29-1777-01A-01R-1567-13 | 0.562117 | low |
| TCGA-09-2044-01B-01R-1568-13 | 0.562789 | low |
| TCGA-04-1542-01A-01R-1566-13 | 0.563451 | low |
| TCGA-61-1725-01A-01R-1567-13 | 0.564986 | low |
| TCGA-61-2104-01A-01R-1568-13 | 0.565487 | low |
| TCGA-25-1319-01A-01R-1565-13 | 0.566179 | low |
| TCGA-29-1690-01A-01R-1566-13 | 0.566313 | low |
| TCGA-09-1662-01A-01R-1566-13 | 0.566685 | low |
| TCGA-24-2023-01A-01R-1567-13 | 0.56744 | low |
| TCGA-13-1498-01A-01R-1565-13 | 0.568744 | low |
| TCGA-13-0913-02A-01R-1564-13 | 0.569147 | low |
| TCGA-VG-A8LO-01A-11R-A406-31 | 0.569782 | low |
| TCGA-61-2002-01A-01R-1568-13 | 0.570642 | low |
| TCGA-31-1946-01A-01R-1568-13 | 0.573766 | low |
| TCGA-61-1724-01A-01R-1568-13 | 0.57441 | low |
| TCGA-23-1028-01A-01R-1564-13 | 0.575766 | low |
| TCGA-24-2026-01A-01R-1567-13 | 0.575841 | low |
| TCGA-61-1914-01A-01R-1567-13 | 0.576284 | low |
| TCGA-24-1924-01A-01R-1567-13 | 0.5766 | low |
| TCGA-23-1122-01A-01R-1565-13 | 0.576885 | low |
| TCGA-04-1343-01A-01R-1564-13 | 0.576901 | low |
| TCGA-09-1667-01C-01R-1566-13 | 0.579872 | low |
| TCGA-61-1741-01A-02R-1567-13 | 0.580043 | low |
| TCGA-13-1506-01A-01R-1565-13 | 0.580903 | low |
| TCGA-25-1312-01A-01R-1565-13 | 0.582322 | low |
| TCGA-13-1403-01A-01R-1565-13 | 0.58258 | low |
| TCGA-20-1682-01A-01R-1564-13 | 0.583788 | low |
| TCGA-29-1785-01A-01R-1567-13 | 0.584958 | low |
| TCGA-13-0727-01A-01R-1564-13 | 0.588471 | low |
| TCGA-25-1634-01A-01R-1566-13 | 0.588814 | low |
| TCGA-24-1417-01A-01R-1565-13 | 0.589692 | low |
| TCGA-25-1316-01A-01R-1565-13 | 0.592965 | low |
| TCGA-24-2036-01A-01R-1568-13 | 0.595028 | low |
| TCGA-24-2038-01A-01R-1568-13 | 0.600461 | low |
| TCGA-61-2000-01A-01R-1568-13 | 0.600593 | low |
| TCGA-09-1661-01B-01R-1566-13 | 0.602121 | low |
| TCGA-24-2297-01A-01R-1568-13 | 0.604257 | low |
| TCGA-61-1721-01A-01R-1569-13 | 0.606146 | low |
| TCGA-13-1483-01A-01R-1565-13 | 0.607975 | low |
| TCGA-WR-A838-01A-12R-A406-31 | 0.608464 | low |
| TCGA-13-0726-01A-01R-1564-13 | 0.609729 | low |
| TCGA-24-1923-01A-01R-1567-13 | 0.610116 | low |
| TCGA-09-1673-01A-01R-1566-13 | 0.611486 | low |
| TCGA-13-2060-01A-01R-1568-13 | 0.613297 | low |
| TCGA-24-2290-01A-01R-1568-13 | 0.613718 | low |
| TCGA-09-2051-01A-01R-1568-13 | 0.614224 | low |
| TCGA-59-2363-01A-01R-1569-13 | 0.61583 | low |
| TCGA-29-1774-01A-01R-1567-13 | 0.616847 | low |
| TCGA-04-1519-01A-01R-1565-13 | 0.617383 | low |
| TCGA-13-1404-01A-01R-1565-13 | 0.618129 | low |
| TCGA-24-1464-01A-01R-1566-13 | 0.618568 | low |
| TCGA-59-2351-01A-01R-1569-13 | 0.619888 | low |
| TCGA-04-1356-01A-01R-1569-13 | 0.621392 | low |
| TCGA-04-1347-01A-01R-1564-13 | 0.623669 | low |
| TCGA-29-1778-01A-01R-1567-13 | 0.62591 | low |
| TCGA-25-2409-01A-01R-1569-13 | 0.627359 | low |
| TCGA-24-1467-01A-01R-1566-13 | 0.627676 | low |
| TCGA-36-1577-01A-01R-1566-13 | 0.628081 | low |
| TCGA-09-1668-01B-01R-1566-13 | 0.628418 | low |
| TCGA-23-1026-01B-01R-1569-13 | 0.628747 | low |
| TCGA-59-2348-01A-01R-1569-13 | 0.630474 | low |
| TCGA-59-2355-01A-01R-1569-13 | 0.631903 | low |
| TCGA-23-2077-01A-01R-1568-13 | 0.632731 | low |
| TCGA-23-1022-01A-01R-1564-13 | 0.634444 | low |
| TCGA-57-1582-01A-01R-1566-13 | 0.636285 | low |
| TCGA-24-1549-01A-01R-1566-13 | 0.637202 | low |
| TCGA-23-1120-01A-02R-1565-13 | 0.637619 | low |
| TCGA-10-0927-01A-02R-1564-13 | 0.638061 | low |
| TCGA-13-0887-01A-01R-1564-13 | 0.638655 | low |
| TCGA-31-1944-01A-01R-1568-13 | 0.639222 | low |
| TCGA-23-1113-01A-01R-1564-13 | 0.639304 | low |
| TCGA-30-1862-01A-02R-1568-13 | 0.639479 | low |
| TCGA-24-1105-01A-01R-1565-13 | 0.639506 | low |
| TCGA-24-1844-01A-01R-1567-13 | 0.639627 | low |
| TCGA-24-1474-01A-01R-1566-13 | 0.639733 | low |
| TCGA-23-1110-01A-01R-1564-13 | 0.64045 | low |
| TCGA-25-2400-01A-01R-1569-13 | 0.643767 | low |
| TCGA-29-1703-01A-01R-1567-13 | 0.645001 | low |
| TCGA-25-2399-01A-01R-1569-13 | 0.647044 | low |
| TCGA-23-2084-01A-02R-1568-13 | 0.648995 | low |
| TCGA-23-1023-01A-02R-1564-13 | 0.649192 | low |
| TCGA-23-1119-01A-02R-1565-13 | 0.65016 | low |
| TCGA-29-2425-01A-01R-1569-13 | 0.650721 | low |
| TCGA-24-1416-01A-01R-1565-13 | 0.650771 | low |
| TCGA-29-1769-01A-01R-1567-13 | 0.651181 | low |
| TCGA-13-0884-01B-01R-1565-13 | 0.651292 | low |
| TCGA-23-1118-01A-01R-1564-13 | 0.652597 | low |
| TCGA-13-1511-01A-01R-1565-13 | 0.653711 | low |
| TCGA-24-2261-01A-01R-1568-13 | 0.654228 | low |
| TCGA-57-1586-01A-02R-1567-13 | 0.656148 | low |
| TCGA-24-1847-01A-01R-1566-13 | 0.656251 | low |
| TCGA-36-1570-01A-01R-1566-13 | 0.656251 | low |
| TCGA-61-2111-01A-01R-1568-13 | 0.656476 | low |
| TCGA-04-1364-01A-01R-1565-13 | 0.656554 | low |
| TCGA-09-1669-01A-01R-1566-13 | 0.65656 | low |
| TCGA-29-1694-01A-01R-1567-13 | 0.659253 | low |
| TCGA-09-0367-01A-01R-1564-13 | 0.659316 | low |
| TCGA-31-1953-01A-01R-1568-13 | 0.659547 | low |
| TCGA-24-1544-01A-01R-1566-13 | 0.662333 | low |
| TCGA-57-1583-01A-01R-1566-13 | 0.662373 | low |
| TCGA-36-1574-01A-01R-1566-13 | 0.662729 | low |
| TCGA-36-1568-01A-01R-1566-13 | 0.663064 | low |
| TCGA-24-1426-01A-01R-1565-13 | 0.664633 | low |
| TCGA-13-0901-01B-01R-1565-13 | 0.665289 | low |
| TCGA-24-1843-01A-01R-1567-13 | 0.665798 | low |
| TCGA-30-1853-01A-02R-1567-13 | 0.6664 | low |
| TCGA-57-1584-01A-01R-1566-13 | 0.666626 | low |
| TCGA-25-1630-01A-01R-1566-13 | 0.667715 | low |
| TCGA-24-2262-01A-01R-1568-13 | 0.668495 | low |
| TCGA-24-0968-01A-01R-1569-13 | 0.669367 | low |
| TCGA-24-1558-01A-01R-1566-13 | 0.670089 | low |
| TCGA-13-1505-01A-01R-1565-13 | 0.67026 | low |
| TCGA-29-1766-01A-01R-1567-13 | 0.670263 | low |
| TCGA-20-1687-01A-01R-1566-13 | 0.671332 | low |
| TCGA-04-1536-01A-01R-1566-13 | 0.673513 | low |
| TCGA-24-1567-01A-01R-1566-13 | 0.674106 | low |
| TCGA-13-0893-01B-01R-1565-13 | 0.675016 | low |
| TCGA-23-1029-01B-01R-1567-13 | 0.675454 | low |
| TCGA-25-1631-01A-01R-1569-13 | 0.676447 | low |
| TCGA-04-1357-01A-01R-1565-13 | 0.677278 | low |
| TCGA-04-1362-01A-01R-1565-13 | 0.677521 | low |
| TCGA-29-1711-01A-01R-1567-13 | 0.677581 | low |
| TCGA-24-1562-01A-01R-1566-13 | 0.678258 | low |
| TCGA-29-1701-01A-01R-1567-13 | 0.679642 | low |
| TCGA-24-2267-01A-01R-1568-13 | 0.679745 | low |
| TCGA-13-1507-01A-01R-1565-13 | 0.679882 | low |
| TCGA-29-1783-01A-01R-1567-13 | 0.680089 | low |
| TCGA-24-1435-01A-01R-1566-13 | 0.680416 | low |
| TCGA-25-1315-01A-01R-1565-13 | 0.681611 | low |
| TCGA-61-1919-01A-01R-1568-13 | 0.682399 | low |
| TCGA-09-2053-01C-01R-1568-13 | 0.682471 | low |
| TCGA-57-1994-01A-01R-1568-13 | 0.682626 | low |
| TCGA-04-1341-01A-01R-1564-13 | 0.683939 | low |
| TCGA-61-2012-01A-01R-1568-13 | 0.68517 | low |
| TCGA-25-2404-01A-01R-1569-13 | 0.685335 | low |
| TCGA-24-2254-01A-01R-1568-13 | 0.685897 | low |
| TCGA-13-1499-01A-01R-1565-13 | 0.68611 | low |
| TCGA-24-1604-01A-01R-1566-13 | 0.686553 | low |
| TCGA-13-A5FT-01A-11R-A406-31 | 0.688646 | low |
| TCGA-13-1497-01A-01R-1565-13 | 0.688873 | low |
| TCGA-09-0369-01A-01R-1564-13 | 0.688991 | low |
| TCGA-04-1332-01A-01R-1564-13 | 0.689126 | low |
| TCGA-61-1728-01A-01R-1568-13 | 0.689164 | low |
| TCGA-30-1861-01A-01R-1568-13 | 0.689218 | low |
| TCGA-25-1633-01A-01R-1566-13 | 0.689367 | low |
| TCGA-24-1616-01A-01R-1566-13 | 0.689543 | low |
| TCGA-04-1331-01A-01R-1569-13 | 0.689563 | low |
| TCGA-10-0937-01A-02R-1564-13 | 0.690319 | low |
| TCGA-13-1495-01A-01R-1565-13 | 0.690672 | low |
| TCGA-24-1104-01A-01R-1565-13 | 0.693562 | low |
| TCGA-13-0924-01A-01R-1564-13 | 0.69395 | low |
| TCGA-24-1103-01A-01R-1565-13 | 0.694763 | low |
| TCGA-36-1569-01A-01R-1566-13 | 0.69674 | low |
| TCGA-29-1776-01A-01R-1567-13 | 0.697111 | low |
| TCGA-13-0714-01A-01R-1564-13 | 0.699311 | low |
| TCGA-36-1581-01A-01R-1566-13 | 0.700822 | low |
| TCGA-10-0933-01A-01R-1569-13 | 0.702178 | low |
| TCGA-61-2009-01A-01R-1568-13 | 0.704929 | low |
| TCGA-13-0897-01A-01R-1564-13 | 0.705296 | low |
| TCGA-24-1430-01A-01R-1566-13 | 0.705589 | low |
| TCGA-25-2401-01A-01R-1569-13 | 0.706744 | low |
| TCGA-25-1313-01A-01R-1565-13 | 0.708325 | low |
| TCGA-23-1030-01A-02R-1564-13 | 0.709393 | low |
| TCGA-57-1993-01A-01R-1568-13 | 0.709688 | low |
| TCGA-24-1425-01A-02R-1566-13 | 0.713162 | low |
| TCGA-24-2271-01A-01R-1568-13 | 0.714142 | low |
| TCGA-30-1857-01A-02R-1569-13 | 0.715107 | low |
| TCGA-04-1655-01A-01R-1566-13 | 0.716235 | low |
| TCGA-25-1635-01A-01R-1566-13 | 0.717245 | low |
| TCGA-13-0900-01B-01R-1565-13 | 0.717505 | low |
| TCGA-29-2414-01A-02R-1569-13 | 0.718999 | low |
| TCGA-29-1693-01A-01R-1567-13 | 0.719188 | low |
| TCGA-25-2393-01A-01R-1569-13 | 0.722026 | low |
| TCGA-13-0886-01A-01R-1569-13 | 0.722098 | low |
| TCGA-13-1487-01A-01R-1565-13 | 0.722245 | low |
| TCGA-29-1705-01A-01R-1567-13 | 0.722497 | low |
| TCGA-24-2020-01A-01R-1567-13 | 0.722572 | low |
| TCGA-31-1951-01A-01R-1568-13 | 0.72303 | low |
| TCGA-5X-AA5U-01A-11R-A406-31 | 0.72343 | low |
| TCGA-61-2098-01A-01R-1568-13 | 0.724639 | low |
| TCGA-36-1571-01A-01R-1566-13 | 0.724647 | low |
| TCGA-25-1322-01A-01R-1565-13 | 0.72561 | low |
| TCGA-29-1710-01A-02R-1567-13 | 0.728355 | low |
| TCGA-13-0906-01A-01R-1564-13 | 0.72841 | low |
| TCGA-59-2352-01A-01R-1569-13 | 0.728878 | low |
| TCGA-24-2027-01A-01R-1567-13 | 0.731035 | low |
| TCGA-10-0928-01A-02R-1564-13 | 0.732 | low |
| TCGA-24-1552-01A-01R-1566-13 | 0.735404 | low |
| TCGA-13-0908-01B-01R-1565-13 | 0.735663 | low |
| TCGA-20-1686-01A-01R-1566-13 | 0.736334 | low |
| TCGA-23-1809-01A-01R-1566-13 | 0.736779 | low |
| TCGA-61-2008-01A-02R-1568-13 | 0.737085 | low |
| TCGA-25-1329-01A-01R-1565-13 | 0.73814 | low |
| TCGA-59-A5PD-01A-11R-A406-31 | 0.743505 | low |
| TCGA-24-1930-01A-01R-1567-13 | 0.744404 | low |
| TCGA-13-1509-01A-01R-1565-13 | 0.745427 | low |
| TCGA-09-2045-01A-01R-1568-13 | 0.7474 | low |
| TCGA-25-1627-01A-01R-1566-13 | 0.751853 | low |
| TCGA-24-2024-01A-02R-1568-13 | 0.752185 | low |
| TCGA-30-1891-01A-01R-1568-13 | 0.752879 | low |
| TCGA-24-1928-01A-01R-1567-13 | 0.75332 | low |
| TCGA-25-1321-01A-01R-1565-13 | 0.754592 | low |
| TCGA-29-1768-01A-01R-1567-13 | 0.755249 | low |
| TCGA-24-2298-01A-01R-1569-13 | 0.756217 | low |
| TCGA-13-1477-01A-01R-1565-13 | 0.7586 | low |
| TCGA-24-1546-01A-01R-1566-13 | 0.75877 | low |
| TCGA-23-1109-01A-01R-1564-13 | 0.758907 | low |
| TCGA-24-2280-01A-01R-1568-13 | 0.759433 | low |
| TCGA-24-1427-01A-01R-1565-13 | 0.760519 | low |
| TCGA-29-1763-01A-02R-1567-13 | 0.764838 | low |
| TCGA-31-1959-01A-01R-1568-13 | 0.766153 | low |
| TCGA-59-2354-01A-01R-1569-13 | 0.768452 | low |
| TCGA-25-1317-01A-01R-1565-13 | 0.770001 | low |
| TCGA-61-1737-01A-01R-1567-13 | 0.772238 | low |
| TCGA-36-1580-01A-01R-1566-13 | 0.777388 | low |
| TCGA-25-1320-01A-01R-1565-13 | 0.781815 | low |
| TCGA-24-1850-01A-01R-1567-13 | 0.781835 | low |
| TCGA-13-0883-01A-02R-1569-13 | 0.782407 | low |
| TCGA-24-2289-01A-01R-1568-13 | 0.783234 | low |
| TCGA-13-0920-01A-01R-1564-13 | 0.784682 | low |
| TCGA-25-1318-01A-01R-1565-13 | 0.785353 | low |
| TCGA-13-0800-01A-01R-1564-13 | 0.789911 | low |
| TCGA-24-2288-01A-01R-1568-13 | 0.789999 | low |
| TCGA-13-1410-01A-01R-1565-13 | 0.790762 | low |
| TCGA-57-1585-01A-01R-1566-13 | 0.79133 | low |
| TCGA-30-1718-01A-01R-1567-13 | 0.791388 | low |
| TCGA-29-1770-01A-01R-1567-13 | 0.792673 | low |
| TCGA-61-2097-01A-02R-1568-13 | 0.794582 | low |
| TCGA-24-1563-01A-01R-1566-13 | 0.794583 | low |
| TCGA-29-A5NZ-01A-11R-A406-31 | 0.794789 | high |
| TCGA-13-1485-01A-02R-1565-13 | 0.799465 | high |
| TCGA-04-1514-01A-01R-1566-13 | 0.799803 | high |
| TCGA-13-1405-01A-01R-1565-13 | 0.800121 | high |
| TCGA-23-1116-01A-01R-1564-13 | 0.804377 | high |
| TCGA-13-1501-01A-01R-1565-13 | 0.804702 | high |
| TCGA-61-2113-01A-01R-1568-13 | 0.805185 | high |
| TCGA-25-1632-01A-01R-1566-13 | 0.805819 | high |
| TCGA-25-1877-01A-01R-1567-13 | 0.806239 | high |
| TCGA-24-1422-01A-01R-1565-13 | 0.808701 | high |
| TCGA-61-1998-01A-01R-1568-13 | 0.809277 | high |
| TCGA-24-2035-01A-01R-1568-13 | 0.811637 | high |
| TCGA-24-1424-01A-01R-1565-13 | 0.812964 | high |
| TCGA-25-1623-01A-01R-1566-13 | 0.814632 | high |
| TCGA-24-0982-01A-01R-1565-13 | 0.818692 | high |
| TCGA-13-1512-01A-01R-1565-13 | 0.819551 | high |
| TCGA-24-2293-01A-01R-1568-13 | 0.820225 | high |
| TCGA-25-1328-01A-01R-1565-13 | 0.822686 | high |
| TCGA-25-1323-01A-01R-1565-13 | 0.828832 | high |
| TCGA-61-2003-01A-01R-1568-13 | 0.832323 | high |
| TCGA-30-1866-01A-02R-1568-13 | 0.832947 | high |
| TCGA-25-1628-01A-01R-1566-13 | 0.842503 | high |
| TCGA-29-1695-01A-01R-1567-13 | 0.843505 | high |
| TCGA-04-1338-01A-01R-1564-13 | 0.843999 | high |
| TCGA-20-1683-01A-01R-1566-13 | 0.84593 | high |
| TCGA-61-1733-01A-01R-1567-13 | 0.851561 | high |
| TCGA-13-0768-01A-01R-1569-13 | 0.855025 | high |
| TCGA-13-0888-01A-01R-1564-13 | 0.855698 | high |
| TCGA-25-1626-01A-01R-1566-13 | 0.856667 | high |
| TCGA-23-2078-01A-01R-1568-13 | 0.859244 | high |
| TCGA-24-1423-01A-01R-1565-13 | 0.861966 | high |
| TCGA-23-1114-01B-01R-1566-13 | 0.867974 | high |
| TCGA-30-1714-01A-02R-1567-13 | 0.872654 | high |
| TCGA-61-2101-01A-01R-1568-13 | 0.875414 | high |
| TCGA-24-1550-01A-01R-1566-13 | 0.880256 | high |
| TCGA-36-1576-01A-01R-1566-13 | 0.882244 | high |
| TCGA-13-1411-01A-01R-1565-13 | 0.883252 | high |
| TCGA-25-1326-01A-01R-1565-13 | 0.884254 | high |
| TCGA-61-1900-01A-01R-1567-13 | 0.889909 | high |
| TCGA-24-1418-01A-01R-1565-13 | 0.894379 | high |
| TCGA-13-1408-01A-01R-1565-13 | 0.900966 | high |
| TCGA-25-2398-01A-01R-1569-13 | 0.908399 | high |
| TCGA-30-1892-01A-01R-1568-13 | 0.910294 | high |
| TCGA-24-1434-01A-01R-1566-13 | 0.910878 | high |
| TCGA-09-2054-01A-01R-1568-13 | 0.915917 | high |
| TCGA-24-0970-01B-01R-1565-13 | 0.936494 | high |
| TCGA-61-2102-01A-01R-1568-13 | 0.963569 | high |
| TCGA-25-2042-01A-01R-1568-13 | 0.968364 | high |
| TCGA-13-0730-01A-01R-1564-13 | 1 | high |
