# Supplementary material for: Prognostic risk factors of serous ovarian carcinoma based on mesenchymal stem cell phenotype and guidance for therapeutic efficacy
Source: J Transl Med. 2023 Jul 11;21:456. doi: 10.1186/s12967-023-04284-3 (PMC10334653; doi:10.1186/s12967-023-04284-3)
Supplement: Supplementary file 9 — Additional file 9. GO enrichment analysis between low and high MSC score group. GO enrichment analysis of DEGs between groups with low and high MSC scores. [file 12967_2023_4284_MOESM9_ESM.docx]

**Additional file 9** GO enrichment analysis between low and high MSC score group

| **ONTOLOGY** | **ID** | **Description** | **pvalue** | **p.adjust** | **qvalue** | **geneID** |
| --- | --- | --- | --- | --- | --- | --- |
| BP | GO:0030198 | extracellular matrix organization | 4.24E-26 | 6.10E-23 | 4.81E-23 | 81794/81792/9509/9507/11173/56999/165/84168/151887/1215/1307/1277/1278/1281/1289/1290/1295/90993/83716/1511/11117/2199/2620/3036/3912/4015/4017/4035/4312/4323/4327/4313/4314/4811/22795/25903/5156/90102/64093 |
| BP | GO:0043062 | extracellular structure organization | 4.81E-26 | 6.10E-23 | 4.81E-23 | 81794/81792/9509/9507/11173/56999/165/84168/151887/1215/1307/1277/1278/1281/1289/1290/1295/90993/83716/1511/11117/2199/2620/3036/3912/4015/4017/4035/4312/4323/4327/4313/4314/4811/22795/25903/5156/90102/64093 |
| BP | GO:0045229 | external encapsulating structure organization | 6.18E-26 | 6.10E-23 | 4.81E-23 | 81794/81792/9509/9507/11173/56999/165/84168/151887/1215/1307/1277/1278/1281/1289/1290/1295/90993/83716/1511/11117/2199/2620/3036/3912/4015/4017/4035/4312/4323/4327/4313/4314/4811/22795/25903/5156/90102/64093 |
| BP | GO:0001503 | ossification | 1.90E-13 | 1.41E-10 | 1.11E-10 | 89/54829/1009/7123/1277/1278/1290/1291/90993/8642/2737/9464/3082/4015/4256/4323/4313/4330/4653/4656/5744/202151/6474/10736/64093/6591/30812/6678/7043/1462 |
| BP | GO:0031589 | cell-substrate adhesion | 1.53E-11 | 9.06E-09 | 7.14E-09 | 89/81792/56999/84168/151887/1307/1277/136227/1281/1295/11117/2199/2335/3678/8516/3912/4035/10894/4323/4653/4811/22795/90102/5054/7057/23671 |
| BP | GO:0035987 | endodermal cell differentiation | 6.04E-10 | 2.66E-07 | 2.09E-07 | 1289/1290/1291/1295/2335/3624/3678/3912/4323/4313 |
| BP | GO:0061448 | connective tissue development | 6.28E-10 | 2.66E-07 | 2.09E-07 | 59/81792/11173/633/1277/1289/64641/2737/9464/3207/4015/4017/4256/579/5159/5744/6474/10736/6591/30812 |
| BP | GO:0060485 | mesenchyme development | 1.31E-09 | 4.46E-07 | 3.51E-07 | 58/59/70/72/187/1277/147906/8642/1909/2335/9464/3082/4017/5159/90102/202151/10736/6591/30812/7043/51384 |
| BP | GO:0007369 | gastrulation | 1.35E-09 | 4.46E-07 | 3.51E-07 | 187/55997/1289/1290/1291/1295/2335/3207/3624/3678/8516/3912/4323/4313/8013/90102/10736 |
| BP | GO:0032963 | collagen metabolic process | 2.91E-09 | 8.61E-07 | 6.78E-07 | 9509/1277/1278/1289/90993/11117/4312/4323/4327/4313/4314/5159/7043 |
| BP | GO:0001706 | endoderm formation | 4.05E-09 | 1.00E-06 | 7.88E-07 | 1289/1290/1291/1295/2335/3624/3678/3912/4323/4313 |
| BP | GO:0032330 | regulation of chondrocyte differentiation | 4.05E-09 | 1.00E-06 | 7.88E-07 | 81792/11173/2737/3207/4017/579/5744/6474/10736/6591 |
| BP | GO:0030199 | collagen fibril organization | 1.40E-08 | 3.19E-06 | 2.51E-06 | 9509/165/1277/1278/1281/1289/1290/11117/4015/4017 |
| BP | GO:0001704 | formation of primary germ layer | 1.88E-08 | 3.98E-06 | 3.13E-06 | 1289/1290/1291/1295/2335/3207/3624/3678/8516/3912/4323/4313/10736 |
| BP | GO:0003012 | muscle system process | 3.60E-08 | 7.11E-06 | 5.60E-06 | 58/59/70/89/775/8913/1264/1674/1909/10052/2852/9464/23704/23630/10324/4626/4632/4653/4656/8013/221476/5592/5997/5999 |
| BP | GO:0060348 | bone development | 4.30E-08 | 7.59E-06 | 5.99E-06 | 89/633/1277/56603/8642/2200/2737/8111/3207/4015/4323/4653/202151/6424/6474/7043 |
| BP | GO:0007160 | cell-matrix adhesion | 4.36E-08 | 7.59E-06 | 5.99E-06 | 89/81792/56999/1307/1281/11117/2335/8516/4035/10894/4323/4653/4811/22795/90102/5054/7057 |
| BP | GO:0061035 | regulation of cartilage development | 8.29E-08 | 1.36E-05 | 1.08E-05 | 81792/11173/2737/3207/4017/579/5744/6474/10736/6591 |
| BP | GO:0048705 | skeletal system morphogenesis | 1.15E-07 | 1.79E-05 | 1.41E-05 | 89/1277/56603/2737/3207/4188/4256/4323/4313/579/5156/6424/6474/10736/6913/7043 |
| BP | GO:0007492 | endoderm development | 1.39E-07 | 2.05E-05 | 1.61E-05 | 1289/1290/1291/1295/2335/3624/3678/3912/4323/4313 |
| BP | GO:0010171 | body morphogenesis | 1.45E-07 | 2.05E-05 | 1.61E-05 | 1277/83716/64388/10586/4313/5156/90102/7043 |
| BP | GO:0048706 | embryonic skeletal system development | 1.63E-07 | 2.20E-05 | 1.73E-05 | 1277/1745/2737/3207/4188/4323/579/5156/6474/10736/6913/7043 |
| BP | GO:0022617 | extracellular matrix disassembly | 2.58E-07 | 3.32E-05 | 2.61E-05 | 9507/1215/1511/4035/4312/4323/4327/4313/4314 |
| BP | GO:0051216 | cartilage development | 6.15E-07 | 7.50E-05 | 5.91E-05 | 81792/11173/633/1277/2737/9464/3207/4017/4256/579/5744/6474/10736/6591 |
| BP | GO:0055123 | digestive system development | 6.33E-07 | 7.50E-05 | 5.91E-05 | 56033/79827/51339/8642/2737/3239/579/5156/6474/10736/25803/7043 |
| BP | GO:0010810 | regulation of cell-substrate adhesion | 6.96E-07 | 7.80E-05 | 6.15E-05 | 151887/1307/1277/136227/1295/11117/2199/2335/4035/4323/4653/4811/90102/5054/7057 |
| BP | GO:0034329 | cell junction assembly | 7.11E-07 | 7.80E-05 | 6.15E-05 | 89/187/140689/1009/23562/1307/8642/2335/10052/145581/4035/94030/54674/4323/4653/90102/6585/6591/27286/7057/7058 |
| BP | GO:0001763 | morphogenesis of a branching structure | 8.94E-07 | 9.46E-05 | 7.46E-05 | 8642/1909/57471/2252/2737/3082/3207/3239/3845/4323/6474/10736/9353/30812 |
| BP | GO:0001649 | osteoblast differentiation | 1.09E-06 | 0.000111 | 8.78E-05 | 1277/1291/90993/2737/9464/3082/4015/4653/5744/202151/6474/64093/6591/30812/1462 |
| BP | GO:0001822 | kidney development | 1.13E-06 | 0.000112 | 8.81E-05 | 59/185/168667/56603/8642/2200/2737/3207/8516/55083/4811/5156/5159/5176/10736/9353/30812 |
| BP | GO:1901888 | regulation of cell junction assembly | 1.44E-06 | 0.000138 | 0.000109 | 187/1307/145581/4035/94030/54674/4323/4653/90102/6585/6591/27286/7057/7058 |
| BP | GO:0072001 | renal system development | 1.71E-06 | 0.000159 | 0.000125 | 59/185/168667/56603/8642/2200/2737/3207/8516/55083/4811/5156/5159/5176/10736/9353/30812 |
| BP | GO:0001655 | urogenital system development | 1.87E-06 | 0.000168 | 0.000132 | 59/185/168667/56603/8642/2200/2737/3207/3239/8516/55083/4811/5156/5159/5176/10736/9353/30812 |
| BP | GO:0048565 | digestive tract development | 2.09E-06 | 0.000182 | 0.000143 | 79827/51339/8642/2737/3239/579/5156/6474/10736/25803/7043 |
| BP | GO:0055001 | muscle cell development | 2.49E-06 | 0.000211 | 0.000166 | 58/70/89/2318/10324/4015/4656/5156/5159/221476/5997/5999/6474 |
| BP | GO:0042476 | odontogenesis | 2.62E-06 | 0.000211 | 0.000166 | 54829/1277/1278/1745/2737/9464/3624/3912/5156/5054/7043 |
| BP | GO:0006936 | muscle contraction | 2.70E-06 | 0.000211 | 0.000166 | 58/59/70/89/775/8913/1264/1674/1909/10052/2852/23704/23630/10324/4626/4632/5592/5997 |
| BP | GO:0002062 | chondrocyte differentiation | 2.81E-06 | 0.000211 | 0.000166 | 81792/11173/2737/3207/4017/579/5744/6474/10736/6591 |
| BP | GO:0014706 | striated muscle tissue development | 2.85E-06 | 0.000211 | 0.000166 | 58/70/89/56999/8913/56603/10052/10324/4015/4223/9242/4656/5156/5159/221476/5997/5999/6474/30812 |
| BP | GO:0042692 | muscle cell differentiation | 2.85E-06 | 0.000211 | 0.000166 | 58/70/89/8038/187/56603/2318/2852/10324/3845/4015/4323/4656/5156/5159/221476/5997/5999/6474 |
| BP | GO:0042060 | wound healing | 2.98E-06 | 0.000215 | 0.00017 | 1277/1281/1289/9162/2162/2153/2335/2737/3678/4015/5156/5159/375033/90102/5592/5054/6678/7043/7057/23671 |
| BP | GO:0085029 | extracellular matrix assembly | 3.17E-06 | 0.000223 | 0.000176 | 84168/1278/11117/3036/3912/4015/90102 |
| BP | GO:0071711 | basement membrane organization | 4.34E-06 | 0.000299 | 0.000236 | 1215/2620/3912/4811/22795/90102 |
| BP | GO:0060537 | muscle tissue development | 5.72E-06 | 0.000385 | 0.000303 | 58/70/89/56999/8913/56603/10052/10324/4015/4223/9242/4656/5156/5159/221476/5997/5999/6474/30812 |
| BP | GO:0001654 | eye development | 6.84E-06 | 0.00045 | 0.000355 | 775/1289/1290/1295/1745/2200/2737/3624/11081/10586/5156/5159/5309/5176/64093/30812/55714/51384 |
| BP | GO:0060349 | bone morphogenesis | 7.21E-06 | 0.000462 | 0.000364 | 89/1277/56603/2737/3207/4323/6424/6474/7043 |
| BP | GO:0060541 | respiratory system development | 7.33E-06 | 0.000462 | 0.000364 | 9509/83716/2252/2737/3845/4015/4323/9242/5156/5159/6678/25803/7043 |
| BP | GO:0048762 | mesenchymal cell differentiation | 7.88E-06 | 0.000469 | 0.00037 | 1277/147906/1909/2335/9464/3082/4017/90102/202151/10736/6591/30812/7043/51384 |
| BP | GO:0048704 | embryonic skeletal system morphogenesis | 7.88E-06 | 0.000469 | 0.00037 | 2737/3207/4188/4323/5156/6474/10736/6913/7043 |
| BP | GO:0150063 | visual system development | 7.92E-06 | 0.000469 | 0.00037 | 775/1289/1290/1295/1745/2200/2737/3624/11081/10586/5156/5159/5309/5176/64093/30812/55714/51384 |
| BP | GO:0072132 | mesenchyme morphogenesis | 8.57E-06 | 0.000498 | 0.000393 | 58/59/70/72/187/8642/6591 |
| BP | GO:0045765 | regulation of angiogenesis | 9.02E-06 | 0.000514 | 0.000405 | 8038/56999/187/168667/1215/90993/11117/3082/140862/3678/5228/5740/5054/5176/6678/7057/7058 |
| BP | GO:0030324 | lung development | 9.28E-06 | 0.000518 | 0.000409 | 9509/83716/2252/2737/3845/4015/4323/5156/5159/6678/25803/7043 |
| BP | GO:0048880 | sensory system development | 9.83E-06 | 0.000539 | 0.000425 | 775/1289/1290/1295/1745/2200/2737/3624/11081/10586/5156/5159/5309/5176/64093/30812/55714/51384 |
| BP | GO:1901342 | regulation of vasculature development | 1.13E-05 | 0.000609 | 0.00048 | 8038/56999/187/168667/1215/90993/11117/3082/140862/3678/5228/5740/5054/5176/6678/7057/7058 |
| BP | GO:0030323 | respiratory tube development | 1.16E-05 | 0.000615 | 0.000485 | 9509/83716/2252/2737/3845/4015/4323/5156/5159/6678/25803/7043 |
| BP | GO:0061138 | morphogenesis of a branching epithelium | 1.23E-05 | 0.000639 | 0.000504 | 8642/1909/2252/2737/3082/3207/3239/3845/4323/10736/9353/30812 |
| BP | GO:0051146 | striated muscle cell differentiation | 1.44E-05 | 0.000723 | 0.00057 | 58/70/89/8038/56603/10324/3845/4323/4656/5156/5159/221476/5997/5999/6474 |
| BP | GO:0002040 | sprouting angiogenesis | 1.45E-05 | 0.000723 | 0.00057 | 56999/185/187/168667/90993/3678/4017/4223/5228/9353/27286/7057 |
| BP | GO:0007178 | transmembrane receptor protein serine/threonine kinase signaling pathway | 1.46E-05 | 0.000723 | 0.00057 | 54829/168667/55997/8483/1278/1281/1745/11117/2200/11167/64388/3624/4015/4035/6424/7043/7057 |
| BP | GO:0090092 | regulation of transmembrane receptor protein serine/threonine kinase signaling pathway | 1.97E-05 | 0.000957 | 0.000755 | 54829/168667/8483/1745/11117/2200/11167/64388/3624/4015/4035/6424/7043/7057 |
| BP | GO:0001953 | negative regulation of cell-matrix adhesion | 2.35E-05 | 0.001123 | 0.000885 | 4035/4323/4653/90102/5054/7057 |
| BP | GO:0032331 | negative regulation of chondrocyte differentiation | 2.39E-05 | 0.001123 | 0.000885 | 81792/11173/579/5744/6591 |
| BP | GO:0030574 | collagen catabolic process | 2.71E-05 | 0.001255 | 0.000989 | 9509/4312/4323/4327/4313/4314 |
| BP | GO:0001656 | metanephros development | 2.87E-05 | 0.001308 | 0.001031 | 2200/2737/3207/55083/5156/5159/10736/30812 |
| BP | GO:0090288 | negative regulation of cellular response to growth factor stimulus | 3.03E-05 | 0.001361 | 0.001073 | 81792/168667/90993/1745/11117/2200/64388/9353/7057 |
| BP | GO:0090287 | regulation of cellular response to growth factor stimulus | 3.32E-05 | 0.001469 | 0.001158 | 81792/54829/168667/90993/1745/11117/2200/11167/64388/3678/4015/6424/9353/7043/7057 |
| BP | GO:0030336 | negative regulation of cell migration | 3.72E-05 | 0.001608 | 0.001267 | 56999/1281/6387/11117/3036/4035/4223/89795/90102/127435/5592/5054/5176/9353/7057/23671 |
| BP | GO:0072006 | nephron development | 3.75E-05 | 0.001608 | 0.001267 | 59/8642/2737/3207/55083/4811/5156/5159/10736/30812 |
| BP | GO:0003170 | heart valve development | 3.81E-05 | 0.001613 | 0.001271 | 56999/187/8642/11117/6474/9353/6591 |
| BP | GO:0048546 | digestive tract morphogenesis | 4.06E-05 | 0.001694 | 0.001335 | 51339/2737/3239/5156/6474/10736 |
| BP | GO:0060325 | face morphogenesis | 4.27E-05 | 0.001756 | 0.001384 | 1277/83716/4313/5156/7043 |
| BP | GO:0048701 | embryonic cranial skeleton morphogenesis | 4.62E-05 | 0.001873 | 0.001476 | 2737/4323/5156/10736/6913/7043 |
| BP | GO:0001657 | ureteric bud development | 4.71E-05 | 0.001886 | 0.001486 | 168667/8642/2737/3207/55083/10736/9353/30812 |
| BP | GO:0072163 | mesonephric epithelium development | 5.10E-05 | 0.001969 | 0.001552 | 168667/8642/2737/3207/55083/10736/9353/30812 |
| BP | GO:0072164 | mesonephric tubule development | 5.10E-05 | 0.001969 | 0.001552 | 168667/8642/2737/3207/55083/10736/9353/30812 |
| BP | GO:0010812 | negative regulation of cell-substrate adhesion | 5.12E-05 | 0.001969 | 0.001552 | 1277/4035/4323/4653/90102/5054/7057 |
| BP | GO:0006941 | striated muscle contraction | 5.41E-05 | 0.002055 | 0.00162 | 70/89/775/8913/10052/23704/23630/10324/4626/4632/5997 |
| BP | GO:0043500 | muscle adaptation | 5.99E-05 | 0.002246 | 0.001771 | 58/89/9464/4653/4656/8013/221476/5997/5999 |
| BP | GO:2000146 | negative regulation of cell motility | 6.18E-05 | 0.002267 | 0.001787 | 56999/1281/6387/11117/3036/4035/4223/89795/90102/127435/5592/5054/5176/9353/7057/23671 |
| BP | GO:0048562 | embryonic organ morphogenesis | 6.37E-05 | 0.002267 | 0.001787 | 187/2200/2737/9464/3207/4188/4323/579/5156/6474/10736/6913/7043/51384 |
| BP | GO:0043010 | camera-type eye development | 6.40E-05 | 0.002267 | 0.001787 | 775/1295/1745/2200/2737/3624/11081/10586/5156/5159/5309/5176/30812/55714/51384 |
| BP | GO:0007044 | cell-substrate junction assembly | 6.42E-05 | 0.002267 | 0.001787 | 89/1307/2335/4035/4323/4653/90102/7057 |
| BP | GO:0048557 | embryonic digestive tract morphogenesis | 6.43E-05 | 0.002267 | 0.001787 | 2737/5156/6474/10736 |
| BP | GO:0071230 | cellular response to amino acid stimulus | 6.77E-05 | 0.002345 | 0.001849 | 1307/1277/1278/1290/1291/4313/5156 |
| BP | GO:0010811 | positive regulation of cell-substrate adhesion | 6.81E-05 | 0.002345 | 0.001849 | 151887/1307/136227/1295/11117/2199/2335/4653/4811 |
| BP | GO:0001823 | mesonephros development | 6.91E-05 | 0.002354 | 0.001855 | 168667/8642/2737/3207/55083/10736/9353/30812 |
| BP | GO:0070252 | actin-mediated cell contraction | 7.44E-05 | 0.002484 | 0.001958 | 70/775/8913/10052/23704/23630/4626/4632 |
| BP | GO:0060688 | regulation of morphogenesis of a branching structure | 7.46E-05 | 0.002484 | 0.001958 | 2252/3082/3239/6474/10736/30812 |
| BP | GO:0051271 | negative regulation of cellular component movement | 8.01E-05 | 0.002607 | 0.002055 | 56999/1281/6387/11117/3036/4035/4223/89795/90102/127435/5592/5054/5176/9353/7057/23671 |
| BP | GO:0071559 | response to transforming growth factor beta | 8.32E-05 | 0.002607 | 0.002055 | 54829/8483/7123/1277/1278/1281/1745/11117/2200/4015/25878/7043/7057 |
| BP | GO:0060323 | head morphogenesis | 8.35E-05 | 0.002607 | 0.002055 | 1277/83716/4313/5156/7043 |
| BP | GO:0061037 | negative regulation of cartilage development | 8.35E-05 | 0.002607 | 0.002055 | 81792/11173/579/5744/6591 |
| BP | GO:1901889 | negative regulation of cell junction assembly | 8.35E-05 | 0.002607 | 0.002055 | 4035/4323/90102/6585/7057 |
| BP | GO:0032964 | collagen biosynthetic process | 8.36E-05 | 0.002607 | 0.002055 | 1277/1289/90993/11117/5159/7043 |
| BP | GO:0051153 | regulation of striated muscle cell differentiation | 8.60E-05 | 0.002655 | 0.002093 | 89/56603/4323/4656/221476/5997/5999/6474 |
| BP | GO:0045165 | cell fate commitment | 9.00E-05 | 0.002749 | 0.002167 | 56603/1745/64641/2737/3207/440823/4656/5454/10736/30812/25803/6913/51384 |
| BP | GO:0150115 | cell-substrate junction organization | 9.91E-05 | 0.002996 | 0.002361 | 89/1307/2335/4035/4323/4653/90102/7057 |
| BP | GO:0010720 | positive regulation of cell development | 0.000102 | 0.00304 | 0.002396 | 89/6387/2335/2737/2852/8111/3207/4035/4653/4656/5176/6474/9353/30812 |
| BP | GO:0072028 | nephron morphogenesis | 0.000105 | 0.00304 | 0.002396 | 8642/2737/3207/55083/5159/10736/30812 |
| BP | GO:0051895 | negative regulation of focal adhesion assembly | 0.000106 | 0.00304 | 0.002396 | 4035/4323/90102/7057 |
| BP | GO:0060973 | cell migration involved in heart development | 0.000106 | 0.00304 | 0.002396 | 8642/9464/5159/6591 |
| BP | GO:0150118 | negative regulation of cell-substrate junction organization | 0.000106 | 0.00304 | 0.002396 | 4035/4323/90102/7057 |
| BP | GO:0090101 | negative regulation of transmembrane receptor protein serine/threonine kinase signaling pathway | 0.000111 | 0.003156 | 0.002488 | 54829/168667/8483/1745/11117/2200/64388/4035/7043 |
| BP | GO:0048741 | skeletal muscle fiber development | 0.000113 | 0.003179 | 0.002506 | 58/89/10324/4656/6474 |
| BP | GO:0018149 | peptide cross-linking | 0.00013 | 0.003631 | 0.002862 | 633/1281/2162/2335/7057 |
| BP | GO:0014068 | positive regulation of phosphatidylinositol 3-kinase signaling | 0.000134 | 0.003708 | 0.002923 | 2335/2852/3082/4653/5156/5159/51384 |
| BP | GO:0071229 | cellular response to acid chemical | 0.000145 | 0.003978 | 0.003135 | 1307/1277/1278/1290/1291/4313/5156 |
| BP | GO:0051962 | positive regulation of nervous system development | 0.000152 | 0.004144 | 0.003266 | 6387/2335/2737/2852/4035/94030/54674/5176/6474/9353/30812/27286/7058 |
| BP | GO:0098742 | cell-cell adhesion via plasma-membrane adhesion molecules | 0.000158 | 0.004258 | 0.003356 | 30835/1009/23562/8642/1830/147409/83872/145581/94030/54510/5100/56112/55714 |
| BP | GO:0040013 | negative regulation of locomotion | 0.000166 | 0.004436 | 0.003496 | 56999/1281/6387/11117/3036/4035/4223/89795/90102/127435/5592/5054/5176/9353/7057/23671 |
| BP | GO:0014904 | myotube cell development | 0.00017 | 0.004508 | 0.003554 | 58/89/10324/4656/6474 |
| BP | GO:0060047 | heart contraction | 0.000187 | 0.004899 | 0.003862 | 70/775/8913/1674/10052/27129/23704/23630/4632/5997/5999/6474 |
| BP | GO:0050673 | epithelial cell proliferation | 0.000191 | 0.00495 | 0.003902 | 185/187/168667/1295/6387/2252/3082/3912/4017/4323/8013/5228/5176/6591/6678/7057/51384 |
| BP | GO:0055013 | cardiac muscle cell development | 0.000212 | 0.005417 | 0.00427 | 70/5156/5159/221476/5997/5999/6474 |
| BP | GO:0060021 | roof of mouth development | 0.000212 | 0.005417 | 0.00427 | 2737/9464/3624/4223/9242/5156/7043 |
| BP | GO:0010463 | mesenchymal cell proliferation | 0.00022 | 0.005569 | 0.00439 | 8642/2252/9464/6474/10736 |
| BP | GO:0051960 | regulation of nervous system development | 0.000224 | 0.005613 | 0.004424 | 6387/1745/2335/2737/2852/3082/4035/94030/54674/5309/5176/6474/6585/9353/30812/27286/7058 |
| BP | GO:0048041 | focal adhesion assembly | 0.000245 | 0.006102 | 0.00481 | 89/1307/4035/4323/4653/90102/7057 |
| BP | GO:0072210 | metanephric nephron development | 0.000249 | 0.006129 | 0.004831 | 55083/5156/5159/10736/30812 |
| BP | GO:0060675 | ureteric bud morphogenesis | 0.00025 | 0.006129 | 0.004831 | 8642/2737/3207/55083/10736/30812 |
| BP | GO:0090130 | tissue migration | 0.000253 | 0.006149 | 0.004847 | 58/59/70/72/56999/168667/2252/11167/4017/4223/5176/9353/6678/27286/7057 |
| BP | GO:0071560 | cellular response to transforming growth factor beta stimulus | 0.000262 | 0.006307 | 0.004972 | 54829/8483/7123/1277/1278/1281/1745/11117/2200/4015/7043/7057 |
| BP | GO:0003015 | heart process | 0.000272 | 0.006466 | 0.005096 | 70/775/8913/1674/10052/27129/23704/23630/4632/5997/5999/6474 |
| BP | GO:0072171 | mesonephric tubule morphogenesis | 0.000273 | 0.006466 | 0.005096 | 8642/2737/3207/55083/10736/30812 |
| BP | GO:0014902 | myotube differentiation | 0.000275 | 0.006466 | 0.005096 | 58/89/8038/56603/10324/4323/4656/6474 |
| BP | GO:0045766 | positive regulation of angiogenesis | 0.00028 | 0.006491 | 0.005116 | 8038/187/168667/1215/3082/3678/5228/5740/5054/7057 |
| BP | GO:1904018 | positive regulation of vasculature development | 0.00028 | 0.006491 | 0.005116 | 8038/187/168667/1215/3082/3678/5228/5740/5054/7057 |
| BP | GO:0003081 | regulation of systemic arterial blood pressure by renin-angiotensin | 0.000289 | 0.00664 | 0.005234 | 185/1215/1511/56670 |
| BP | GO:0016525 | negative regulation of angiogenesis | 0.000293 | 0.006671 | 0.005258 | 56999/90993/11117/140862/5054/5176/6678/7057/7058 |
| BP | GO:0030282 | bone mineralization | 0.000309 | 0.006926 | 0.005459 | 89/54829/7123/1278/4015/4256/5744/7043 |
| BP | GO:0010172 | embryonic body morphogenesis | 0.00032 | 0.006926 | 0.005459 | 64388/10586/90102 |
| BP | GO:0048592 | eye morphogenesis | 0.000323 | 0.006926 | 0.005459 | 1289/1290/1295/2200/2737/5309/30812/55714/51384 |
| BP | GO:0048754 | branching morphogenesis of an epithelial tube | 0.000323 | 0.006926 | 0.005459 | 8642/1909/2737/3207/3845/4323/10736/9353/30812 |
| BP | GO:2000181 | negative regulation of blood vessel morphogenesis | 0.000323 | 0.006926 | 0.005459 | 56999/90993/11117/140862/5054/5176/6678/7057/7058 |
| BP | GO:0055006 | cardiac cell development | 0.000324 | 0.006926 | 0.005459 | 70/5156/5159/221476/5997/5999/6474 |
| BP | GO:1905330 | regulation of morphogenesis of an epithelium | 0.000325 | 0.006926 | 0.005459 | 81792/2252/3082/3239/10736/30812 |
| BP | GO:0090596 | sensory organ morphogenesis | 0.000325 | 0.006926 | 0.005459 | 1289/1290/1295/56603/2200/2737/579/5309/10736/30812/55714/51384 |
| BP | GO:1903522 | regulation of blood circulation | 0.000325 | 0.006926 | 0.005459 | 185/775/8913/1674/10052/3274/27129/23704/23630/5997/5999/6474 |
| BP | GO:1901343 | negative regulation of vasculature development | 0.000339 | 0.007178 | 0.005658 | 56999/90993/11117/140862/5054/5176/6678/7057/7058 |
| BP | GO:0051893 | regulation of focal adhesion assembly | 0.000353 | 0.007361 | 0.005802 | 1307/4035/4323/4653/90102/7057 |
| BP | GO:0090109 | regulation of cell-substrate junction assembly | 0.000353 | 0.007361 | 0.005802 | 1307/4035/4323/4653/90102/7057 |
| BP | GO:0060993 | kidney morphogenesis | 0.00037 | 0.007658 | 0.006037 | 8642/2737/3207/55083/5159/10736/30812 |
| BP | GO:0010517 | regulation of phospholipase activity | 0.000383 | 0.007879 | 0.006211 | 185/1909/4035/5156/5159/5997 |
| BP | GO:0060324 | face development | 0.000392 | 0.007893 | 0.006222 | 1277/83716/4313/5156/7043 |
| BP | GO:0007519 | skeletal muscle tissue development | 0.000392 | 0.007893 | 0.006222 | 58/89/56603/10324/4223/9242/4656/6474/30812 |
| BP | GO:0051147 | regulation of muscle cell differentiation | 0.000392 | 0.007893 | 0.006222 | 89/56603/2852/4323/4656/221476/5997/5999/6474 |
| BP | GO:0035051 | cardiocyte differentiation | 0.000411 | 0.008198 | 0.006462 | 70/2852/9464/5156/5159/221476/5997/5999/6474 |
| BP | GO:0042698 | ovulation cycle | 0.000415 | 0.008198 | 0.006462 | 2620/3624/5156/5176/9353/7043 |
| BP | GO:1904888 | cranial skeletal system development | 0.000415 | 0.008198 | 0.006462 | 2737/4323/5156/10736/6913/7043 |
| BP | GO:0072378 | blood coagulation | 10/18723 | 0.008454 | 0.063076 | 0.049718329 |
| BP | GO:0071492 | cellular response to UV-A | 0.000435 | 0.008485 | 0.006688 | 4312/4313/4314 |
| BP | GO:0022602 | ovulation cycle process | 0.000435 | 0.008485 | 0.006688 | 2620/3624/5156/9353/7043 |
| BP | GO:0043501 | skeletal muscle adaptation | 0.000472 | 0.00908 | 0.007157 | 58/89/4653/4656 |
| BP | GO:0072273 | metanephric nephron morphogenesis | 0.000472 | 0.00908 | 0.007157 | 55083/5159/10736/30812 |
| BP | GO:0030048 | actin filament-based movement | 0.000479 | 0.009145 | 0.007208 | 70/775/8913/10052/23704/23630/4626/4632 |
| BP | GO:0010830 | regulation of myotube differentiation | 0.000483 | 0.009169 | 0.007227 | 89/56603/4323/4656/6474 |
| BP | GO:0043502 | regulation of muscle adaptation | 0.000508 | 0.009591 | 0.00756 | 89/9464/4656/8013/221476/5997/5999 |
| BP | GO:0072078 | nephron tubule morphogenesis | 0.000524 | 0.00975 | 0.007685 | 8642/2737/3207/55083/10736/30812 |
| BP | GO:0150116 | regulation of cell-substrate junction organization | 0.000524 | 0.00975 | 0.007685 | 1307/4035/4323/4653/90102/7057 |
| BP | GO:0031032 | actomyosin structure organization | 0.000527 | 0.00975 | 0.007685 | 58/70/1264/10324/4653/5156/5159/90102/7043/23671 |
| BP | GO:0048742 | regulation of skeletal muscle fiber development | 0.000574 | 0.010371 | 0.008175 | 89/4656/6474 |
| BP | GO:0061052 | negative regulation of cell growth involved in cardiac muscle cell development | 0.000574 | 0.010371 | 0.008175 | 221476/5997/5999 |
| BP | GO:0086067 | AV node cell to bundle of His cell communication | 0.000574 | 0.010371 | 0.008175 | 775/8913/10052 |
| BP | GO:0060840 | artery development | 0.000574 | 0.010371 | 0.008175 | 56999/187/2737/9464/4015/4035/5159 |
| BP | GO:1903053 | regulation of extracellular matrix organization | 0.000589 | 0.010517 | 0.00829 | 165/84168/11117/4035/90102 |
| BP | GO:2001238 | positive regulation of extrinsic apoptotic signaling pathway | 0.000589 | 0.010517 | 0.00829 | 2852/3624/8406/7057/7078 |
| BP | GO:0048738 | cardiac muscle tissue development | 0.0006 | 0.01065 | 0.008395 | 70/56999/8913/10052/9242/5156/5159/221476/5997/5999/6474 |
| BP | GO:0072088 | nephron epithelium morphogenesis | 0.000609 | 0.010729 | 0.008457 | 8642/2737/3207/55083/10736/30812 |
| BP | GO:0071772 | response to BMP | 0.000617 | 0.010751 | 0.008474 | 81792/11173/168667/1745/2200/11167/64388/6424/7043 |
| BP | GO:0071773 | cellular response to BMP stimulus | 0.000617 | 0.010751 | 0.008474 | 81792/11173/168667/1745/2200/11167/64388/6424/7043 |
| BP | GO:0060538 | skeletal muscle organ development | 0.000644 | 0.011163 | 0.008799 | 58/89/56603/10324/4223/9242/4656/6474/30812 |
| BP | GO:0007156 | homophilic cell adhesion via plasma membrane adhesion molecules | 0.000673 | 0.011587 | 0.009133 | 1009/8642/1830/147409/83872/54510/5100/56112/55714 |
| BP | GO:0061333 | renal tubule morphogenesis | 0.000703 | 0.012036 | 0.009487 | 8642/2737/3207/55083/10736/30812 |
| BP | GO:0031214 | biomineral tissue development | 0.000733 | 0.012472 | 0.009831 | 89/54829/7123/1277/1278/4015/4256/5744/7043 |
| BP | GO:0010755 | regulation of plasminogen activation | 0.000738 | 0.012487 | 0.009843 | 7123/5054/7057 |
| BP | GO:0072073 | kidney epithelium development | 0.000753 | 0.012673 | 0.009989 | 168667/8642/2737/3207/55083/10736/9353/30812 |
| BP | GO:0030178 | negative regulation of Wnt signaling pathway | 0.000764 | 0.012788 | 0.01008 | 56033/51339/147906/2737/3090/4035/4188/6424/6591 |
| BP | GO:0008016 | regulation of heart contraction | 0.000774 | 0.012885 | 0.010156 | 775/8913/1674/10052/27129/23704/23630/5997/5999/6474 |
| BP | GO:0060048 | cardiac muscle contraction | 0.00079 | 0.013073 | 0.010304 | 70/775/8913/10052/23704/23630/4632/5997 |
| BP | GO:0110148 | biomineralization | 0.000797 | 0.013112 | 0.010335 | 89/54829/7123/1277/1278/4015/4256/5744/7043 |
| BP | GO:0048736 | appendage development | 0.000831 | 0.013468 | 0.010616 | 775/56603/2737/9464/3207/3239/4223/6474/64093 |
| BP | GO:0060173 | limb development | 0.000831 | 0.013468 | 0.010616 | 775/56603/2737/9464/3207/3239/4223/6474/64093 |
| BP | GO:0060562 | epithelial tube morphogenesis | 0.000832 | 0.013468 | 0.010616 | 81792/187/8642/1909/2737/9464/3207/55083/3845/4323/10736/9353/30812 |
| BP | GO:0007517 | muscle organ development | 0.00088 | 0.014169 | 0.011168 | 58/70/89/1293/56603/2115/10324/4015/4223/9242/4656/6474/30812 |
| BP | GO:0035791 | platelet-derived growth factor receptor-beta signaling pathway | 0.000929 | 0.014796 | 0.011662 | 4015/4035/5159 |
| BP | GO:0070141 | response to UV-A | 0.000929 | 0.014796 | 0.011662 | 4312/4313/4314 |
| BP | GO:0048566 | embryonic digestive tract development | 0.00094 | 0.014882 | 0.011731 | 2737/5156/6474/10736 |
| BP | GO:0007422 | peripheral nervous system development | 0.000989 | 0.01558 | 0.01228 | 2115/9464/4653/5453/5454/30812 |
| BP | GO:0086002 | cardiac muscle cell action potential involved in contraction | 0.001016 | 0.015917 | 0.012546 | 775/8913/10052/23704/23630 |
| BP | GO:0090257 | regulation of muscle system process | 0.001028 | 0.016029 | 0.012634 | 89/775/1264/2852/9464/4656/8013/221476/5592/5997/5999 |
| BP | GO:0051149 | positive regulation of muscle cell differentiation | 0.001055 | 0.016297 | 0.012845 | 89/56603/2852/4323/4656/6474 |
| BP | GO:0003338 | metanephros morphogenesis | 0.001062 | 0.016297 | 0.012845 | 55083/5159/10736/30812 |
| BP | GO:0009954 | proximal/distal pattern formation | 0.001062 | 0.016297 | 0.012845 | 56603/1745/2737/3207 |
| BP | GO:0014066 | regulation of phosphatidylinositol 3-kinase signaling | 0.001067 | 0.016298 | 0.012846 | 2335/2852/3082/4653/5156/5159/51384 |
| BP | GO:0014065 | phosphatidylinositol 3-kinase signaling | 0.001092 | 0.016589 | 0.013076 | 2335/2852/3082/4653/5156/5159/375033/51384 |
| BP | GO:0003179 | heart valve morphogenesis | 0.001104 | 0.01669 | 0.013155 | 56999/8642/11117/9353/6591 |
| BP | GO:0030900 | forebrain development | 0.001144 | 0.01712 | 0.013494 | 1281/6387/1745/2737/10642/3624/3845/3912/4035/5453/5454/5592/6585/9353 |
| BP | GO:0033275 | actin-myosin filament sliding | 0.001149 | 0.01712 | 0.013494 | 70/4626/4632 |
| BP | GO:0007596 | blood coagulation | 0.00115 | 0.01712 | 0.013494 | 1281/9162/2162/2153/2335/5156/375033/5592/5054/7057 |
| BP | GO:0048333 | mesodermal cell differentiation | 0.001195 | 0.017406 | 0.01372 | 3207/3624/8516/10736 |
| BP | GO:0001658 | branching involved in ureteric bud morphogenesis | 0.001199 | 0.017406 | 0.01372 | 8642/2737/3207/10736/30812 |
| BP | GO:0022029 | telencephalon cell migration | 0.001199 | 0.017406 | 0.01372 | 6387/2737/3912/5454/9353 |
| BP | GO:0048008 | platelet-derived growth factor receptor signaling pathway | 0.001199 | 0.017406 | 0.01372 | 4015/4035/8013/5156/5159 |
| BP | GO:0050886 | endocrine process | 0.001199 | 0.017406 | 0.01372 | 185/1215/1511/3624/3845/56670 |
| BP | GO:0051155 | positive regulation of striated muscle cell differentiation | 0.001299 | 0.018768 | 0.014793 | 89/56603/4323/4656/6474 |
| BP | GO:0008406 | gonad development | 0.001319 | 0.018969 | 0.014952 | 2620/3207/3624/4323/5156/5159/22836/9353/30812/7425 |
| BP | GO:0044319 | wound healing, spreading of cells | 0.001339 | 0.018975 | 0.014957 | 1289/3678/90102/23671 |
| BP | GO:0086019 | cell-cell signaling involved in cardiac conduction | 0.001339 | 0.018975 | 0.014957 | 775/8913/10052/23630 |
| BP | GO:0090505 | epiboly involved in wound healing | 0.001339 | 0.018975 | 0.014957 | 1289/3678/90102/23671 |
| BP | GO:0042063 | gliogenesis | 0.001345 | 0.018975 | 0.014957 | 1745/2737/3845/3912/4035/4323/4653/5309/5453/5454/56978/30812 |
| BP | GO:0007599 | hemostasis | 0.001365 | 0.019006 | 0.014981 | 1281/9162/2162/2153/2335/5156/375033/5592/5054/7057 |
| BP | GO:0050817 | coagulation | 0.001365 | 0.019006 | 0.014981 | 1281/9162/2162/2153/2335/5156/375033/5592/5054/7057 |
| BP | GO:0030326 | embryonic limb morphogenesis | 0.00138 | 0.019006 | 0.014981 | 775/56603/2737/9464/3207/3239/6474 |
| BP | GO:0035113 | embryonic appendage morphogenesis | 0.00138 | 0.019006 | 0.014981 | 775/56603/2737/9464/3207/3239/6474 |
| BP | GO:0043200 | response to amino acid | 0.00138 | 0.019006 | 0.014981 | 1307/1277/1278/1290/1291/4313/5156 |
| BP | GO:0001991 | regulation of systemic arterial blood pressure by circulatory renin-angiotensin | 0.0014 | 0.019006 | 0.014981 | 1215/1511/56670 |
| BP | GO:1901201 | regulation of extracellular matrix assembly | 0.0014 | 0.019006 | 0.014981 | 84168/11117/90102 |
| BP | GO:0035904 | aorta development | 0.001405 | 0.019006 | 0.014981 | 56999/187/4015/4035/5159 |
| BP | GO:0010466 | negative regulation of peptidase activity | 0.001405 | 0.019006 | 0.014981 | 1293/1447/3082/4035/51050/221476/140902/5054/5176/7057/7078 |
| BP | GO:0007162 | negative regulation of cell adhesion | 0.001423 | 0.019156 | 0.015099 | 339390/1277/6387/2737/4035/4323/4653/90102/5592/5054/6591/7057 |
| BP | GO:0043588 | skin development | 0.001448 | 0.019414 | 0.015302 | 9509/1277/1278/1281/1289/1290/56603/2252/3624/5453/51384 |
| BP | GO:2001236 | regulation of extrinsic apoptotic signaling pathway | 0.00148 | 0.01975 | 0.015567 | 2852/3082/3624/5054/6591/8406/7057/7078 |
| BP | GO:0090504 | epiboly | 0.001495 | 0.019861 | 0.015655 | 1289/3678/90102/23671 |
| BP | GO:0021885 | forebrain cell migration | 0.001518 | 0.020068 | 0.015818 | 6387/2737/3912/5454/9353 |
| BP | GO:0007389 | pattern specification process | 0.001561 | 0.020549 | 0.016197 | 187/56033/55997/56603/8642/1745/2737/9464/3207/3239/4188/4223/579/5100/10736 |
| BP | GO:0045137 | development of primary sexual characteristics | 0.00161 | 0.021105 | 0.016635 | 2620/3207/3624/4323/5156/5159/22836/9353/30812/7425 |
| BP | GO:1903670 | regulation of sprouting angiogenesis | 0.001637 | 0.021356 | 0.016833 | 56999/168667/90993/3678/7057 |
| BP | GO:0010831 | positive regulation of myotube differentiation | 0.001682 | 0.021751 | 0.017145 | 56603/4323/4656 |
| BP | GO:0055003 | cardiac myofibril assembly | 0.001682 | 0.021751 | 0.017145 | 70/5156/5159 |
| BP | GO:0014910 | regulation of smooth muscle cell migration | 0.001719 | 0.022144 | 0.017455 | 4035/8013/5159/5592/5054/9353 |
| BP | GO:0032970 | regulation of actin filament-based process | 0.001767 | 0.022661 | 0.017862 | 775/6387/163782/4035/54551/4653/5156/5159/90102/5999/22836/9353/7043/23671 |
| BP | GO:0007015 | actin filament organization | 0.001783 | 0.022759 | 0.01794 | 58/70/6387/57471/2620/163782/54551/4653/90102/5999/22836/9353/7043/23671/165904 |
| BP | GO:1904705 | regulation of vascular associated smooth muscle cell proliferation | 0.001821 | 0.023146 | 0.018244 | 1264/2852/4313/8013/5592/7043 |
| BP | GO:0001990 | regulation of systemic arterial blood pressure by hormone | 0.001844 | 0.023248 | 0.018325 | 185/1215/1511/56670 |
| BP | GO:0016202 | regulation of striated muscle tissue development | 0.001844 | 0.023248 | 0.018325 | 89/4015/4656/6474 |
| BP | GO:0001935 | endothelial cell proliferation | 0.001854 | 0.023265 | 0.018338 | 185/187/168667/6387/4017/4323/5228/6678/7057 |
| BP | GO:0001837 | epithelial to mesenchymal transition | 0.001894 | 0.023583 | 0.018589 | 1277/147906/3082/4017/90102/6591/7043/51384 |
| BP | GO:0060393 | regulation of pathway-restricted SMAD protein phosphorylation | 0.001895 | 0.023583 | 0.018589 | 168667/11117/3624/4035/7043 |
| BP | GO:0060191 | regulation of lipase activity | 0.001926 | 0.023676 | 0.018662 | 185/1909/4035/5156/5159/5997 |
| BP | GO:0072080 | nephron tubule development | 0.001926 | 0.023676 | 0.018662 | 8642/2737/3207/55083/10736/30812 |
| BP | GO:1990874 | vascular associated smooth muscle cell proliferation | 0.001926 | 0.023676 | 0.018662 | 1264/2852/4313/8013/5592/7043 |
| BP | GO:0032956 | regulation of actin cytoskeleton organization | 0.001979 | 0.024141 | 0.019029 | 6387/163782/4035/54551/4653/5156/5159/90102/5999/22836/9353/7043/23671 |
| BP | GO:0030728 | ovulation | 0.001997 | 0.024141 | 0.019029 | 2620/3624/5997 |
| BP | GO:0048643 | positive regulation of skeletal muscle tissue development | 0.001997 | 0.024141 | 0.019029 | 89/4656/6474 |
| BP | GO:0097094 | craniofacial suture morphogenesis | 0.001997 | 0.024141 | 0.019029 | 2737/4323/7043 |
| BP | GO:0030239 | myofibril assembly | 0.002035 | 0.024423 | 0.019251 | 58/70/10324/5156/5159 |
| BP | GO:0001708 | cell fate specification | 0.002037 | 0.024423 | 0.019251 | 2737/3207/440823/5454/10736/6913 |
| BP | GO:0007548 | sex differentiation | 0.002117 | 0.025205 | 0.019867 | 2620/3207/3239/3624/4323/5156/5159/22836/9353/30812/7425 |
| BP | GO:0055007 | cardiac muscle cell differentiation | 0.002119 | 0.025205 | 0.019867 | 70/5156/5159/221476/5997/5999/6474 |
| BP | GO:0055002 | striated muscle cell development | 0.002182 | 0.025851 | 0.020377 | 58/70/10324/5156/5159 |
| BP | GO:0086091 | regulation of heart rate by cardiac conduction | 0.002246 | 0.026405 | 0.020813 | 775/8913/23704/23630 |
| BP | GO:1901861 | regulation of muscle tissue development | 0.002246 | 0.026405 | 0.020813 | 89/4015/4656/6474 |
| BP | GO:0061326 | renal tubule development | 0.002271 | 0.026592 | 0.020961 | 8642/2737/3207/55083/10736/30812 |
| BP | GO:0060389 | pathway-restricted SMAD protein phosphorylation | 0.002337 | 0.026834 | 0.021151 | 168667/11117/3624/4035/7043 |
| BP | GO:0035313 | wound healing, spreading of epidermal cells | 0.002346 | 0.026834 | 0.021151 | 1289/3678/90102 |
| BP | GO:0072077 | renal vesicle morphogenesis | 0.002346 | 0.026834 | 0.021151 | 55083/10736/30812 |
| BP | GO:0086014 | atrial cardiac muscle cell action potential | 0.002346 | 0.026834 | 0.021151 | 775/10052/23630 |
| BP | GO:0086026 | atrial cardiac muscle cell to AV node cell signaling | 0.002346 | 0.026834 | 0.021151 | 775/10052/23630 |
| BP | GO:0086066 | atrial cardiac muscle cell to AV node cell communication | 0.002346 | 0.026834 | 0.021151 | 775/10052/23630 |
| BP | GO:0008585 | female gonad development | 0.002396 | 0.027298 | 0.021517 | 2620/3624/4323/5156/9353/7425 |
| BP | GO:0001952 | regulation of cell-matrix adhesion | 0.002423 | 0.027499 | 0.021675 | 1307/4035/4323/4653/90102/5054/7057 |
| BP | GO:0050918 | positive chemotaxis | 0.002499 | 0.028255 | 0.022272 | 6387/2252/3082/4035/5228 |
| BP | GO:0030510 | regulation of BMP signaling pathway | 0.002526 | 0.028448 | 0.022424 | 168667/1745/2200/11167/64388/6424 |
| BP | GO:0046661 | male sex differentiation | 0.002581 | 0.028954 | 0.022822 | 3207/3239/3624/4323/5156/5159/22836/30812 |
| BP | GO:0014909 | smooth muscle cell migration | 0.002661 | 0.02968 | 0.023395 | 4035/8013/5159/5592/5054/9353 |
| BP | GO:0052547 | regulation of peptidase activity | 0.002665 | 0.02968 | 0.023395 | 84168/1293/1447/2335/2852/3082/4035/5118/51050/221476/140902/5054/5176/7057/7078 |
| BP | GO:0048634 | regulation of muscle organ development | 0.002705 | 0.029746 | 0.023447 | 89/4015/4656/6474 |
| BP | GO:0032332 | positive regulation of chondrocyte differentiation | 0.002732 | 0.029746 | 0.023447 | 2737/3207/4017 |
| BP | GO:0043586 | tongue development | 0.002732 | 0.029746 | 0.023447 | 56603/2737/9464 |
| BP | GO:0045844 | positive regulation of striated muscle tissue development | 0.002732 | 0.029746 | 0.023447 | 89/4656/6474 |
| BP | GO:0048636 | positive regulation of muscle organ development | 0.002732 | 0.029746 | 0.023447 | 89/4656/6474 |
| BP | GO:0072087 | renal vesicle development | 0.002732 | 0.029746 | 0.023447 | 55083/10736/30812 |
| BP | GO:1903844 | regulation of cellular response to transforming growth factor beta stimulus | 0.00276 | 0.029945 | 0.023604 | 54829/1745/11117/2200/4015/7043/7057 |
| BP | GO:1902904 | negative regulation of supramolecular fiber organization | 0.00278 | 0.030048 | 0.023685 | 11117/163782/4653/89795/90102/9353/11075/23671 |
| BP | GO:0014912 | negative regulation of smooth muscle cell migration | 0.002957 | 0.031504 | 0.024832 | 4035/5592/5054/9353 |
| BP | GO:0021795 | cerebral cortex cell migration | 0.002957 | 0.031504 | 0.024832 | 2737/3912/5454/9353 |
| BP | GO:0032965 | regulation of collagen biosynthetic process | 0.002957 | 0.031504 | 0.024832 | 90993/11117/5159/7043 |
| BP | GO:1904037 | positive regulation of epithelial cell apoptotic process | 0.002957 | 0.031504 | 0.024832 | 57124/2852/6424/7057 |
| BP | GO:0021543 | pallium development | 0.00299 | 0.031747 | 0.025024 | 1281/1745/2737/10642/3912/4035/5454/9353 |
| BP | GO:0010611 | regulation of cardiac muscle hypertrophy | 0.003036 | 0.03212 | 0.025318 | 9464/8013/221476/5997/5999 |
| BP | GO:0021537 | telencephalon development | 0.003064 | 0.032297 | 0.025457 | 1281/6387/1745/2737/10642/3624/3912/4035/5454/9353 |
| BP | GO:0046545 | development of primary female sexual characteristics | 0.003099 | 0.032548 | 0.025655 | 2620/3624/4323/5156/9353/7425 |
| BP | GO:1901863 | positive regulation of muscle tissue development | 0.003154 | 0.033006 | 0.026016 | 89/4656/6474 |
| BP | GO:0048608 | reproductive structure development | 0.003212 | 0.033375 | 0.026307 | 2620/2737/3207/3239/3624/4188/4323/5156/5159/22836/5176/9353/30812/7425 |
| BP | GO:1904706 | negative regulation of vascular associated smooth muscle cell proliferation | 0.003224 | 0.033375 | 0.026307 | 1264/2852/5592/7043 |
| BP | GO:0050878 | regulation of body fluid levels | 0.003234 | 0.033375 | 0.026307 | 187/1281/1447/56603/9162/2162/2153/2335/5156/375033/5592/5054/7057 |
| BP | GO:0051346 | negative regulation of hydrolase activity | 0.003234 | 0.033375 | 0.026307 | 815/1293/1447/3082/4035/51050/221476/140902/5997/5054/5176/7057/7078 |
| BP | GO:0051963 | regulation of synapse assembly | 0.003256 | 0.03345 | 0.026366 | 145581/94030/54674/6585/27286/7058 |
| BP | GO:0001101 | response to acid chemical | 0.003264 | 0.03345 | 0.026366 | 1307/1277/1278/1290/1291/4313/5156 |
| BP | GO:0014897 | striated muscle hypertrophy | 0.003419 | 0.034686 | 0.027341 | 9464/4653/8013/221476/5997/5999 |
| BP | GO:0048568 | embryonic organ development | 0.003419 | 0.034686 | 0.027341 | 187/2200/2737/9464/3207/4188/4323/579/5156/6474/10736/6913/7043/51384 |
| BP | GO:0061458 | reproductive system development | 0.003419 | 0.034686 | 0.027341 | 2620/2737/3207/3239/3624/4188/4323/5156/5159/22836/5176/9353/30812/7425 |
| BP | GO:0001667 | ameboidal-type cell migration | 0.003525 | 0.035603 | 0.028064 | 56999/168667/2252/2335/11167/9464/3036/4017/4223/5176/9353/30812/6678/27286/7057 |
| BP | GO:1902903 | regulation of supramolecular fiber organization | 0.003534 | 0.035603 | 0.028064 | 165/6387/11117/163782/54551/4653/89795/90102/5999/9353/11075/7043/23671 |
| BP | GO:0002027 | regulation of heart rate | 0.003588 | 0.036027 | 0.028398 | 775/8913/23704/23630/5999/6474 |
| BP | GO:0032891 | negative regulation of organic acid transport | 0.003613 | 0.036157 | 0.0285 | 5997/5999/7057 |
| BP | GO:0014743 | regulation of muscle hypertrophy | 0.003652 | 0.0363 | 0.028613 | 9464/8013/221476/5997/5999 |
| BP | GO:0033627 | cell adhesion mediated by integrin | 0.003652 | 0.0363 | 0.028613 | 1307/2200/3678/5054/6591 |
| BP | GO:0035107 | appendage morphogenesis | 0.003685 | 0.036387 | 0.028681 | 775/56603/2737/9464/3207/3239/6474 |
| BP | GO:0035108 | limb morphogenesis | 0.003685 | 0.036387 | 0.028681 | 775/56603/2737/9464/3207/3239/6474 |
| BP | GO:0014896 | muscle hypertrophy | 0.003763 | 0.03691 | 0.029094 | 9464/4653/8013/221476/5997/5999 |
| BP | GO:1901890 | positive regulation of cell junction assembly | 0.003763 | 0.03691 | 0.029094 | 1307/94030/54674/4653/27286/7058 |
| BP | GO:0086003 | cardiac muscle cell contraction | 0.003876 | 0.037889 | 0.029865 | 775/8913/10052/23704/23630 |
| BP | GO:0048015 | phosphatidylinositol-mediated signaling | 0.004097 | 0.039372 | 0.031034 | 2335/2852/3082/4653/5156/5159/375033/51384 |
| BP | GO:0021544 | subpallium development | 0.004112 | 0.039372 | 0.031034 | 1745/2737/3624 |
| BP | GO:0061050 | regulation of cell growth involved in cardiac muscle cell development | 0.004112 | 0.039372 | 0.031034 | 221476/5997/5999 |
| BP | GO:0007157 | heterophilic cell-cell adhesion via plasma membrane cell adhesion molecules | 0.004124 | 0.039372 | 0.031034 | 30835/8642/83872/55714 |
| BP | GO:0060976 | coronary vasculature development | 0.004124 | 0.039372 | 0.031034 | 187/2852/9464/5159 |
| BP | GO:0008584 | male gonad development | 0.004147 | 0.039372 | 0.031034 | 3207/3624/4323/5156/5159/22836/30812 |
| BP | GO:0035296 | regulation of tube diameter | 0.004147 | 0.039372 | 0.031034 | 59/185/1909/2852/3274/5592/5997 |
| BP | GO:0050921 | positive regulation of chemotaxis | 0.004147 | 0.039372 | 0.031034 | 6387/5159/5228/5054/9353/56670/7057 |
| BP | GO:0097746 | blood vessel diameter maintenance | 0.004147 | 0.039372 | 0.031034 | 59/185/1909/2852/3274/5592/5997 |
| BP | GO:0045785 | positive regulation of cell adhesion | 0.004192 | 0.039674 | 0.031272 | 151887/30835/1307/136227/1295/6387/11117/2199/2335/2737/55083/4653/4811/8013 |
| BP | GO:0035150 | regulation of tube size | 0.004311 | 0.040533 | 0.031949 | 59/185/1909/2852/3274/5592/5997 |
| BP | GO:0046546 | development of primary male sexual characteristics | 0.004311 | 0.040533 | 0.031949 | 3207/3624/4323/5156/5159/22836/30812 |
| BP | GO:0010927 | cellular component assembly involved in morphogenesis | 0.004327 | 0.04056 | 0.031971 | 58/70/10324/5156/5159/90102 |
| BP | GO:0048660 | regulation of smooth muscle cell proliferation | 0.004381 | 0.040937 | 0.032268 | 1264/2852/4313/8013/5159/5592/7043/7057 |
| BP | GO:0003044 | regulation of systemic arterial blood pressure mediated by a chemical signal | 0.004457 | 0.04126 | 0.032522 | 185/1215/1511/56670 |
| BP | GO:0010712 | regulation of collagen metabolic process | 0.004457 | 0.04126 | 0.032522 | 90993/11117/5159/7043 |
| BP | GO:0048483 | autonomic nervous system development | 0.004457 | 0.04126 | 0.032522 | 1909/2335/9464/30812 |
| BP | GO:0034446 | substrate adhesion-dependent cell spreading | 0.004528 | 0.041784 | 0.032935 | 84168/2335/8516/3912/4653/23671 |
| BP | GO:0031100 | animal organ regeneration | 0.004606 | 0.042123 | 0.033202 | 6387/2737/3082/8013/5228 |
| BP | GO:0086001 | cardiac muscle cell action potential | 0.004606 | 0.042123 | 0.033202 | 775/8913/10052/23704/23630 |
| BP | GO:0003018 | vascular process in circulatory system | 0.004633 | 0.042123 | 0.033202 | 59/185/1909/2852/3274/4035/5592/5997/6581/9353 |
| BP | GO:0031639 | plasminogen activation | 0.00465 | 0.042123 | 0.033202 | 7123/5054/7057 |
| BP | GO:0048641 | regulation of skeletal muscle tissue development | 0.00465 | 0.042123 | 0.033202 | 89/4656/6474 |
| BP | GO:2000353 | positive regulation of endothelial cell apoptotic process | 0.00465 | 0.042123 | 0.033202 | 57124/2852/7057 |
| BP | GO:0048017 | inositol lipid-mediated signaling | 0.00468 | 0.042264 | 0.033313 | 2335/2852/3082/4653/5156/5159/375033/51384 |
| BP | GO:0022411 | cellular component disassembly | 0.004721 | 0.042501 | 0.0335 | 9507/1215/1511/2852/4035/4312/4323/4327/4313/4314/89795/6591/11075/7043 |
| BP | GO:0072009 | nephron epithelium development | 0.004736 | 0.04251 | 0.033508 | 8642/2737/3207/55083/10736/30812 |
| BP | GO:0006939 | smooth muscle contraction | 0.004951 | 0.044171 | 0.034817 | 59/1264/1909/2852/5592/5997 |
| BP | GO:0014812 | muscle cell migration | 0.004951 | 0.044171 | 0.034817 | 4035/8013/5159/5592/5054/9353 |
| BP | GO:0048659 | smooth muscle cell proliferation | 0.004994 | 0.044425 | 0.035017 | 1264/2852/4313/8013/5159/5592/7043/7057 |
| BP | GO:0060326 | cell chemotaxis | 0.005102 | 0.044896 | 0.035389 | 185/6387/6374/3082/4015/5156/5159/5228/5054/9353/7057 |
| BP | GO:0010001 | glial cell differentiation | 0.005126 | 0.044896 | 0.035389 | 1745/2737/3845/4035/4653/5453/5454/56978/30812 |
| BP | GO:0050769 | positive regulation of neurogenesis | 0.005126 | 0.044896 | 0.035389 | 6387/2335/2737/2852/4035/5176/6474/9353/30812 |
| BP | GO:0061045 | negative regulation of wound healing | 0.005144 | 0.044896 | 0.035389 | 5156/90102/5592/5054/7057 |
| BP | GO:0030195 | negative regulation of blood coagulation | 0.005179 | 0.044896 | 0.035389 | 5156/5592/5054/7057 |
| BP | GO:1902041 | regulation of extrinsic apoptotic signaling pathway via death domain receptors | 0.005179 | 0.044896 | 0.035389 | 3082/5054/7057/7078 |
| BP | GO:0021846 | cell proliferation in forebrain | 0.005229 | 0.044896 | 0.035389 | 2737/10642/5454 |
| BP | GO:0033622 | integrin activation | 0.005229 | 0.044896 | 0.035389 | 1307/6387/2335 |
| BP | GO:0072012 | glomerulus vasculature development | 0.005229 | 0.044896 | 0.035389 | 59/5156/5159 |
| BP | GO:0099560 | synaptic membrane adhesion | 0.005229 | 0.044896 | 0.035389 | 145581/94030/5100 |
| BP | GO:1903792 | negative regulation of anion transport | 0.005229 | 0.044896 | 0.035389 | 5997/5999/6424 |
| BP | GO:1905048 | regulation of metallopeptidase activity | 0.005229 | 0.044896 | 0.035389 | 84168/4035/7078 |
| BP | GO:0050772 | positive regulation of axonogenesis | 0.005429 | 0.046477 | 0.036634 | 6387/2335/4035/6474/9353 |
| BP | GO:1900047 | negative regulation of hemostasis | 0.005567 | 0.047523 | 0.037459 | 5156/5592/5054/7057 |
| CC | GO:0062023 | collagen-containing extracellular matrix | 1.73E-37 | 4.59E-35 | 4.10E-35 | 81794/9509/9507/56999/165/23452/54829/633/140689/151887/8483/7123/1215/1307/1277/1278/84570/136227/1281/1289/1290/1291/1292/1293/1295/1511/6387/11117/2162/2199/2200/2335/83872/3912/4017/4256/4313/25878/4653/4811/22795/5118/127435/5054/5176/64093/6678/8406/27286/284297/7043/7057/7058/7078/23671/1462 |
| CC | GO:0005581 | collagen trimer | 3.35E-16 | 4.43E-14 | 3.96E-14 | 114898/114904/114905/1307/1277/1278/84570/136227/1281/1289/1290/1291/1292/1293/1295/81035/11117/4015 |
| CC | GO:0005604 | basement membrane | 7.72E-12 | 6.82E-10 | 6.09E-10 | 151887/1289/1295/2200/2335/83872/3912/4017/4811/22795/5176/64093/6678/7058/23671 |
| CC | GO:0005788 | endoplasmic reticulum lumen | 1.87E-10 | 1.24E-08 | 1.11E-08 | 11173/340075/1307/1277/1278/84570/136227/1281/1289/1290/1291/1292/1293/1295/2153/2200/2335/11167/3912/283208/7057/1462/7425 |
| CC | GO:0005583 | fibrillar collagen trimer | 7.34E-09 | 3.24E-07 | 2.90E-07 | 1307/1277/1278/1281/1289/1290 |
| CC | GO:0098643 | banded collagen fibril | 7.34E-09 | 3.24E-07 | 2.90E-07 | 1307/1277/1278/1281/1289/1290 |
| CC | GO:0098644 | complex of collagen trimers | 1.18E-08 | 4.47E-07 | 3.99E-07 | 1307/1277/1278/1281/1289/1290/1295 |
| CC | GO:0031093 | platelet alpha granule lumen | 5.30E-06 | 0.000175 | 0.000157 | 2162/2153/2335/3082/5054/6678/7043/7057 |
| CC | GO:0031091 | platelet alpha granule | 6.64E-06 | 0.000195 | 0.000175 | 2162/2153/2335/3082/5054/6678/7043/7057/7058 |
| CC | GO:0005869 | dynactin complex | 6.73E-05 | 0.001783 | 0.001593 | 58/59/70/72 |
| CC | GO:0043292 | contractile fiber | 0.000193 | 0.004659 | 0.004164 | 58/59/70/89/775/1674/2318/10324/4313/4626/4632/27295 |
| CC | GO:0030017 | sarcomere | 0.000258 | 0.005692 | 0.005087 | 58/70/89/775/1674/2318/10324/4313/4626/4632/27295 |
| CC | GO:0030016 | myofibril | 0.000555 | 0.011313 | 0.010111 | 58/70/89/775/1674/2318/10324/4313/4626/4632/27295 |
| CC | GO:0042383 | sarcolemma | 0.000894 | 0.016273 | 0.014544 | 633/775/1291/1292/1293/1674/2318/7043 |
| CC | GO:0030175 | filopodium | 0.000921 | 0.016273 | 0.014544 | 58/59/70/72/84168/57471/10642 |
| CC | GO:0034774 | secretory granule lumen | 0.002603 | 0.043114 | 0.038533 | 7123/83716/1511/2162/2153/2335/3082/5054/6678/7043/7057/7078 |
| CC | GO:0060205 | cytoplasmic vesicle lumen | 0.002807 | 0.043429 | 0.038815 | 7123/83716/1511/2162/2153/2335/3082/5054/6678/7043/7057/7078 |
| CC | GO:0031983 | vesicle lumen | 0.00295 | 0.043429 | 0.038815 | 7123/83716/1511/2162/2153/2335/3082/5054/6678/7043/7057/7078 |
| MF | GO:0005201 | extracellular matrix structural constituent | 1.35E-27 | 4.86E-25 | 4.24E-25 | 165/54829/633/8483/1307/1277/1278/84570/1281/1289/1290/1291/1292/1293/1295/11117/2199/2200/2335/83872/3912/4256/25878/4811/22795/5118/127435/6678/8406/27286/7057/7058/1462 |
| MF | GO:0005539 | glycosaminoglycan binding | 3.06E-12 | 5.50E-10 | 4.80E-10 | 633/151887/7123/84570/1289/83716/1511/2200/2252/2335/11167/64388/10894/1463/5118/5228/6585/9353/64093/7057/7058/1462 |
| MF | GO:0030020 | extracellular matrix structural constituent conferring tensile strength | 1.07E-11 | 1.28E-09 | 1.12E-09 | 1307/1277/1278/84570/1281/1289/1290/1291/1292/1293/1295 |
| MF | GO:0005518 | collagen binding | 1.81E-11 | 1.63E-09 | 1.42E-09 | 165/84168/54829/1291/1292/2335/4015/4811/22795/5118/127435/6678/7057 |
| MF | GO:0008201 | heparin binding | 3.81E-11 | 2.39E-09 | 2.08E-09 | 151887/7123/84570/1289/83716/1511/2200/2252/2335/11167/64388/5118/5228/6585/9353/64093/7057/7058 |
| MF | GO:0048407 | platelet-derived growth factor binding | 3.98E-11 | 2.39E-09 | 2.08E-09 | 1277/1278/1281/1289/1291/5156/5159 |
| MF | GO:0050840 | extracellular matrix binding | 4.16E-10 | 2.14E-08 | 1.87E-08 | 633/57124/1755/2199/4811/25903/9353/64093/6678/284297/7057 |
| MF | GO:0004222 | metalloendopeptidase activity | 8.00E-10 | 3.60E-08 | 3.14E-08 | 8038/8745/81794/81792/9509/9507/11173/56999/9635/4312/4323/4327/4313/4314 |
| MF | GO:1901681 | sulfur compound binding | 1.24E-08 | 4.94E-07 | 4.31E-07 | 151887/7123/84570/1289/83716/1511/2200/2252/2335/11167/64388/4035/5118/5228/6585/9353/64093/7057/7058 |
| MF | GO:0008237 | metallopeptidase activity | 1.26E-07 | 4.53E-06 | 3.94E-06 | 8038/8745/81794/81792/9509/9507/11173/56999/9635/1358/4312/4323/4327/4313/4314 |
| MF | GO:0005178 | integrin binding | 1.32E-06 | 4.31E-05 | 3.75E-05 | 89/8745/1307/1281/1289/6387/11117/2200/2335/3912/4323/7057 |
| MF | GO:0043394 | proteoglycan binding | 1.20E-05 | 0.000361 | 0.000315 | 1289/2335/4035/4811/9353/7057 |
| MF | GO:0019838 | growth factor binding | 4.14E-05 | 0.001145 | 0.000998 | 1277/1278/1281/1289/1291/5156/5159/27286/7043/7057 |
| MF | GO:0005044 | scavenger receptor activity | 5.81E-05 | 0.001494 | 0.001301 | 81035/1755/4017/4035/286133/284297 |
| MF | GO:0004175 | endopeptidase activity | 0.000197 | 0.004724 | 0.004117 | 8038/8745/81794/81792/9509/9507/11173/56999/9635/1215/11330/1511/4312/4323/4327/4313/4314 |
| MF | GO:0030021 | extracellular matrix structural constituent conferring compression resistance | 0.00026 | 0.005845 | 0.005093 | 54829/633/127435/1462 |
| MF | GO:0097493 | structural molecule activity conferring elasticity | 0.00046 | 0.009734 | 0.008482 | 11117/2199/2200 |
| MF | GO:0001968 | fibronectin binding | 0.000679 | 0.012865 | 0.01121 | 151887/4653/284297/7057 |
| MF | GO:0043236 | laminin binding | 0.000679 | 0.012865 | 0.01121 | 4811/9353/284297/7057 |
| MF | GO:0002020 | protease binding | 0.000811 | 0.014604 | 0.012725 | 9507/1277/1278/1281/2335/4035/5054/7078 |
| MF | GO:0038024 | cargo receptor activity | 0.000892 | 0.015293 | 0.013326 | 81035/1755/4017/4035/286133/284297 |
| MF | GO:0005161 | platelet-derived growth factor receptor binding | 0.001214 | 0.019867 | 0.017311 | 3678/5156/5159 |
| MF | GO:0061134 | peptidase regulator activity | 0.002038 | 0.031902 | 0.027798 | 1293/1447/2335/5118/51050/221476/140902/5054/5176/7078 |
| MF | GO:0005516 | calmodulin binding | 0.002771 | 0.041563 | 0.036215 | 165/775/815/1264/2669/4626/6004/5997/5999 |
