# Supplementary material for: Prognostic risk factors of serous ovarian carcinoma based on mesenchymal stem cell phenotype and guidance for therapeutic efficacy
Source: J Transl Med. 2023 Jul 11;21:456. doi: 10.1186/s12967-023-04284-3 (PMC10334653; doi:10.1186/s12967-023-04284-3)
Supplement: Supplementary file 10 — Additional file 10. KEGG pathway analysis between low and high MSC score group. KEGG pathway analysis of DEGs between groups with low and high MSC scores. [file 12967_2023_4284_MOESM10_ESM.docx]

**Additional file** **10** KEGG pathway analysis between low and high MSC score group

| **ID** | **Description** | **pvalue** | **p.adjust** | **qvalue** | **geneID** |
| --- | --- | --- | --- | --- | --- |
| hsa04974 | Protein digestion and absorption | 9.83E-10 | 1.85E-07 | 1.47E-07 | 1307/1277/1278/84570/136227/1281/1289/1290/1291/1292/1293/1295/1358 |
| hsa04512 | ECM-receptor interaction | 1.82E-09 | 1.85E-07 | 1.47E-07 | 1277/1278/1291/1292/1293/2335/3678/8516/3912/9899/7057/7058 |
| hsa04510 | Focal adhesion | 9.95E-09 | 6.74E-07 | 5.38E-07 | 1277/1278/1291/1292/1293/2318/2335/3082/3678/8516/3912/5156/5159/5228/7057/7058 |
| hsa04151 | PI3K-Akt signaling pathway | 2.24E-07 | 1.14E-05 | 9.07E-06 | 1277/1278/1291/1292/1293/90993/2252/2335/3082/3678/8516/3845/3912/2846/5156/5159/5228/7057/7058 |
| hsa04933 | AGE-RAGE signaling pathway in diabetic complications | 7.56E-06 | 0.000307 | 0.000245 | 185/1277/1278/1281/2335/3845/4313/5054/7043 |
| hsa05205 | Proteoglycans in cancer | 1.96E-05 | 0.000663 | 0.00053 | 815/1277/1278/2318/2335/3082/3678/3845/4313/7057/7078/51384 |
| hsa05165 | Human papillomavirus infection | 3.64E-05 | 0.001056 | 0.000843 | 1277/1278/1291/1292/1293/90993/2335/3678/8516/3845/3912/5159/7057/7058/51384 |
| hsa04926 | Relaxin signaling pathway | 0.000346 | 0.008249 | 0.006587 | 59/1277/1278/1281/90993/3845/4312/4313 |
| hsa04020 | Calcium signaling pathway | 0.000388 | 0.008249 | 0.006587 | 185/775/8913/51806/815/1909/2252/3082/3274/5156/5159 |
| hsa05146 | Amoebiasis | 0.000445 | 0.008249 | 0.006587 | 1277/1278/1281/1511/2335/3912/7043 |
| hsa04270 | Vascular smooth muscle contraction | 0.000447 | 0.008249 | 0.006587 | 59/72/185/775/51806/1909/5320/5592 |
| hsa05144 | Malaria | 0.000545 | 0.009214 | 0.007358 | 3082/4035/7043/7057/7058 |
| hsa05410 | Hypertrophic cardiomyopathy | 0.001338 | 0.020897 | 0.016687 | 70/775/1674/3678/8516/7043 |
| hsa04912 | GnRH signaling pathway | 0.001586 | 0.022332 | 0.017833 | 775/51806/815/3845/4323/4313 |
| hsa05415 | Diabetic cardiomyopathy | 0.001683 | 0.022332 | 0.017833 | 185/815/1215/1277/1278/1281/9945/4313/7043 |
| hsa05414 | Dilated cardiomyopathy | 0.001866 | 0.022332 | 0.017833 | 70/775/1674/3678/8516/7043 |
| hsa05215 | Prostate cancer | 0.001968 | 0.022332 | 0.017833 | 90993/3845/4314/5156/5159/6935 |
| hsa04925 | Aldosterone synthesis and secretion | 0.002073 | 0.022332 | 0.017833 | 185/775/8913/51806/815/90993 |
| hsa04015 | Rap1 signaling pathway | 0.002125 | 0.022332 | 0.017833 | 51806/2252/3082/3845/2846/5156/5159/5228/7057 |
| hsa05219 | Bladder cancer | 0.0022 | 0.022332 | 0.017833 | 3845/4312/4313/7057 |
| hsa04924 | Renin secretion | 0.002357 | 0.022784 | 0.018194 | 185/775/51806/9635/1909 |
| hsa04810 | Regulation of actin cytoskeleton | 0.002737 | 0.025082 | 0.020029 | 6387/2252/2335/3678/8516/3845/2846/5156/5159 |
| hsa05218 | Melanoma | 0.002842 | 0.025082 | 0.020029 | 2252/3082/3845/5156/5159 |
| hsa05214 | Glioma | 0.003395 | 0.028604 | 0.022842 | 51806/815/3845/5156/5159 |
| hsa04614 | Renin-angiotensin system | 0.003523 | 0.028604 | 0.022842 | 185/1215/1511 |
| hsa05412 | Arrhythmogenic right ventricular cardiomyopathy | 0.003805 | 0.029705 | 0.023721 | 89/775/1674/3678/8516 |
| hsa04010 | MAPK signaling pathway | 0.0064 | 0.048116 | 0.038423 | 775/8913/2252/2318/3082/3845/5156/5159/5228/7043 |
