# Supplementary material for: Prognostic risk factors of serous ovarian carcinoma based on mesenchymal stem cell phenotype and guidance for therapeutic efficacy
Source: J Transl Med. 2023 Jul 11;21:456. doi: 10.1186/s12967-023-04284-3 (PMC10334653; doi:10.1186/s12967-023-04284-3)
Supplement: Supplementary file 11 — Additional file 11. Genes related to prognosis in brown module. Prognostic genes in module correlated with MSC score. [file 12967_2023_4284_MOESM11_ESM.docx]

**Additional file 11** Genes related to prognosis in brown module

| **Genes** | **HR** | **HR.95L** | **HR.95H** | **pvalue** |
| --- | --- | --- | --- | --- |
| PDE2A | 1.184778 | 1.010013 | 1.389783 | 0.037314 |
| AKAP12 | 1.287257 | 1.066711 | 1.553403 | 0.008452 |
| ARHGAP6 | 1.142116 | 1.007425 | 1.294814 | 0.037939 |
| NDNF | 1.1036 | 1.018983 | 1.195244 | 0.015436 |
| GALNT15 | 1.129394 | 1.003637 | 1.270909 | 0.043358 |
| SMOC2 | 1.236301 | 1.022273 | 1.495139 | 0.028736 |
| SLC22A3 | 1.087512 | 1.004547 | 1.177328 | 0.038263 |
| LRRC17 | 1.16229 | 1.000085 | 1.350802 | 0.049871 |
| NRK | 1.089276 | 1.006036 | 1.179404 | 0.035002 |
| MMP17 | 1.147557 | 1.034039 | 1.273537 | 0.009603 |
| OGN | 1.106329 | 1.009758 | 1.212136 | 0.030133 |
| PER1 | 1.399031 | 1.044232 | 1.87438 | 0.02445 |
| COL16A1 | 1.237902 | 1.012471 | 1.513524 | 0.037451 |
