# Supplementary material for: Prognostic risk factors of serous ovarian carcinoma based on mesenchymal stem cell phenotype and guidance for therapeutic efficacy
Source: J Transl Med. 2023 Jul 11;21:456. doi: 10.1186/s12967-023-04284-3 (PMC10334653; doi:10.1186/s12967-023-04284-3)
Supplement: Supplementary file 12 — Additional file 12. Genes in the MSC score related prognostic model. Multivariate Cox regression analysis was conducted to identify MSC-score-related genes associated with OS and three genes (MMP17, AKAP12, andPER1) were used to construct the prognostic model. [file 12967_2023_4284_MOESM12_ESM.docx]

**Additional file 12** Genes in the MSC score related prognostic model

| **Genes** | **coef** | **HR** | **HR.95L** | **HR.95H** | **pvalue** |
| --- | --- | --- | --- | --- | --- |
| AKAP12 | 0.145457 | 1.156568 | 1.011076 | 1.322997 | 0.03396 |
| MMP17 | 0.122203 | 1.129984 | 0.979431 | 1.303679 | 0.09392 |
| PER1 | 0.124154 | 1.13219 | 0.985383 | 1.30087 | 0.079748 |
