# Supplementary material for: Prognostic risk factors of serous ovarian carcinoma based on mesenchymal stem cell phenotype and guidance for therapeutic efficacy
Source: J Transl Med. 2023 Jul 11;21:456. doi: 10.1186/s12967-023-04284-3 (PMC10334653; doi:10.1186/s12967-023-04284-3)
Supplement: Supplementary file 13 — Additional file 13. C-index of TCGA cohort, GEO cohorts and nomogram. Standard error, minimum value, maximum value and P value of C-index of TCGA cohort, GEO cohorts and nomogram. [file 12967_2023_4284_MOESM13_ESM.docx]

**Additional file 13** C-index of TCGA cohort, GEO cohorts and nomogram

| **Group** | **Cindex** | **Cindex$se** | **Cindex$lower** | **Cindex$upper** | **Cindex$pvalue** |
| --- | --- | --- | --- | --- | --- |
| TCGA group | 0.5783767 | 0.021304 | 0.5366217 | 0.6201316 | 0.0002342 |
| Agilent group | 0.5600322 | 0.022844 | 0.5152585 | 0.604806 | 0.0085913 |
| Affymetrix group | 0.5828117 | 0.024335 | 0.5351156 | 0.6305078 | 0.0006666 |
| Nomogram | 0.6378611 | 0.019969 | 0.5987219 | 0.6770003 | 5.07E-12 |

Cindex$se, standard error of C-index; Cindex$lower, minimal value of C-index; Cindex$upper, maximum value of C-index; Cindex$pvalue, *P* value of C-index.
