# Supplementary material for: Prognostic risk factors of serous ovarian carcinoma based on mesenchymal stem cell phenotype and guidance for therapeutic efficacy
Source: J Transl Med. 2023 Jul 11;21:456. doi: 10.1186/s12967-023-04284-3 (PMC10334653; doi:10.1186/s12967-023-04284-3)
Supplement: Supplementary file 14 — Additional file 14. Difference between staining index of PER1, AKAP12 and MMP17 and age among SOC samples. Difference between staining index of PER1, AKAP12 and MMP17 and age among SOC samples via Mann-Whitney U test. [file 12967_2023_4284_MOESM14_ESM.docx]

**Additional file 14** Difference between staining index of PER1, AKAP12 and MMP17 and age among SOC samples

|  | Staining index | | Mann-Whitney U test | |
| --- | --- | --- | --- | --- |
|  | SOC with age <= 50 | SOC with age > 50 | *Z* value | *P* Value |
| PER1 | 8.08±2.64 | 8.52±3.65 | -1.532 | 0.126 |
| AKAP12 | 10.24±2.47 | 9.97±2.98 | -0.222 | 0.825 |
| MMP17 | 8.58±2.87 | 8.63±3.06 | -0.092 | 0.927 |
