# Supplementary material for: Prognostic risk factors of serous ovarian carcinoma based on mesenchymal stem cell phenotype and guidance for therapeutic efficacy
Source: J Transl Med. 2023 Jul 11;21:456. doi: 10.1186/s12967-023-04284-3 (PMC10334653; doi:10.1186/s12967-023-04284-3)
Supplement: Supplementary file 15 — Additional file 15. Difference between staining index of PER1, AKAP12 and MMP17 and tumor size among SOC samples. Difference between staining index of PER1, AKAP12 and MMP17 and tumor size among SOC samples via Mann-Whitney U test. [file 12967_2023_4284_MOESM15_ESM.docx]

**Additional file 15** Difference between staining index of PER1, AKAP12 and MMP17 and tumor size among SOC samples

|  | Staining index | | Mann-Whitney U test | |
| --- | --- | --- | --- | --- |
|  | SOC with d <= 5cm | SOC with d > 5cm | *Z* value | *P* Value |
| PER1 | 7.75±4.03 | 7.68±3.67 | -0.033 | 0.974 |
| AKAP12 | 9.00±3.55 | 8.44±3.68 | -0.376 | 0.707 |
| MMP17 | 7.13±4.12 | 8.06±3.22 | -0.723 | 0.47 |

d <= 5cm, diameter of tumor less than or equal to 5cm; d > 5cm diameter of tumor more than 5cm.
