# Supplementary material for: Prognostic risk factors of serous ovarian carcinoma based on mesenchymal stem cell phenotype and guidance for therapeutic efficacy
Source: J Transl Med. 2023 Jul 11;21:456. doi: 10.1186/s12967-023-04284-3 (PMC10334653; doi:10.1186/s12967-023-04284-3)
Supplement: Supplementary file 16 — Additional file 16: Figure S1. Estimation of the best cutoff value for the MSC scores determined by the X-tile software. The interface of estimation of the best cutoff value for the MSC scores in TCGA-OV cohort by the X-tile software. Figure S2. Identification of the MSC-score-related gene set. (A) Clustering dendrograms to reject outliers. (B) Scale-free network confirmation with connectivity value k. (C) Module dendrogram and (D) gene dendrogram before and after combination of modules with similar expression patterns. Figure S3. Gene expression heatmap, risk scores and OS time distribution Gene expression heatmap, risk scores and OS time distribution of TCGA and GEO cohorts, and ROC curve of GEO datasets. Figure S4. Immunity inhibition factors and genetic characteristics in different MSC risk group. The level of immunity inhibition factors and genetic characteristics in different MSC risk group, which P value was more than 0.05. Figure S5. Estimation of the best cutoff value for the GSE19061 risk scores determined by X-tile software. The interface of estimation of the best cutoff value for the GSE19061 risk scores determined by X-tile software. Figure S6. Kaplan-Meier analysis of other biomarkers of GSE91061. To evaluate the predictive power of other biomarkers, PD-L1 and PD-1 expression status, 14 kinds of tumor-infiltrating lymphocytes, mutational burden, and immune gene signatures were correlation with the survival. Figure S7. Correlation between the abundance score of biomarkers and response to immunotherapy. The scores of other biomarkers in different response groups of GSE19061 and the proportion of patients with response to anti-PD-1 immunotherapy in different biomarker level groups. Figure S8. Evaluation of the prognostic model in the antiangiogenesis dataset and construction of the nomogram. Evaluation of the prognostic model in the antiangiogenesis dataset and conformity between nomogram prediction and actual observation in terms of the 1-(C), 3-(D), and [file 12967_2023_4284_MOESM16_ESM.docx]

**Additional figure**

**
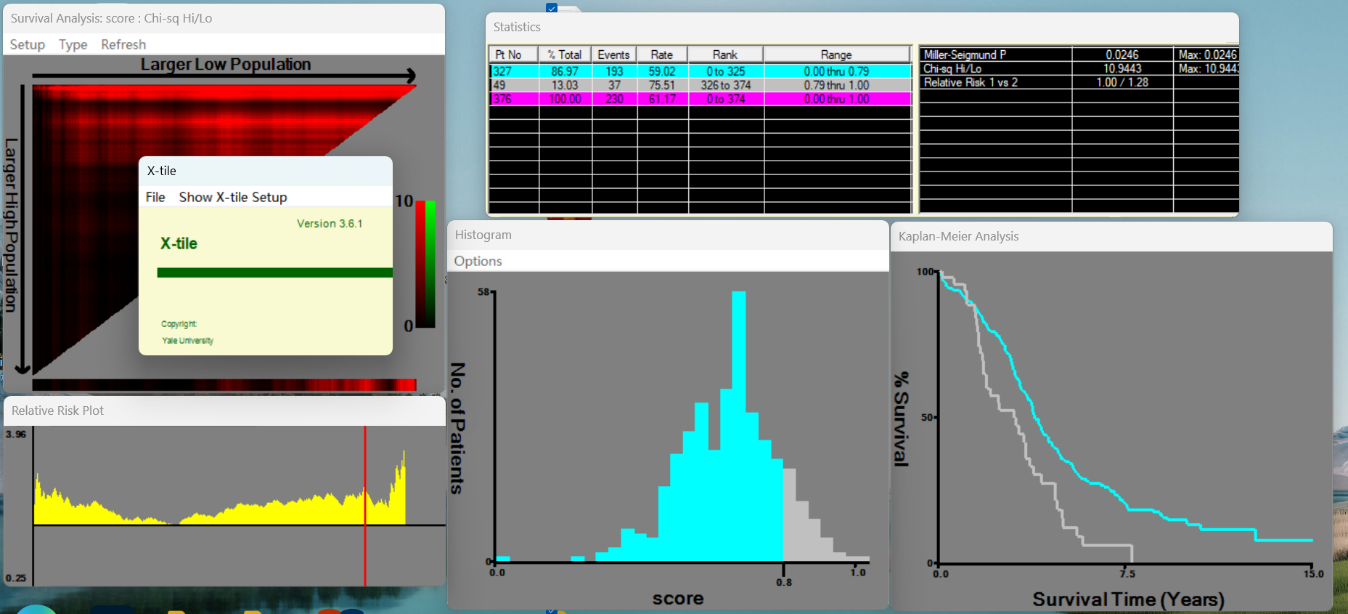
**

**Figure S1** Estimation of the best cutoff value for the MSC scores of TCGA-OV cohort determined by the X-tile software. When 0.79 was set as the cut off value, the overall survival of patients in these two groups are markedly different (*P*=0.0246)

**
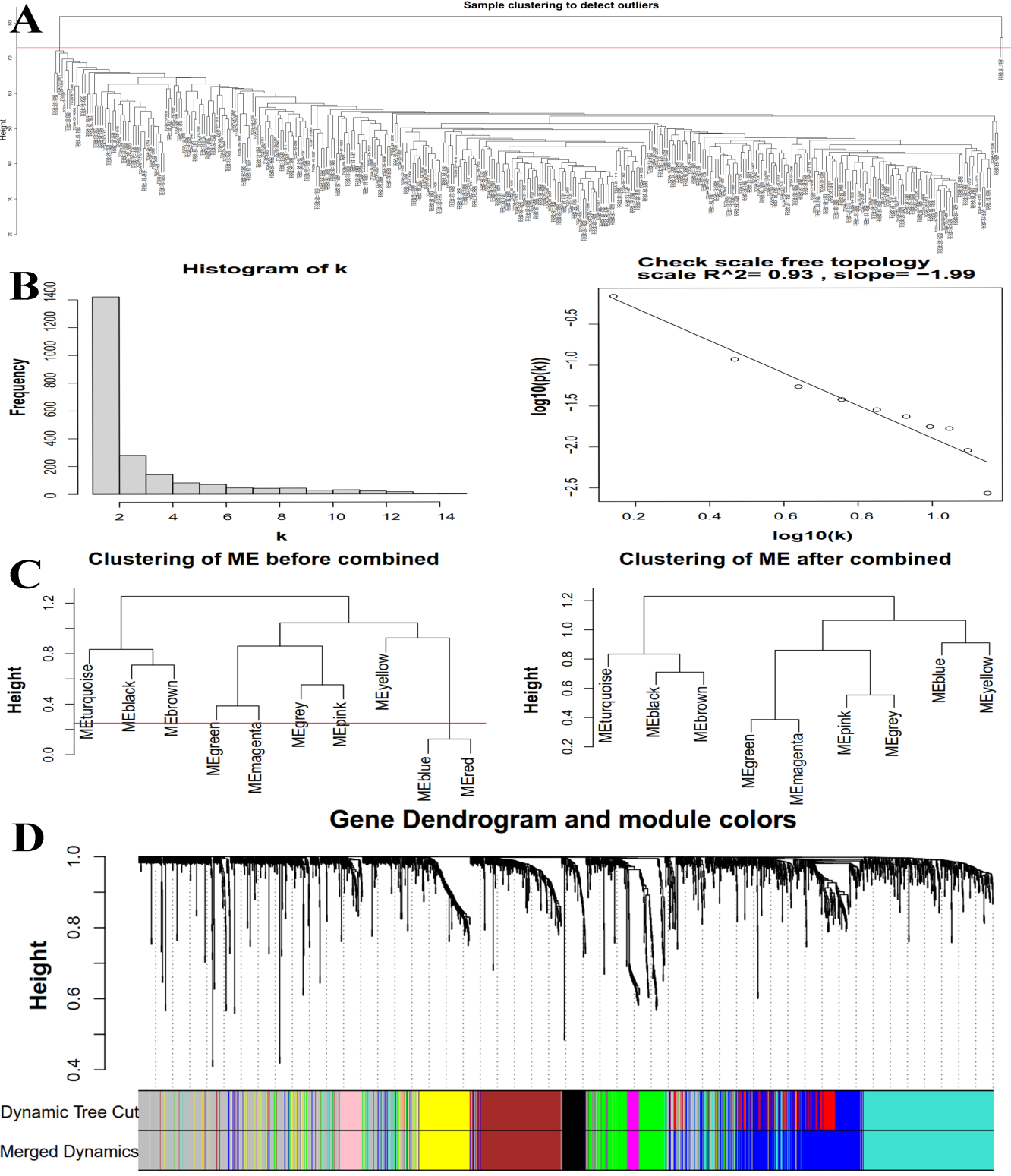
**

**Figure S2** Identification of the MSC-score-related gene set of TCGA-OV cohort. **(A)** Clustering dendrograms to reject outliers, and there are two outliers when height is 73. **(B)** Scale-free network confirmation with connectivity value k when soft threshold is 6. Before and after combination of modules with similar expression patterns displayed with **(C)** module dendrogram and **(D)** gene dendrogram based on a dissimilarity measure (1-TOM). And blue and red module eigengenes were combined.

**
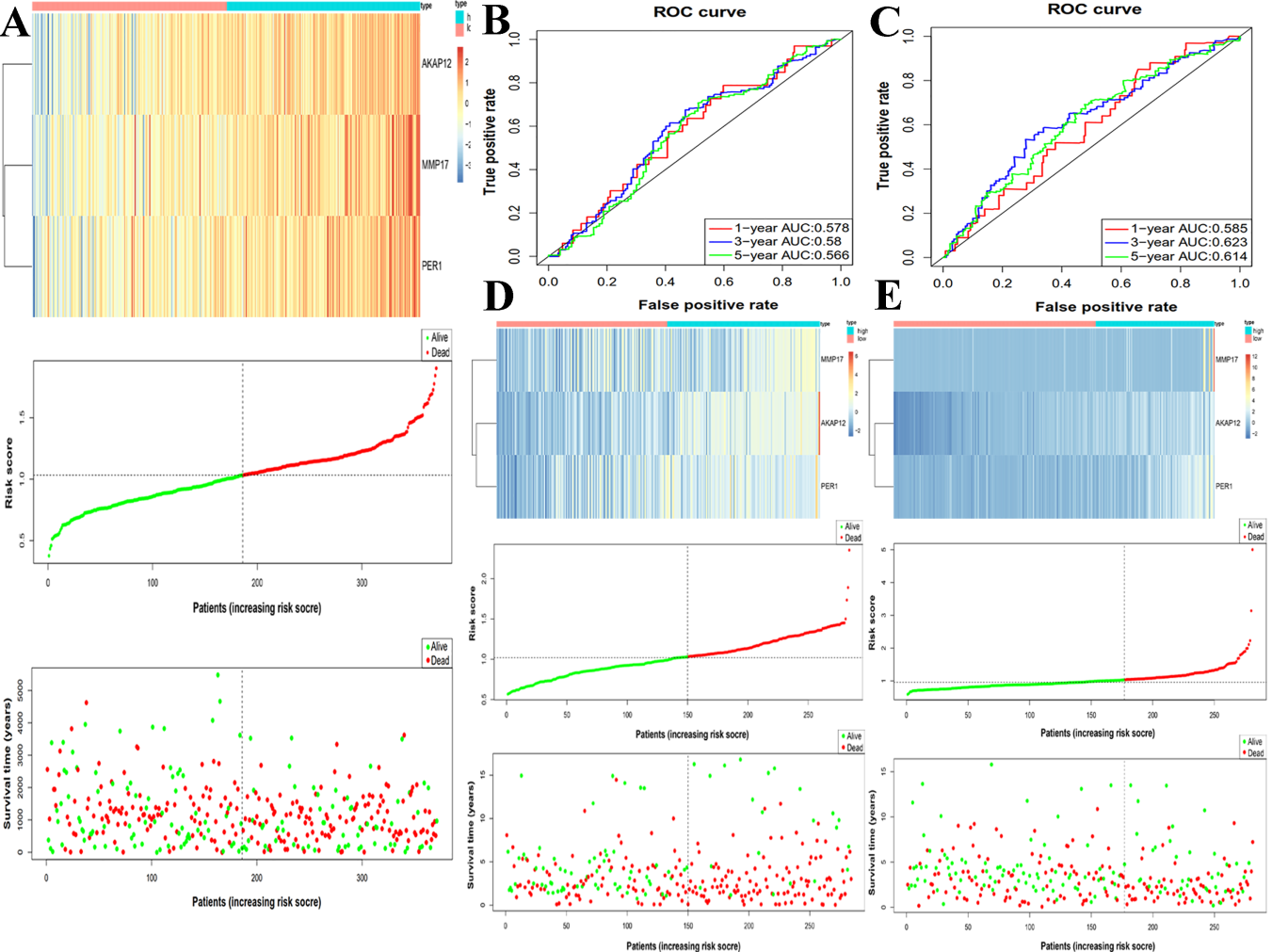
**

**Figure S3 (A)** Gene expression heatmap, risk scores and OS time distribution of the TCGA cohort. ROC curve and AUC value of the prognostic model in the validation cohort **(B)** GSE17260 and GSE53963 from GPL6480 and **(C)** GSE26712, GSE14764, GSE23554 from GPL96. Gene expression heatmap, risk scores, and OS time distribution of **(D)** GSE17260 and GSE53963 from GPL6480 and **(E)** GSE26712, GSE14764 and GSE23554 from GPL96**.** The pink bar stands for high-risk group and the blue bar is low-risk group. The redder of the bar represents the higher gene expression. The red and green circle represents dead and alive status, respectively. ROC, receiver operating characteristic; AUC, area under the curve; PER1, period circadian regulator 1; AKAP12, a-kinase anchoring protein 12; MMP17, matrix metallopeptidase 17.

**
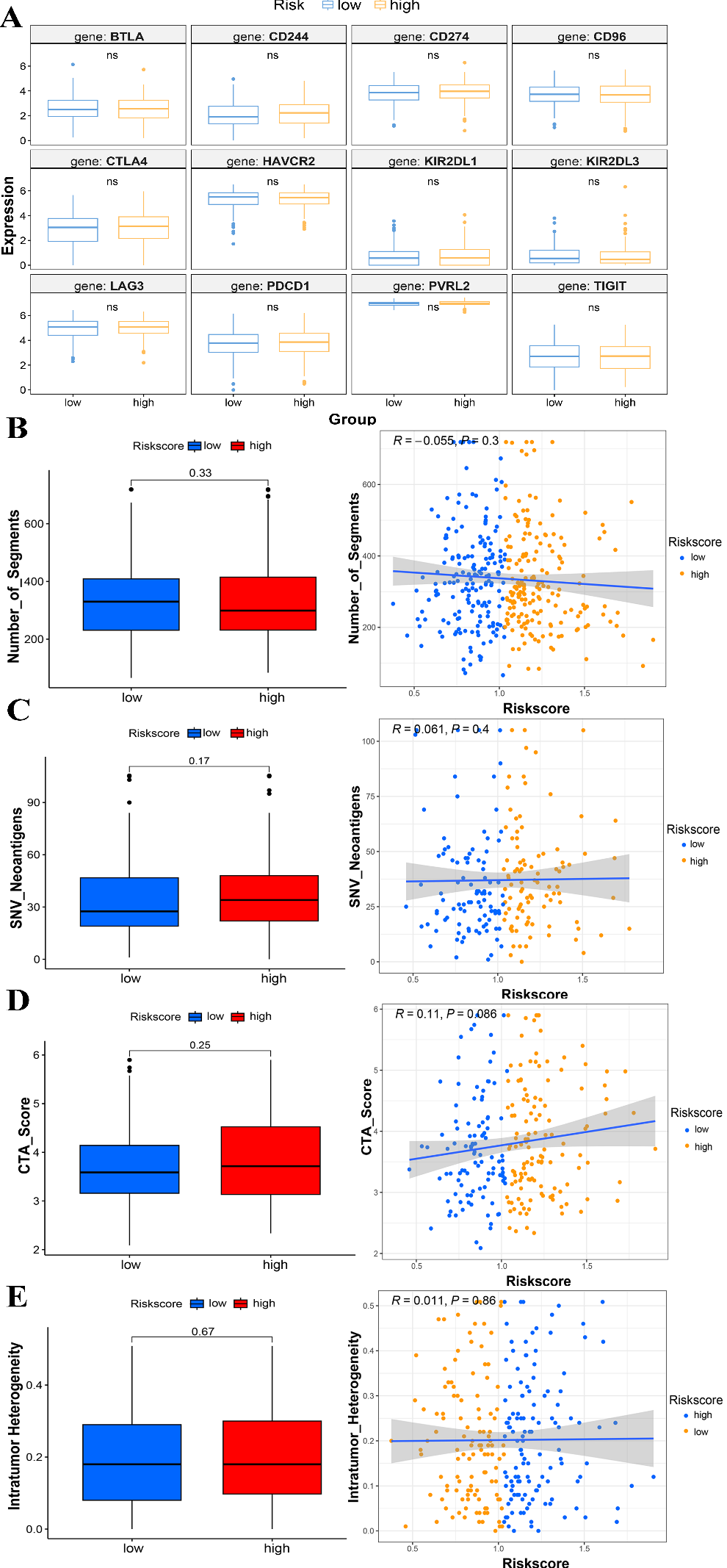
**

**Figure S4 (A)** Expression of differential immunity inhibition factors of *P* > 0.05 in different risk groups of TCGA cohort. Alteration of **(B)** segment number of CNV, **(C)** SNV neoantigen, **(D)** CTA scores**,** and **(E)** intratumor heterogeneity of TCGA cohort**.** R means correlation coefficient.

**
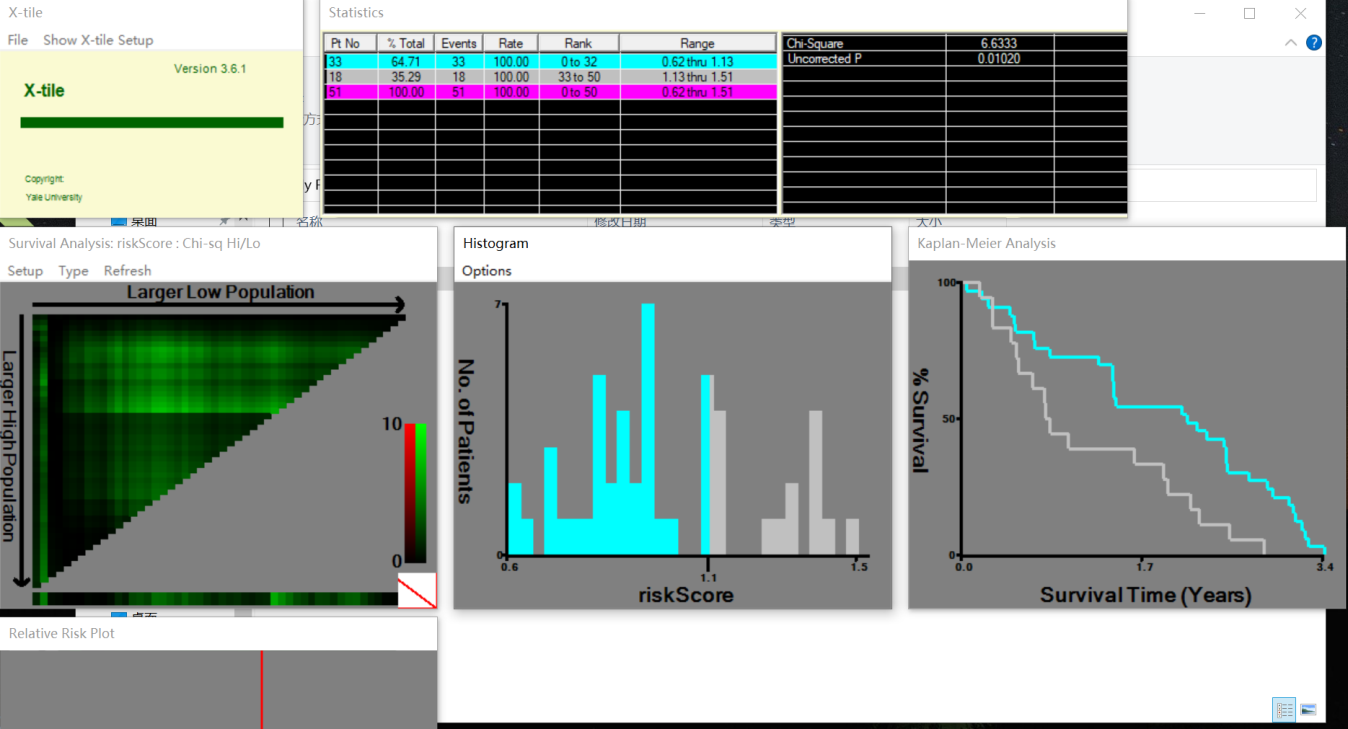
**

**Figure S5** Estimation of the best cutoff value for the GSE19061 risk scores determined by X-tile software. When 1.13 was set as the cut off value, the overall survival of patients in these two groups are significantly different (*P*=0.01).


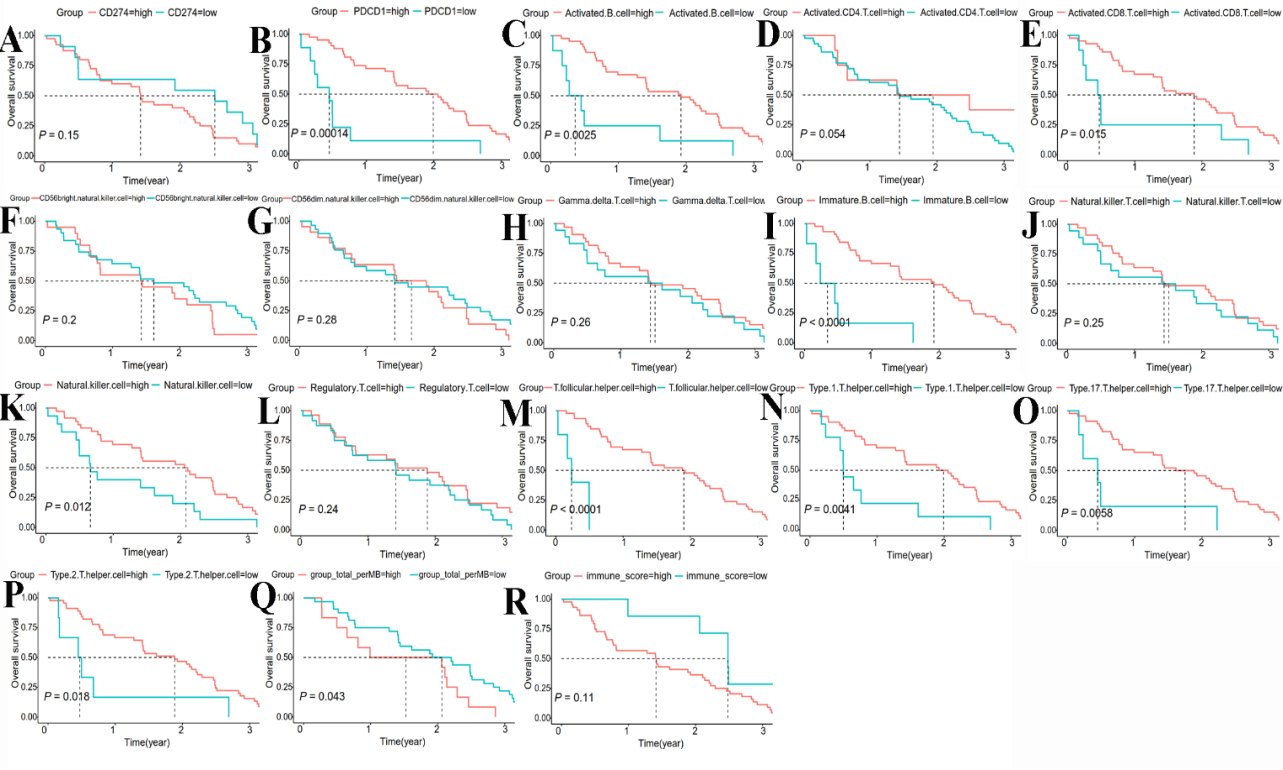


**Figure S6** Kaplan-Meier analysis of **(A)** CD274, **(B)** PDCD1, **(C)** Activated B cell, **(D)** Activated CD4 T cell, **(E)** Activated CD8 T cell, **(F)** CD56bright natural killer cell, **(G)** CD56dim natural killer cell, **(H)** Gamma delta T cell, **(I)** Immature B cell, **(J)** Natural killer T cell, **(K)** Natural killer cell, **(L)** Regulatory T cell, **(M)** T follicular helper cell, **(N)** Type 1 T helper cell, **(O)** Type 17 T helper cell, **(P)** Type 2 T helper cell, **(Q)** tumor mutant burden, **(R)** score of immune gene signature of GSE91061. There is statistical significance (*P*<0.05) between survival analysis and **(B)** PDCD1 expression, **(C)** Activated B cell, **(E)** Activated CD8 T cell, **(I)** Immature B cell, **(K)** Natural killer cell, **(M)** T follicular helper cell, **(N)** Type 1 T helper cell, **(O)** Type 17 T helper cell, **(P)** Type 2 T helper cell and **(Q)** tumor mutant burden.

**
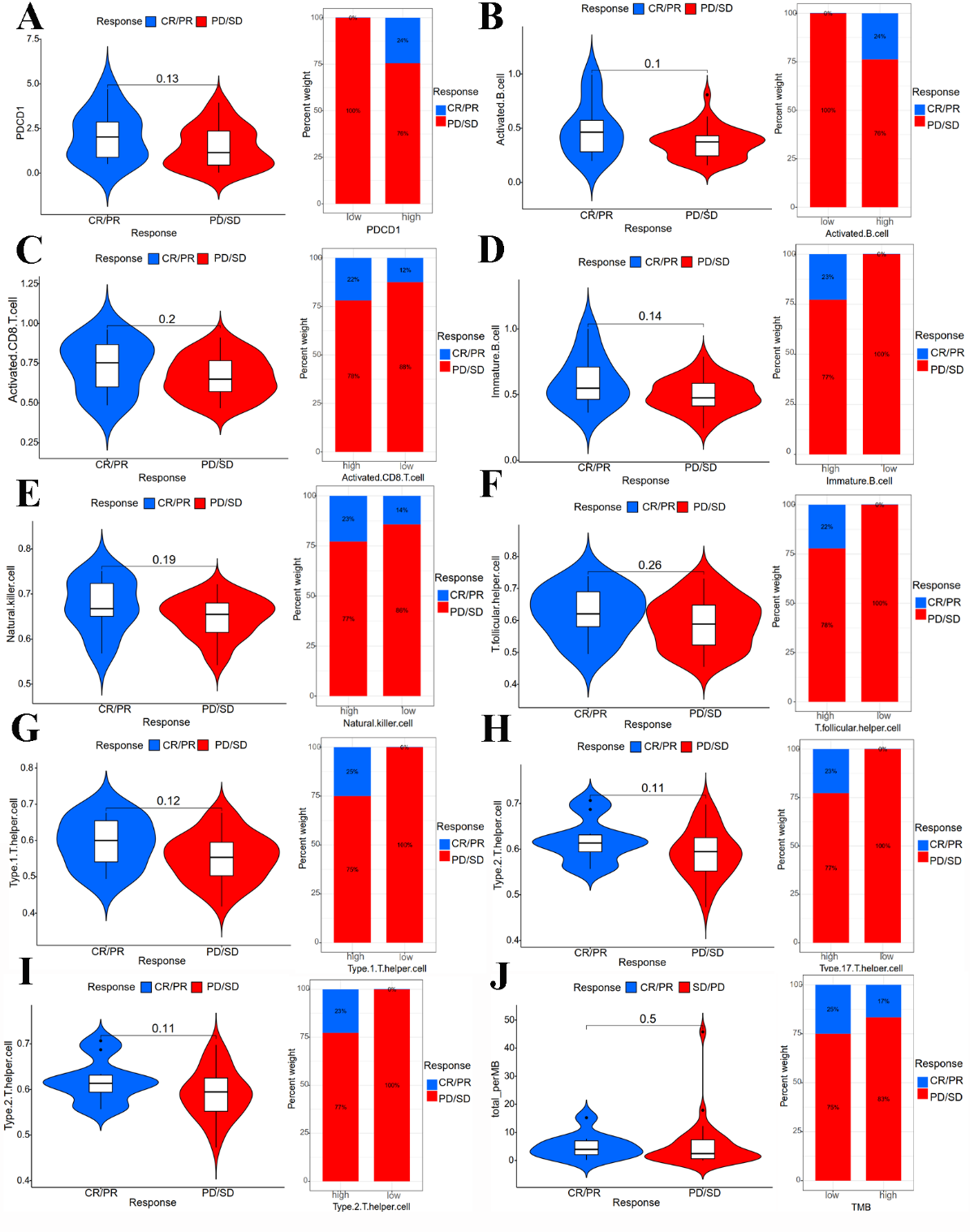
**

**Figure S7** The violin plot of scores of other biomarkers in different response groups of GSE19061 and the barplot of proportion of patients with response to anti-PD-1 immunotherapy in different biomarker level groups. The biomarkers included: **(A)** PDCD1 expression, **(B)** Activated B cell, **(C)** Activated CD8 T cell, **(D)** Immature B cell, **(E)** Natural killer cell, **(F)** T follicular helper cell, **(G)** Type 1 T helper cell, **(H)** Type 17 T helper cell, **(I)** Type 2 T helper cell and **(J)** tumor mutant burden. The patients with response to anti-PD-1 therapy tend to have a higher score of these 10 indexes than those patients without response to therapy.

**
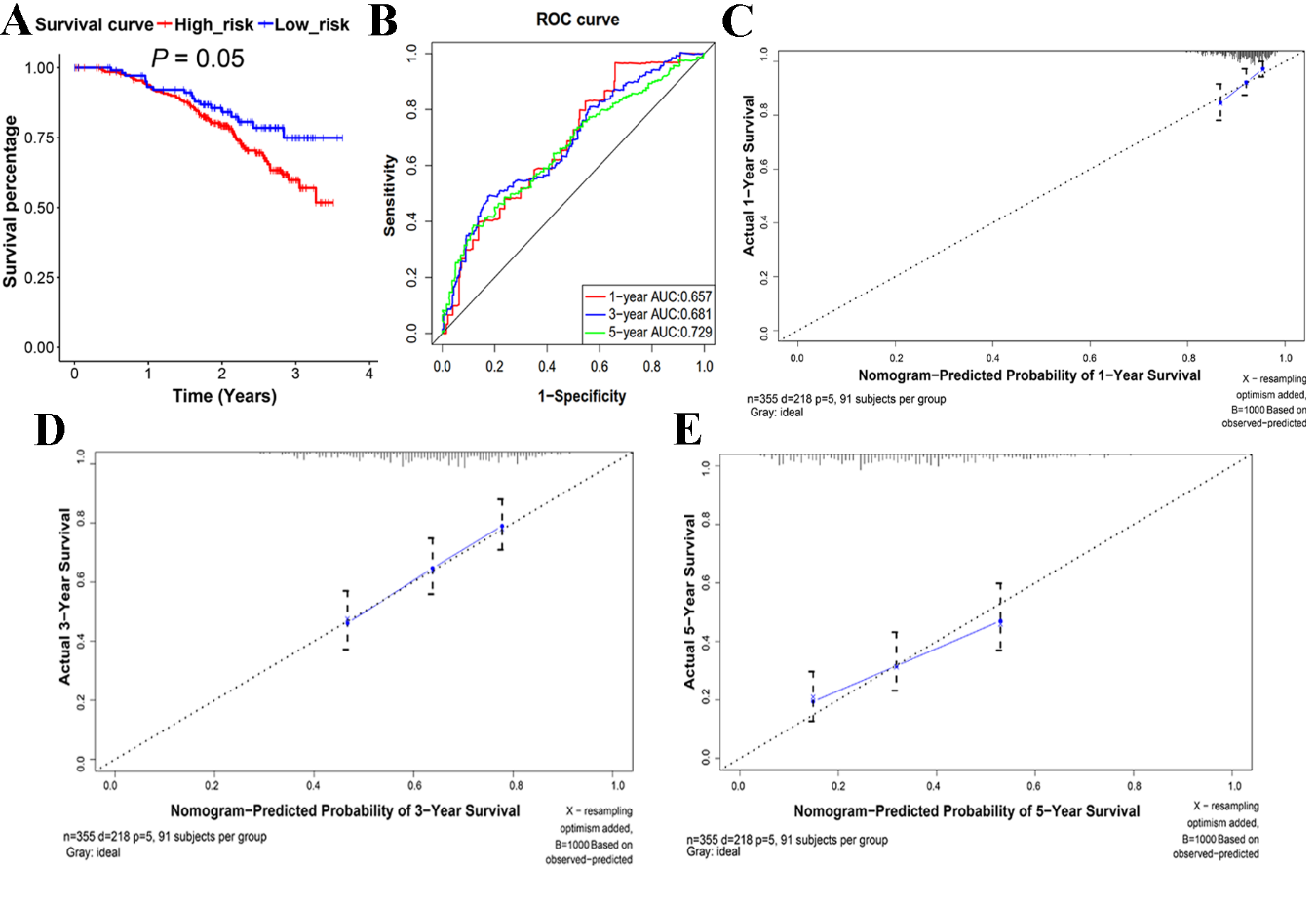
**

**Figure S8** Evaluation of the prognostic model in the antiangiogenesis dataset and construction of the nomogram. **(A)** Kaplan-Meier analysis of GSE140082. **(B)** ROC curve and AUC value of the prognostic model in GSE140082. Calibration plots for conformity between nomogram prediction and actual observation in terms of the **(C)** 1-, **(D)** 3-**,** and **(E)** 5- year survival rates. The gray dashed line is the reference line, and the blue solid curve is the curve fitting line. If these lines coincide, the capability of nomogram is convincing.


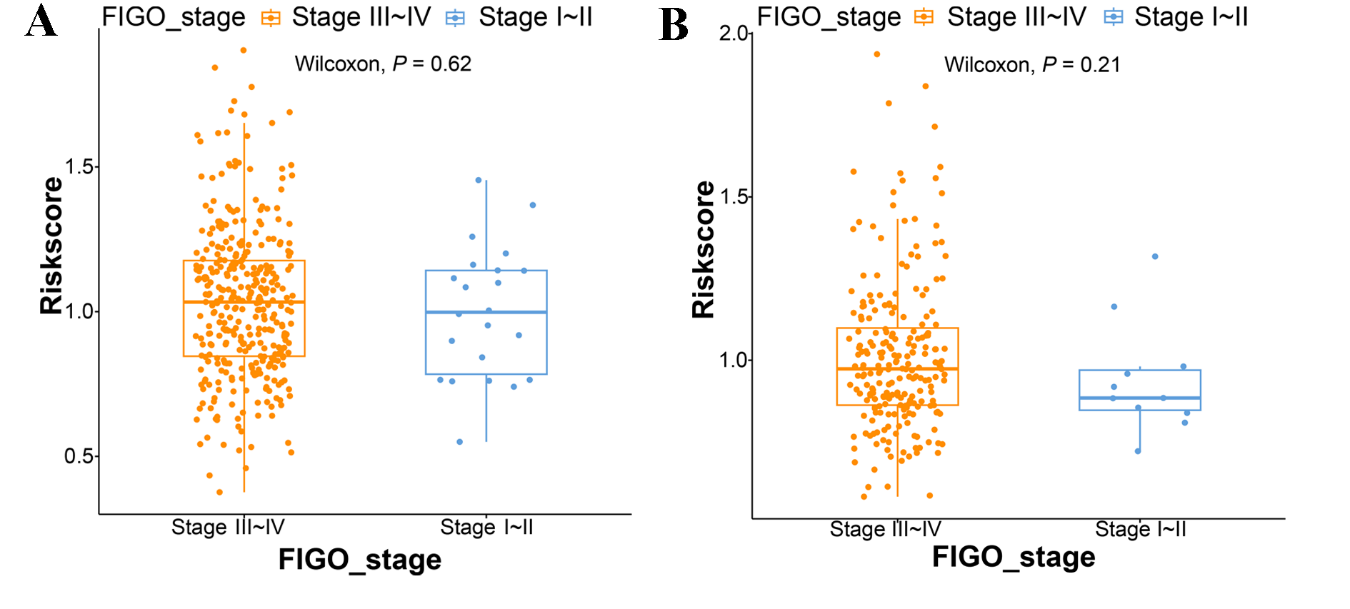


**Figure S9** Comparison of risk score in different FIGO stage，which means the condition on metastasis of SOC, in **(A)** TCGA cohort and **(B)** GSE14764 and GSE53963.
